# Towards improved accuracy of Hirshfeld atom refinement with an alternative electron density partition

**DOI:** 10.1107/S2052252524011242

**Published:** 2025-01-01

**Authors:** Michał Chodkiewicz, Krzysztof Woźniak

**Affiliations:** a Biological and Chemical Research Centre, Department of Chemistry University of Warsaw Żwirki i Wigury 101 Warszawa02-089 Poland; ESRF, France

**Keywords:** Hirshfeld atom refinement, alternative electron density partition, exponential Hirshfeld atom refinement, hydrogen atom parameters, quantum crystallography

## Abstract

Hirshfeld atom refinement (HAR) is generally the chosen method for obtaining accurate hydrogen atom parameters from X-ray diffraction data. Still, determination can prove challenging, especially in the case of atomic displacement parameters (ADPs). We demonstrate that such a situation can occur when the ADP values of the bonding partner of the hydrogen atom are not determined accurately. Atomic electron densities partially overlap and inaccuracies in the bonding neighbour ADPs can be partially compensated for with modifications to the hydrogen ADPs. We introduce a modified version of the original Hirshfeld partition: the exponential Hirshfeld partition, parameterized with an adjustable parameter (*n*) to allow control of the overlap level of the atomic electron densities which, for *n* = 1, is equivalent to the Hirshfeld partition. The accuracy of the HAR-like procedure using the new partition (expHAR) was tested on a set of organic structures using B3LYP and MP2 electron densities. Applying expHAR improved the hydrogen atom parameters in the majority of the structures (compared with HAR), especially in cases with the highest deviations from the reference neutron values. *X*—H bond lengths and hydrogen ADPs improved for 9/10 of the structures for B3LYP-based refinement and 8/9 for MP2-based refinement when the ADPs were compared with a newly introduced scale-independent similarity measure.

## Abstract 2

We demonstrate that applying the alternative electron density partition in a Hirshfeld atom refinement may significantly improve the accuracy of hydrogen atom parameters. The new partition leads to less overlapping atomic densities. As a result, hydrogen atom parameters are less dependent on the structural parameters of their neighbours and their inaccuracies.

## 1. Introduction

Hirshfeld atom refinement (HAR) is a method used for crystal structure determination from X-ray diffraction data (Jayatilaka & Dittrich, 2008 ▸; Capelli *et al.*, 2014 ▸). It uses atomic form factors based on atomic electron densities from quantum mechanical calculations for the system of interest. The Hirshfeld atom partitioning (Hirshfeld, 1977 ▸) of the electron density is applied to split the density into atomic contributions. HAR enables the determination of structural parameters much more accurately than the commonly used independent atom model (IAM), which is based on spherically symmetric atomic densities. The improvement is especially notable in the case of hydrogen atom parameters (Woińska *et al.*, 2016 ▸). Since X-rays are mostly scattered by electron density and hydrogen atoms have only a single electron, their structural parameters are relatively difficult to measure accurately as they are sensitive to small modifications of the model of electron density. IAM neglects the effects related to chemical bonding and interatomic interactions and provides quite poor bond lengths for hydrogen atoms and poor descriptions of hydrogen atomic displacement parameters (ADPs). The inclusion of information concerning the asphericity of atomic electron density usually greatly improves these descriptions. This was first demonstrated using a method based on multipole expansion of the atomic densities in terms of the Hansen–Coppens model (Hansen & Coppens, 1978 ▸). It turned out that parameters of the atomic densities can be transferred between atoms of the same type in different structures (Brock *et al.*, 1991 ▸; Pichon-Pesme *et al.*, 1995 ▸; Koritsanszky *et al.*, 2002 ▸). This led to the development and application of databases of transferable multipole model parameters: ELMAM2 (Zarychta *et al.*, 2007 ▸; Domagała *et al.*, 2012 ▸; Nassour *et al.*, 2017 ▸), Invariom (Dittrich *et al.*, 2004 ▸, 2005 ▸, 2006 ▸, 2013 ▸), UBDB (Volkov *et al.*, 2007 ▸; Dominiak *et al.*, 2007 ▸; Jarzembska & Dominiak, 2012 ▸; Kumar *et al.*, 2019 ▸) and its successor MATTS (Jha *et al.*, 2022 ▸). The corresponding model is called the transferable aspherical atom model (TAAM). TAAM-based hydrogen atom parameters are much more accurate than those obtained using IAM (*e.g.* Bąk *et al.*, 2011 ▸; Jha *et al.*, 2020 ▸).

Further improvements were achieved with HAR, which relies on the calculation of the electron density for the system of interest. This is derived from the fact that HAR does not rely on transferability, can take into account the effects of intermolecular interactions and is usually based on a much more flexible model of the total electron density. Intermediate to HAR and TAAM are methods that use the Hirshfeld electron density partition and, like TAAM, utilize the idea of transferability: (1) HAR-ELMO (Malaspina *et al.*, 2019 ▸) couples HAR with libraries of extremely localized orbitals (Meyer & Genoni, 2018 ▸), (2) HAR employing fragmentation techniques in quantum mechanical calculations (Bergmann *et al.*, 2020 ▸; Chodkiewicz *et al.*, 2022*a* ▸) and (3) a database of transferable atomic densities obtained with the Hirshfeld partition (Koritsanszky *et al.*, 2011 ▸; Chodkiewicz *et al.*, 2024 ▸). Though all of the above show accuracies similar to HAR, they are, in principle, less accurate than HAR due to applied approximations, but are also less time consuming.

Some systems are still challenging for HAR [*e.g.* those including hydrogen atoms bonded to heavy-element atoms (Woińska *et al.*, 2021 ▸, 2023 ▸)]. It was possible to obtain very good agreement between HAR- and neutron-measurement-derived hydrogen atom parameters for a number of organic molecules, for example, in urea (Chodkiewicz *et al.*, 2020 ▸; Ruth *et al.*, 2022 ▸) the N—H bond length could deviate from the neutron measurements by as little as 5 mÅ, although in certain cases the agreement was much worse, *e.g.* 40 mÅ for the O—H bonds in *N*-acetyl-l-4-hy­droxy­proline monohydrate (Chodkiewicz *et al.*, 2024 ▸), yet still much better than IAM. It seems that the parameters of carbon-bonded hydrogen atoms are more likely to be accurately derived with HAR than those of oxygen- or nitro­gen-bonded hydrogen atoms [figure 2 in Woińska *et al.* (2016 ▸)]. Hydrogen atoms in COOH and OH groups appear to be prone to elongation of their thermal ellipsoids along bonding directions even if such a feature was not present in the neutron measurement structure.

Therefore, while HAR has significantly improved the structural description of hydrogen atoms compared with the spherical model, it is still not a reliable replacement for neutron measurements, even in the case of systems that contain only light elements.

The possibility of improving HAR results with the help of techniques that allow for the estimation of hydrogen atom ADPs, such as SHADE3 (Madsen & Hoser, 2014 ▸), TLS+INV (Lübben *et al.*, 2015 ▸) and Normal Mode Refinement (NoMoRe) (Hoser & Madsen, 2016 ▸, 2017 ▸) has been tested. Including estimated hydrogen ADPs has additionally been shown to be necessary (Dittrich *et al.*, 2017 ▸) in cases where the hydrogen atom ADPs become non-positive definite in free refinement. ADPs obtained with such methods appear to be more accurate than those obtained directly with HAR (Woińska *et al.*, 2024 ▸; Wanat *et al.*, 2021 ▸), yet SHADE3 estimates were not always accurate (Malaspina *et al.*, 2020 ▸), and NoMoRe was relatively expensive computationally and not applicable to disordered structures (in the current form). Fixing hydrogen ADPs to the value obtained with such methods did not improve *X*—H bond lengths (Woińska *et al.*, 2024 ▸; Malaspina *et al.*, 2020 ▸).

In this work, we focus on exploring the possibility of improving HAR. There are various ways refinement can be performed, including choice of quantum chemistry method, basis set and representation of intermolecular interactions [*e.g.* with multipoles mimicking the crystal field (Jayatilaka & Dittrich, 2008 ▸)] or using the periodic wavefunction (Wall, 2016 ▸; Ruth *et al.*, 2022 ▸). Finally, HAR can be generalized by the application of electron density partitions other than the Hirshfeld partition (Chodkiewicz *et al.*, 2020 ▸). All these aspects of HAR have been at least partially explored. It was observed that changes in various settings lead to systematic changes in the refined structure, yet no clear path to improve results has been found.

For example, application of the Hartree–Fock (HF) method usually leads to longer polar bonds to hydrogen than DFT. This phenomenon was studied by Landeros-Rivera *et al.* (2023 ▸) who linked the effect with the amount of Hartree–Fock exchange (*E*_x_^HF^) in the density functional (the larger the fraction of *E*_x_^HF^, the longer the polar *X*—H bonds). Though the HF method usually provides larger agreement factors (*R* factors) than DFT methods, it also seems to supply more accurate bond lengths. Among DFT functionals, those with about 25% admixture of *E*_x_^HF^ gave the lowest *R* factors. Post-HF methods were also applied to HAR but no clear advantage was observed over less expensive methods (Wieduwilt *et al.*, 2020 ▸; Chodkiewicz *et al.*, 2020 ▸). In the case of a basis set choice, it is known that cc-pVDZ gives systematically different results than the larger basis set cc-pVTZ (cc-pVDZ gives smaller ADPs). It is however quite difficult to judge which ADPs are best, as the ADPs for heavier atoms derived from neutron and X-ray measurements are sometimes quite different (usually in terms of size rather than directionality) and therefore it is sometimes not clear how to choose reference values for assessment of hydrogen ADP accuracies. What appears to be clear in the case of quantum chemistry methods is that accounting for the effects of intermolecular interactions improves the accuracy of HAR, especially in the case of systems with strong hydrogen bonds. Methods that involve the calculation of periodic wavefunctions appear to be very promising (Ruth *et al.*, 2022 ▸) in this respect.

Unlike the choice of quantum chemistry method and basis set, the research does not suggest which partition should give better results in HAR, except for one study that explicitly tested some of the partitions in an HAR-like refinement (Chodkiewicz *et al.*, 2020 ▸). However, no clear improvement over the Hirshfeld partition was observed. Results of similar accuracy were obtained with the Hirshfeld partition and iterative stockholder partition, even though the difference in atomic charge on the hydrogen atom reached values as large as 0.4 e (*i.e.* more than 40% of electrons in the atom).

Although obtaining accurate hydrogen atom parameters with HAR does not require high-resolution data, it is expected that higher resolutions should bring better results. Sometimes this leads to a reduced accuracy [figure 2 in Woińska *et al.* (2016 ▸)], especially for O—H bonds. C—H bond length accuracies appear to be much more stable to increasing data resolution. Furthermore, these parameters depend on the weighting scheme applied (Kleemiss *et al.*, 2021 ▸), sometimes quite substantially in our experience.

Multiple factors influence the accuracy of HAR, which complicates the search for the optimal method to perform it. In this study, we try to shed light on some of the so-far unexplained trends and phenomena observed in HAR to make the search for optimal settings for HAR and HAR-derived methods easier. Such settings are also important for approaches relying on the transferability of atomic densities such as TAAM and THAM, since in this case the choice of method used for the generation of the corresponding databanks of atomic electron densities also influences the accuracy of the methods (Chodkiewicz *et al.*, 2024 ▸).

## 2. Test systems and methods

### 2.1. Test systems

**Figure 1:**
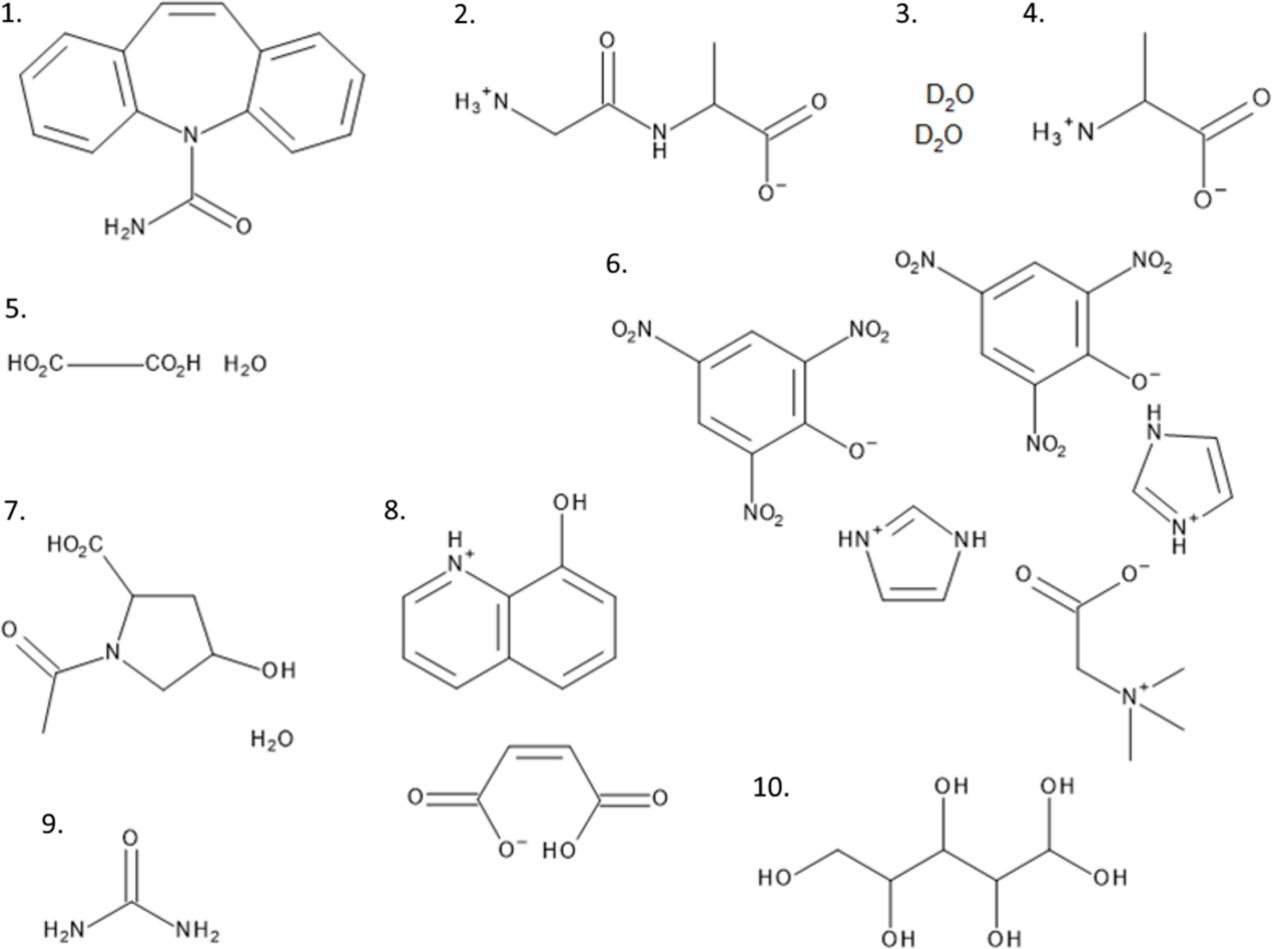
The test systems, symmetry independent molecules/ions shown: (1) carbamazepine, (2) Gly-l-Ala, (3) ice VI, (4) l-Ala, (5) Oxa·2H_2_O, (6) BIPa, (7) NAC·H_2_O, (8) 8HQ HM, (9) urea and (10) xylitol.

Most of the datasets used in this work contain high-resolution data because there were matching neutron structures available for such types of data. High-resolution datasets are commonly used in HAR development, which has consequences for method assessment, discussed later. We have chosen the following datasets (see Fig. 1 ▸):

(1) Carbamazepine, form III – X-ray data (*d*_min_ = 0.42 Å) and neutron structure from Sovago *et al.* (2016 ▸), *T* = 100 K.

(2) Gly-l-Ala – X-ray data (*d*_min_ = 0.65 Å) and neutron structure from Capelli *et al.* (2014 ▸), *T* = 150 K.

(3) Ice VI, a disordered form of heavy ice (D_2_O) – X-ray data (*d*_min_ = 0.62 Å) from Chodkiewicz *et al.* (2022 ▸*b*), *p* = 1.15 GPa, room temperature, in-house source data; neutron structure from Kuhs *et al.* (1989 ▸), *p* = 0.85 GPa, *T* = 296 K.

(4) l-Alanine (l-Ala) – X-ray data (*d*_min_ = 0.46 Å) from Destro *et al.* (1988 ▸), neutron structure from Malaspina *et al.* (2019 ▸), *T* = 23 K.

(5) Oxalic acid dihydrate (Oxa·2H_2_O) – X-ray data (dataset oxa7, *d*_min_ = 0.43 Å) and neutron structure from Kamiński *et al.* (2014 ▸), *T* = 100 K.

(6) BIPa, a co-crystal of a betaine zwitterion, two imidazolium cations and two picrate anions – X-ray data (*d*_min_ = 0.42 Å; Overgaard *et al.*, 1999 ▸)] from Fugel *et al.* (2018 ▸) and neutron structure from Jørgensen *et al.* (2014 ▸), *T* = 100 K.

(7) *N*-acetyl-l-4-hy­droxy­proline monohydrate (NAC·H_2_O) – X-ray data (*d*_min_ = 0.49 Å) and neutron structure from Lübben *et al.* (2014 ▸), *T* = 9 K.

(8) 8-hy­droxy­quinolinium hydrogen maleate (8HQ HM) – X-ray data (*d* = 0.43 Å) from Malaspina *et al.* (2020 ▸) *T* = 15 K; neutron data from Malaspina *et al.* (2017 ▸), *T* = 12 K.

(9) Urea – X-ray data (*d*_min_ = 0.36 Å) from Birkedal *et al.* (2004 ▸) and neutron structure from Swaminathan *et al.* (1984 ▸), *T* = 123 K.

(10) Xylitol – X-ray data (*d*_min_ = 0.42 Å) from Madsen *et al.* (2004 ▸) and neutron structure from Madsen *et al.* (2003 ▸), *T* = 122 K.

### 2.2. Refinements

HAR and a modified version of HAR with alternative partitions of the electron density (described later) were performed using a locally modified version of *Olex2* (Dolomanov *et al.*, 2009 ▸; Bourhis *et al.*, 2015 ▸). A program based on a development version of the DiSCaMB library (Chodkiewicz *et al.*, 2018 ▸) was used to generate files with atomic form factors in .tsc format (Kleemiss *et al.*, 2021 ▸; Midgley *et al.*, 2019 ▸). Such files were then imported into *Olex2* and used in the refinement conducted with *olex2.refine*. The HAR procedure was repeated until the maximum shift of a parameter divided by its standard deviation was lower than 0.1. Though we think the threshold is already sufficient, a tighter threshold (0.01) is commonly used in HAR (Capelli *et al.*, 2014 ▸).

HAR involves the calculation of a wavefunction. The most popular approach, also applied here, is based on the calculation of a wavefunction for a molecular system (in contrast to a periodic one). The wavefunction is calculated for molecular species extracted from the crystal structure. For the structures with *Z*′ = 1 (where *Z*′ denotes the number of symmetry-independent molecules in the asymmetric unit), the molecule from the asymmetric unit is used. For the other systems, the following subsystems have been used: Oxa·2H_2_O – oxalic acid with the two closest water molecules (one subsystem); BIPa – five separate subsystems (*i.e.* wavefunctions calculated separately) corresponding to the five chemical units in the asymmetric unit; NAC·H_2_O – the two chemical units treated as one subsystem; and 8HQ HM – wavefunctions for the two ions were calculated independently. The ice VI case is more complicated due to disorder; here, calculations of atomic form factors involved averaging atomic electron densities from multiple possible local conformations of a water molecule. Wavefunctions were calculated for clusters of five water molecules (the ‘central’ one and its closest neighbours). Six such clusters were used. The configurations of the neighbouring molecules were chosen randomly from a larger number of possible configurations that fulfil the so-called ice rules (Bernal & Fowler, 1933 ▸). Other possible representations would differ only in the position of the hydrogen atom that is not directly involved in the hydrogen bond to the central molecule. The electron densities of the atoms in the central molecule were used for form-factor calculations.

Distributed multipoles up to dipoles were used to represent interactions in a crystal, for all structures except ice VI. The multipoles were located on chemical units up to 8 Å apart from the one for which the wavefunction is calculated, and they were calculated using the same electron density partition as the one used in the refinement.

The majority of quantum mechanical calculations were performed using DFT with the B3LYP functional and MP2 using the cc-pVTZ basis set. A few additional refinements used the HF method with the cc-pVTZ and cc-pVDZ basis sets. *ORCA* (version 5.0; Neese, 2012 ▸, 2022 ▸) was used for DFT and HF calculations, and *Gaussian16* (revision C. 01; Frisch *et al.*, 2016 ▸) was used for MP2 calculations.

Form factors were calculated via numerical integration with a 590-point Lebedev–Laikov grid for angular integration (Lebedev & Laikov, 1999 ▸) and a 75-point Mura–Knowles (Mura & Knowles, 1996 ▸) radial integration grid (the same grid size was used for all atoms).

### 2.3. Statistics

The accuracy of the refined structural parameters was assessed by comparison with reference neutron structures.

#### 2.3.1. Bond lengths

The accuracy of the bond lengths was described with an average absolute difference between the value from the X-ray refinement and the reference one:
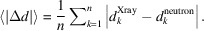
Similarily defined was the (non-absolute) average difference, 〈Δ*d*〉, which was useful for detecting trends in bond lengths.

#### 2.3.2. ADPs

ADPs for non-hydrogen atoms from neutron and X-ray measurements sometimes differed systematically. Comparison of hydrogen ADPs may involve some corrections that account for differences, *e.g.* a scaling procedure introduced by Blessing (1995 ▸). However, it did not fully solve the problem since the parameters of the scaling procedure were derived from ADPs of non-hydrogen atoms and, in principle, they may differ from those that should be used for hydrogen atoms.

To mitigate the problem of different scales for X-ray and neutron ADPs, a similarity indicator, MSD_corr_, was introduced that is independent of the scale of the ADPs and compares the directionality of atomic displacements. It is defined in an analogous way as a correlation coefficient, though it misses statistical interpretation. It measures the ‘correlation’ of atomic mean square displacements (MSDs). The MSD of an atom is defined as the mean value of the square of the projection of its displacement onto the chosen direction given by a unit vector **n** (Nelmes, 1969 ▸), related to ADP tensor **U** by the simple relation: MSD**_n_**(**U**) = **n**^T^**Un**. A root mean square displacement RMSD**_n_** = (MSD**_n_**)^1/2^ was used to construct peanut plots (Hummel *et al.*, 1990 ▸). MSD_corr_ is defined similarly to a Pearson correlation coefficient between
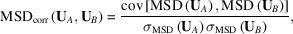
where cov is the ‘covariance’, defined by
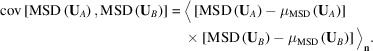
The expression in angle brackets is averaged over all possible displacement directions **n**, μ_MSD_ is the averaged MSD (also averaged over all possible **n**) and is equal to the equivalent isotropic ADP (*U*_eq_), and 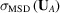 is the standard deviation of MSD(**U**_*A*_): 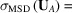

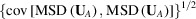
. Since MSD_corr_ is a correlation coefficient, it can take values between −1 and 1. It is undefined for isotropic ADPs. Adding an identity matrix multiplied by a number to the ADP tensor did not change MSD_corr_. To illustrate the properties of the index, its values for a few combinations of the diagonal ADP tensors are given: MSD_corr_[diag(2,1,1), diag(1,2,2)] = −1, MSD_corr_[diag(2,1,1), diag(1,2,1)] = −0.5, MSD_corr_[diag(2,1,1), diag(2,2,1)] = 0.5, MSD_corr_[diag(2,1,1), diag(3,1,1)] = 1, MSD_corr_[diag(3,3,2), diag(3,1,2)] = 0. The calculation of MSD_corr_ was performed by numerical integration over a unit sphere using a Lebedev–Laikov grid with 5810 points (however, an analytical expression was also derived, see the supporting information).

Other scale-independent ADP similarity measures exist, *e.g.* the modified correlation coefficient (Kondrashov *et al.*, 2007 ▸) and the normalized correlation coefficient (Merritt, 1999 ▸). They are closely related to the correlation coefficient (CC) introduced by Merritt (1999 ▸) which, in turn, is closely related to the *S*_12_ index [*S*_12_ = 100(1 − CC)] introduced by Whitten & Spackman (2006 ▸). Another ‘shape’ similarity measure is the angle between the longest principal axes of the ellipsoids (Yang *et al.*, 2009 ▸; Mroz *et al.*, 2021 ▸). MSD_corr_ was chosen in this work because it is relatively easy to interpret the numerical values of the index since it is an analog of the correlation coefficient.

In addition to MSD_corr_, the rescaled overlapping coefficient (Chodkiewicz *et al.*, 2024 ▸) was used. It is based on the overlapping coefficient (Inman & Bradley, 1989 ▸) which, for one-dimensional probability distributions, is the overlapping area of the distribution functions. The pair distribution functions (PDFs) [*p*_1_(**u**), *p*_2_(**u**)] of atomic displacements (**u**), corresponding to the compared ADPs, can be written as
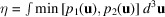
or alternatively with the help of 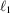, a norm of the difference of PDFs as 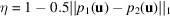. The rescaled version 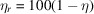 can be seen as the percentage difference between PDFs. This similarity index is scale-dependent so the potential difference in the scale of ADPs from neutron and X-ray measurements would affect its value.

A ratio of equivalent isotropic ADPs (*U*_eq_) was used to compare the extent of atomic displacements.

The computer program *compare_adps* for computing the ADP similarity indices is freely available at https://www.discamb.org/download.html; it is based on the DiSCaMB library (Chodkiewicz *et al.*, 2018 ▸).

## 3. Insight from the analysis of ADPs

An assessment of HAR as a refinement method usually involves a comparison of HAR-derived hydrogen atom structural parameters, mainly bond lengths and ADPs.

A bond-length comparison is relatively straightforward but its interpretation may be hindered by a cancellation of errors which may occur when two sources of error lead to the opposite effects on the bond lengths. For example, HAR for xylitol performed with the B3LYP functional using the cc-pVTZ basis set and distributed multipoles (to mimic the effect of the surrounding molecules) leads to O—H bond lengths which differ from neutron diffraction results on average by 17 mÅ. In comparison, HAR with HF and a smaller basis set (cc-pVDZ) reduces the error to 4.5 mÅ. It might be surprising that the method that neglects correlation (HF) combined with a smaller basis set gives better results. But when the *R* factors are compared, it turns out that B3LYP-based HAR performs better (*R*_1_ = 1.56) than HF-based refinement (*R*_1_ = 1.69). Higher *R* factors but more accurate bond lengths are rather typical for HF-based refinements (*e.g.* Chodkiewicz *et al.*, 2024 ▸; Landeros-Rivera *et al.*, 2023 ▸; Wieduwilt *et al.*, 2020 ▸).

**Figure 2:**
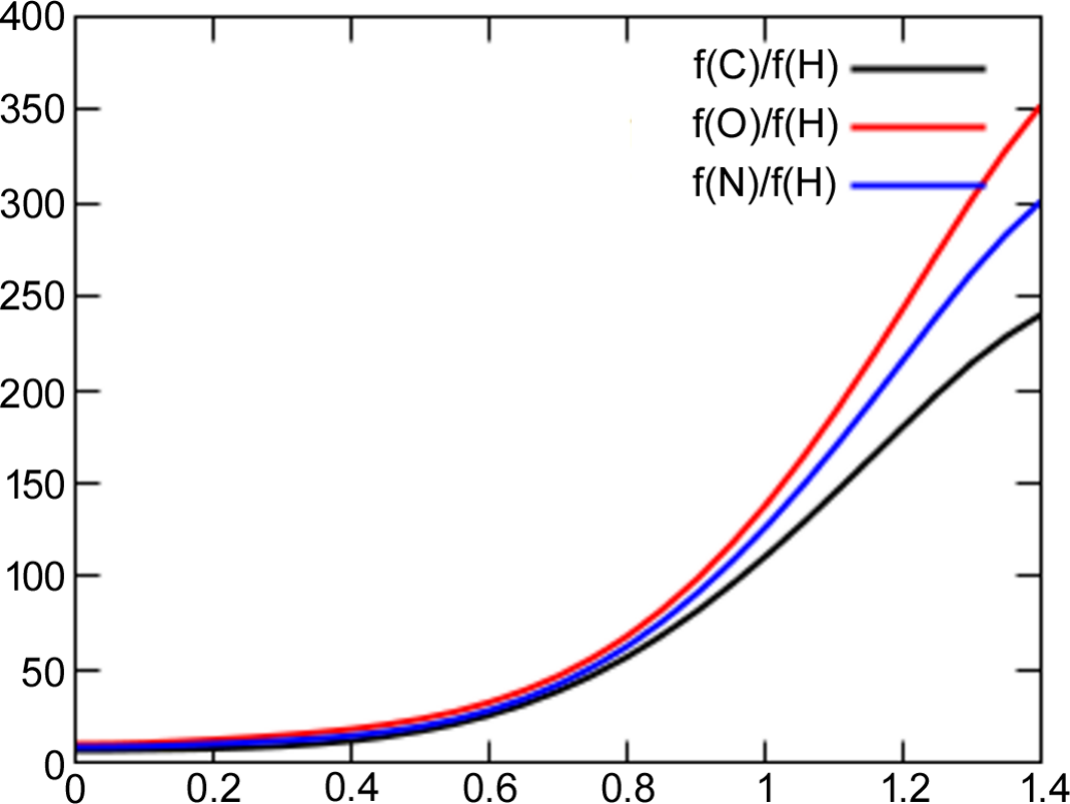
Ratio of non-hydrogen (C, N, O) to hydrogen atom form factors in urea as a function of sin(θ)/λ (Å^−1^).

Clearly, analysis of the bond-length statistics alone might lead to incorrect conclusions. Therefore, it is important to also include ADPs in the analysis. Comparison of hydrogen ADPs from neutron and X-ray measurements is hindered by the fact that already the ADPs for heavier atoms sometimes differ considerably. There is no commonly accepted approach to tackle this problem. The differences between ADPs of non-hydrogen atoms can be approximately expressed in terms of simple adjustment schemes (Blessing, 1995 ▸, and references therein). Parameters of such a transformation fitted for non-hydrogen atoms can be applied to hydrogen ADPs (Blessing, 1995 ▸). These transformations can be useful (*e.g.* for deriving fixed hydrogen atom parameters in the X-ray analysis of electron density distribution) but sometimes they are also used to rescale ADPs before comparing neutron- and X-ray-derived structures. Such analysis should be applied with caution, since hydrogen atoms’ contribution to high-angle scattering is very small. Their form factors are even a few hundred times smaller than those of heavier elements (like C, N and O) at the maximum resolution of some of the measurements used for HAR-testing purposes, see *e.g.* the form-factor ratio for aspherical form factors of atoms in urea (Fig. 2 ▸, calculation details are provided in the supporting information). Therefore, the effect of systematic errors on the hydrogen and non-hydrogen ADPs can be quite different and the application of a transformation fitted for non-hydrogen to hydrogen ADPs could be ineffective in correcting for systematic errors.

**Figure 3:**
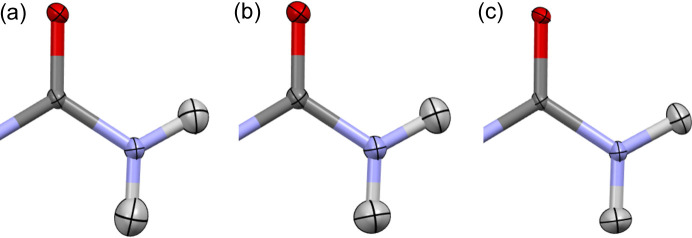
Amide group in carbamazepine, derived with (*a*) HAR with all data included (*d*_min_ = 0.42 Å), (*b*) HAR with limited data (*d*_min_ = 0.8 Å) and (*c*) neutron refinement.

The simplest, isotropic correction, 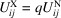, uses only one parameter. The parameter *q* sometimes considerably changes when high-resolution X-ray data are omitted, suggesting that non-hydrogen ADPs determined with high-resolution data included (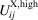) can be systematically larger/smaller than those determined with limited resolution (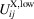). We can compare them using the same scheme: 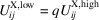. For example, for the high-resolution carbamazepine structure [sin(θ)/λ up to 1.19 Å^−1^ (Sovago *et al.*, 2016 ▸)], the *q* parameter is 0.865 (HAR with B3LYP, for low-resolution data, *d*_min_ = 0.8 Å), indicating that the use of high-resolution data leads to smaller ADPs in this case. Low-resolution refinement seems to be more similar to the neutron structure, which is especially visible in the case of the amide group (Fig. 3 ▸). MSD_corr_ for nitrogen-bonded hydrogen atoms increased from 0.87 to 0.98 and, in addition, 〈|Δ*d*|〉 dropped from 20 to 9 mÅ. In the case of carbon-bonded hydrogen atoms, the MSD_corr_ increased from 0.89 to 0.94 and 〈|Δ*d*|〉 stayed at the same level (5 mÅ).

Since hydrogen atoms’ contribution to the scattering factor is tiny at high resolution, we would not expect that the contribution plays an important role in the change of hydrogen ADPs with resolution. One of the possible explanations for the observed inaccuracy of hydrogen ADPs in carbamazepine is that non-hydrogen ADPs refined for high-resolution data are not optimal for low-resolution data (they differ in size as quantified by the scaling factor *q* = 0.865), but the inaccuracies can be partially compensated with modifications to the hydrogen ADPs.

**Figure 4:**
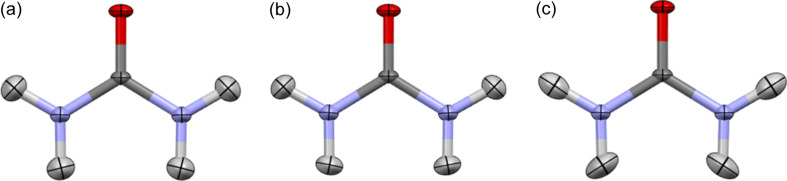
Urea structure derived from (*a*) HAR, (*b*) neutron experiment, (*c*) HAR with nitro­gen ADPs fixed to 90% value of the values from HAR refinement.

This hypothesis was tested with a dataset that provides a very good match between the neutron and X-ray measurement structures and also contains the urea amide group. In this case, the *q* parameter for the low-/high-resolution X-ray structure comparison is close to one (0.996). However, if nitro­gen ADPs in urea are fixed to 90% of their original value (equivalent to *q* = 0.9 for N), the hydrogen ADPs become quite different from those from neutron measurement (Fig. 4 ▸). Similarly, as in the case of carbamazepine refinement for high-resolution data, one of the hydrogens in the amide group is elongated along the bond direction.

**Figure 5:**
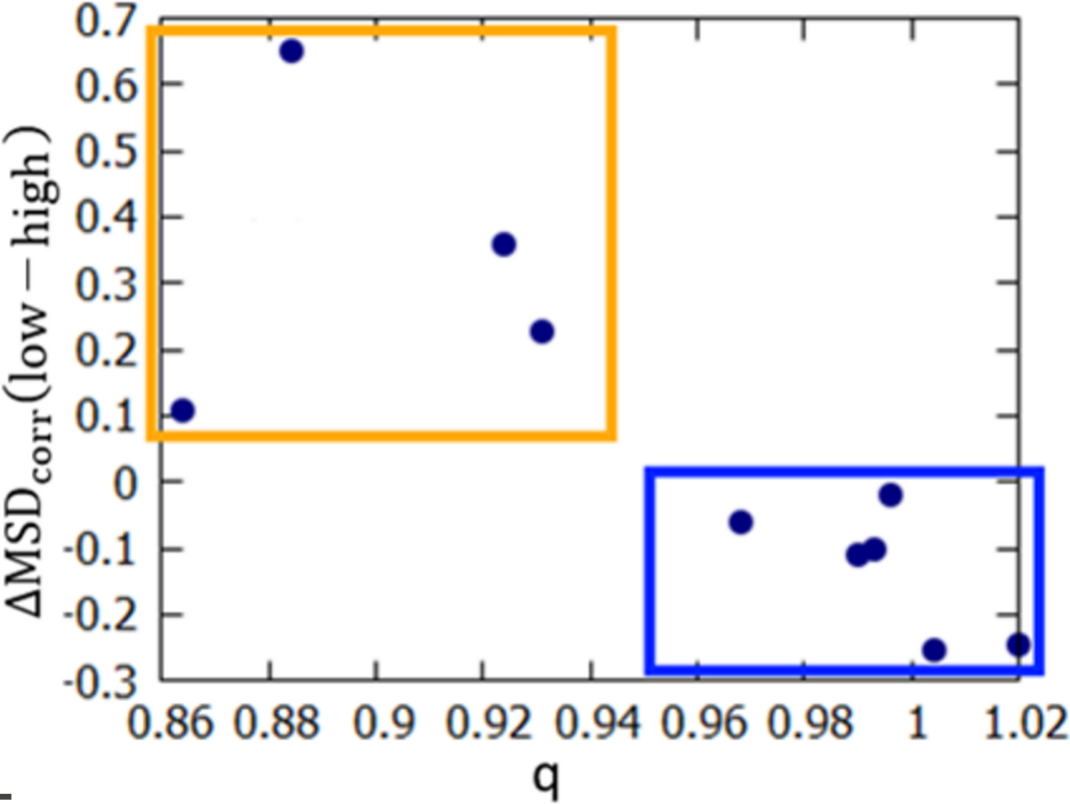
Change in polar hydrogen ADP accuracy (measured with average MSD_corr_) when the data resolution is limited to *d* = 0.8 Å plotted against the scale parameter *q* for scaling low-resolution refinement (*d*_min_ = 0.8 Å) non-hydrogen ADPs to values from high-resolution refinement. Limiting the resolution deteriorated the accuracy of the ADPs when parameter *q* was close to 1 (points inside the blue box) and improved otherwise (points inside the orange box).

For the systems tested, use of high-resolution data increased the accuracy of polar hydrogen ADPs (as measured with MSD_corr_) only when the scale factor *q* for non-hydrogen ADPs at low and high resolution is close to one (Fig. 5 ▸ and Table 1 ▸, low-resolution refinements were performed with *d*_min_ = 0.8 Å, results for B3LYP refinements are shown; MP2 refinements gave similar results, included in Section S10 of the supporting information). Otherwise, an increase in the resolution led to a decrease in the accuracy. This happened for four of the structures: carbamazepine, NAC·H_2_O, l-Ala and BIPa.

## 4. Electron density partition with adjustable interatomic overlap

**Figure 6:**
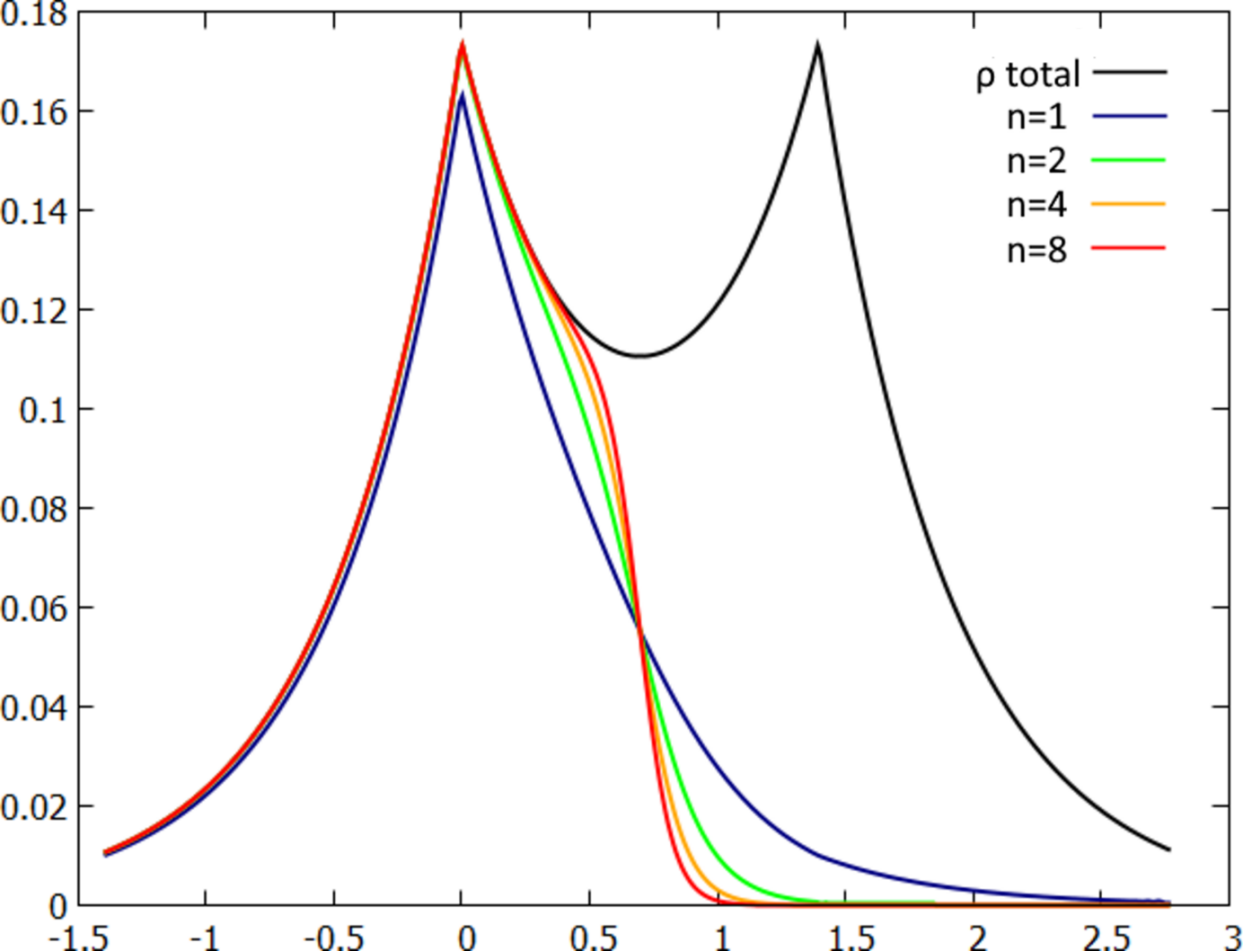
Dependence of atomic electron density on the parameter *n* in the exponential Hirshfeld partition for the model system: 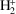 ion. Black line – total electron density, other lines – atomic electron densities for various values of the parameter *n* in the exponential Hirshfeld partition.

Inaccuracies in hydrogen ADPs from HAR appear to be a function of the ADP inaccuracies of their bonding partners. Assuming that a larger overlap of atomic electron densities makes the influence of the bonding partner larger, the inaccuracy can be at least partially eliminated by lowering the overlap. For that purpose, we have constructed an electron density partition based on the original Hirshfeld partition, defined as
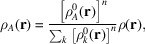
where 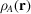 is the atomic electron density of atom *A*, 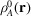 is the spherically averaged atomic density of the isolated atom *A* and ρ(**r**) is total electron density. For *n* = 1, this is the original Hirshfeld partition. Hereafter, the partition will be referred to as the exponential Hirshfeld partition and the corresponding refinement as expHAR(*n*), where *n* is an adjustable parameter of the partition (exponent). Choosing parameter *n* > 1 lowers the overlap of atomic electron densities. The effect is illustrated using a simple model of the 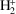 ion electron density, with the wavefunction ψ(*r*) = *N*[φ*A*(*r*) + φ*B*(*r*)], where φ*A* and φ*B* are hydrogen atom 1*s* orbitals (Fig. 6 ▸).

**Figure 7:**
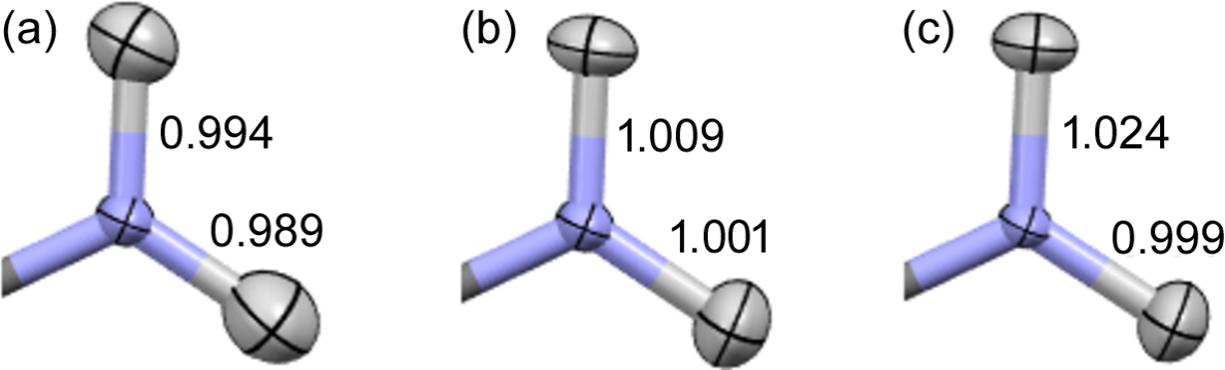
NH_2_ group in carbamazepine, derived using (*a*) HAR, (*b*) expHAR(2) and (*c*) neutron diffraction.

An application of expHAR(2) to carbamazepine (with the B3LYP/cc-pVTZ level of theory) brings a clear improvement of the nitrogen-bonded hydrogen atom parameters (Fig. 7 ▸). Bond lengths and the shape of the thermal ellipsoids are now closer to those from the neutron experiment.

Hydrogen atom ellipsoid elongation along the bond direction [as in Fig. 7 ▸(*a*)] seems much more common in the case of polar hydrogen atoms than in the case of carbon-bonded hydrogen atoms. This effect, as well as the smaller sensitivity of the C—H bond lengths and their ADPs to change of resolution, can be explained by a lower overlap of the atomic electron density. The overlap can be quantified with an overlap integral for the atomic densities:
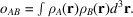
The values of this parameter for C—H, N—H and O—H pairs in selected structures are given in Table 2 ▸. The overlap can be ordered in the following way: O—H > N—H > C—H – larger overlap appears to correspond to more frequent problems with the description of hydrogen ADPs. The overlap is much smaller for the exponential Hirshfeld partition with an exponent of 2, which is more than 2× smaller than the overlap for the C—H pair for the Hirshfeld partition. The values of the overlap integral *o_AB_* were calculated using the wavefunction from HAR, *i.e.* the same geometry and wavefunction were used for both the Hirshfeld partition and the exponential Hirshfeld partition calculations.

Increasing the exponent in the exponential Hirshfeld partition usually leads to higher absolute values of atomic charges (see Section S11 of the supporting information). Increasing the exponent to infinity limits the overlap of atomic electron densities to zero and introduces boundaries between atoms. There are few other non-overlapping partitions of electron density known. One of the most prominent ones was developed within the so-called quantum theory of atoms in molecules (QTAIM) by Bader (1990 ▸). There is also a group of partitions based on the concept of Voronoi polyhedra (Voronoi, 1908 ▸) proposed *e.g.* by Politzer & Harris (1970 ▸) and Rousseau *et al.* (2001 ▸). Becke (1988 ▸) introduced a fuzzy version of such partition as part of a scheme for numerical integration in DFT. Similar to the case of the exponential Hirshfeld partition, the level of overlap of atomic electron densities can be controlled with a parameter (which is an integer number in this case). Becke partition was already applied in HAR-like refinement (Chodkiewicz *et al.*, 2020 ▸) but it led to less accurate results than other partitions tested in that paper.

Since atoms in crystals constantly move, diffraction experiments ‘see’ dynamic electron density. It is common to approximate it as a sum of dynamic atomic electron densities represented as static atomic densities smeared out by atomic motion/displacement. Such dynamic atomic electron densities overlap even if the static atomic electron densities are described with a non-overlapping partition.

## 5. expHAR test calculations and discussion

Tests of expHAR were performed with ten test systems and using various exponents *n* (1.0, 1.25, 1.5, 2, 3 and 4). In addition to the B3LYP functional, second-order Møller–Plesset perturbation theory (MP2) is used for the electron density calculation (except for BIPa, which is omitted from the MP2 calculations due to its size and the slow convergence of the HAR procedure). For the xylitol structure, HF-based HAR/expHAR refinements were also performed.

### 5.1. *R* factors

**Figure 8:**
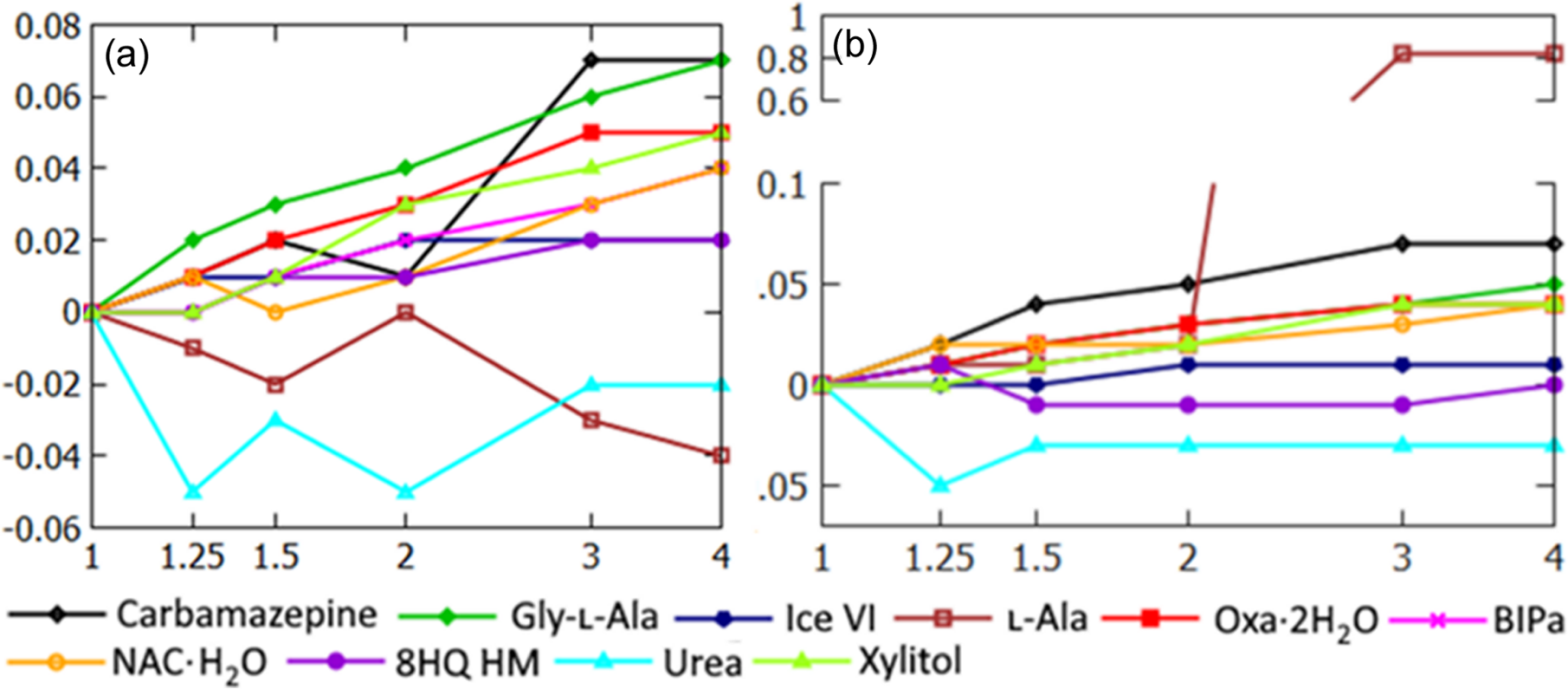
*wR*_2_ (calculated for all data used in the refinement) for expHAR(*n*) as a function of *n* for refinements with (*a*) the B3LYP functional (*b*) using MP2. The large increase of *wR*_2_ for l-Ala MP2 refinement is caused by the change of parameters in the weighting scheme (see the text for details). The lines connecting the datapoints serve as a guide to the eye only and cannot be used to predict *wR*_2_ between the points.

Exponential HAR with *n* > 1 usually leads to a slightly larger *R* factor than HAR. *R*_1_ factors for B3LYP expHAR(2) were up to 0.02% larger than for HAR. A *SHELXL*-type (Sheldrick, 2008 ▸, 2015 ▸) weighting scheme was used during the refinements, *i.e.* the weights are defined as *w* = 1/(σ^2^(*F*_o_^2^) + (*aP*)^2^ + *bP*, where *P* = [2|*F*_c_|^2^ + max(|*F*_o_|^2^,0)], and *F*_c_ and *F*_o_ are the calculated and observed structure factors. Therefore, *R* factors between the refinements are, in principle, not directly comparable since the *a* and *b* parameters may be different for different *n* in expHAR(*n*). For example, for l-alanine the *a* and *b* parameters change from 0.005 and 0 for expHAR(2) to 0.02 and 0.011 for expHAR(3) (MP2 refinements) and *wR*_2_ changes significantly from 3.17 to 3.97%, yet if we used the same weighting scheme as in expHAR(2) in both refinements, then the difference in *wR*_2_ was much smaller, only 0.02%, *wR*_2_ = 3.19% for expHAR(3). For the B3LYP refinements, we did not observe such dramatic changes [Fig. 8 ▸(*a*)]. Changes in *wR*_2_ might be caused by (1) different weighting schemes, (2) different capabilities of the partitions to describe dynamic electron density within convolution approximation and (3) different capabilities of the model to mimic the effects of experimental errors. It is unclear to what extent the factors listed contribute to the differences in *wR*_2_. Though we can eliminate the influence of different weighting schemes, the effects of the two other factors cannot be easily distinguished. Therefore, we will not provide an explanation for the differences in *wR*^2^.

### 5.2. *X*—H bond lengths

**Figure 9:**
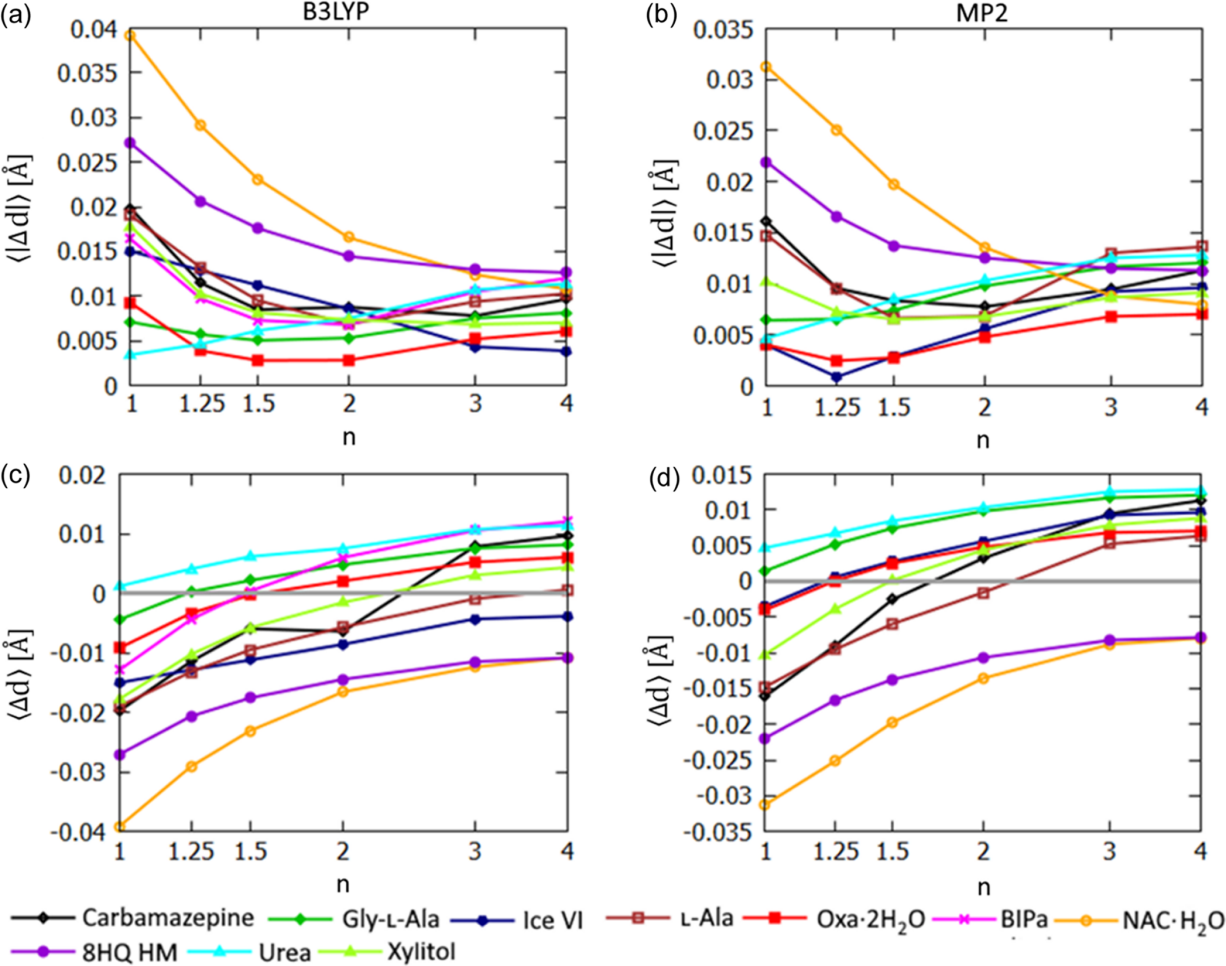
Polar *X*—H bond-length statistics for structures derived with expHAR(*n*) as a function of *n*. Average 〈|Δ*d*|〉 of *X*—H bond lengths from neutron values for refinement with (*a*) B3LYP and (*b*) MP2. Average 〈Δ*d*〉 for *X*—H bond lengths from neutron values for refinement with (*c*) B3LYP and (*d*) MP2.

HAR-related methods provide much more accurate lengths of covalent bonds to hydrogen (*X*—H) than IAM in the case of organic structures. Still, there is a considerable variation in the accuracy of the HAR-derived *X*—H bond lengths [see Fig. 9 ▸(*a*)]. Since C—H bond lengths appear to be reproduced with HAR more accurately than O—H bonds (Woińska *et al.*, 2016 ▸), the bond-length analysis was performed separately for polar *X*—H bonds and for C—H bonds. Statistics calculated without dividing the hydrogen atoms into groups are included in the supporting information.

#### 5.2.1. Polar *X*—H bond lengths

The average absolute difference (〈|Δ*d*|〉) between HAR and neutron-measurement-derived polar *X*—H bond lengths ranges from 3 mÅ for urea to 39 mÅ for NAC·H_2_O for B3LYP refinements (up to 31 mÅ for MP2 refinements). This suggests that there might be room for considerable improvement in some cases. Indeed, for the structures with the largest 〈|Δ*d*|〉 there is a very significant improvement when expHAR is applied [Figs. 9 ▸(*a*) and 9(*b*)] – *e.g.* for NAC·H_2_O 〈|Δ*d*|〉 decreases from 39 to 16 and for 8HQ HM from 27 to 14 mÅ when switching from HAR to expHAR(2) in the case of the B3LYP refinements. The *X*—H bond lengths increased with increasing *n* [Figs. 9 ▸(*c*) and 9 ▸(*d*)]. In the case of the B3LYP-based refinements, bond-length accuracy (measured through 〈|Δ*d*|〉) showed an increased of *n* up to *n* = 2 for all structures except urea. The bond lengths for urea obtained with HAR were already very good and increasing *n* to 2 increased 〈|Δ*d*|〉 from 3 to 8 mÅ in this case. In the case of MP2-based refinements [Fig. 9 ▸(*b*)], the situation is quite similar, however the improvement stops for lower *n* in some cases and, in addition to urea, expHAR does not improve polar *X*—H bond lengths also in the case of Gly-l-Ala. It was observed that MP2 gives systematically longer polar *X*—H bonds than B3LYP (Wieduwilt *et al.*, 2020 ▸; Chodkiewicz *et al.*, 2020 ▸). This is notable in the average difference (〈Δ*d*〉) plots [Figs. 9 ▸(*c*) and 9 ▸(*d*)]. Therefore, a smaller elongation of the polar bonds was necessary to obtain optimal values in the case of MP2 refinements and as a consequence also smaller values of *n* led to optimized values.

Application of HF in HAR leads to relatively long polar *X*—H bond lengths (Chodkiewicz *et al.*, 2020 ▸; Capelli *et al.*, 2014 ▸; Wieduwilt *et al.*, 2020 ▸; Landeros-Rivera *et al.*, 2023 ▸). As a result, the application of HF in expHAR may also lead to long bonds. Such a possibility was tested with the xylitol structure. The O—H bonds in HF-based HAR structures were already too long – on average 9 mÅ longer than from the neutron measurement and for expHAR(2) this number increased to 21 mÅ. HF gave superior O—H bond lengths to B3LYP but larger *R* factors in the case of HAR, whereas in the case of expHAR(2) it was inferior in terms of both *R* factors and bond lengths.

#### 5.2.2. C—H bond lengths

**Figure 10:**
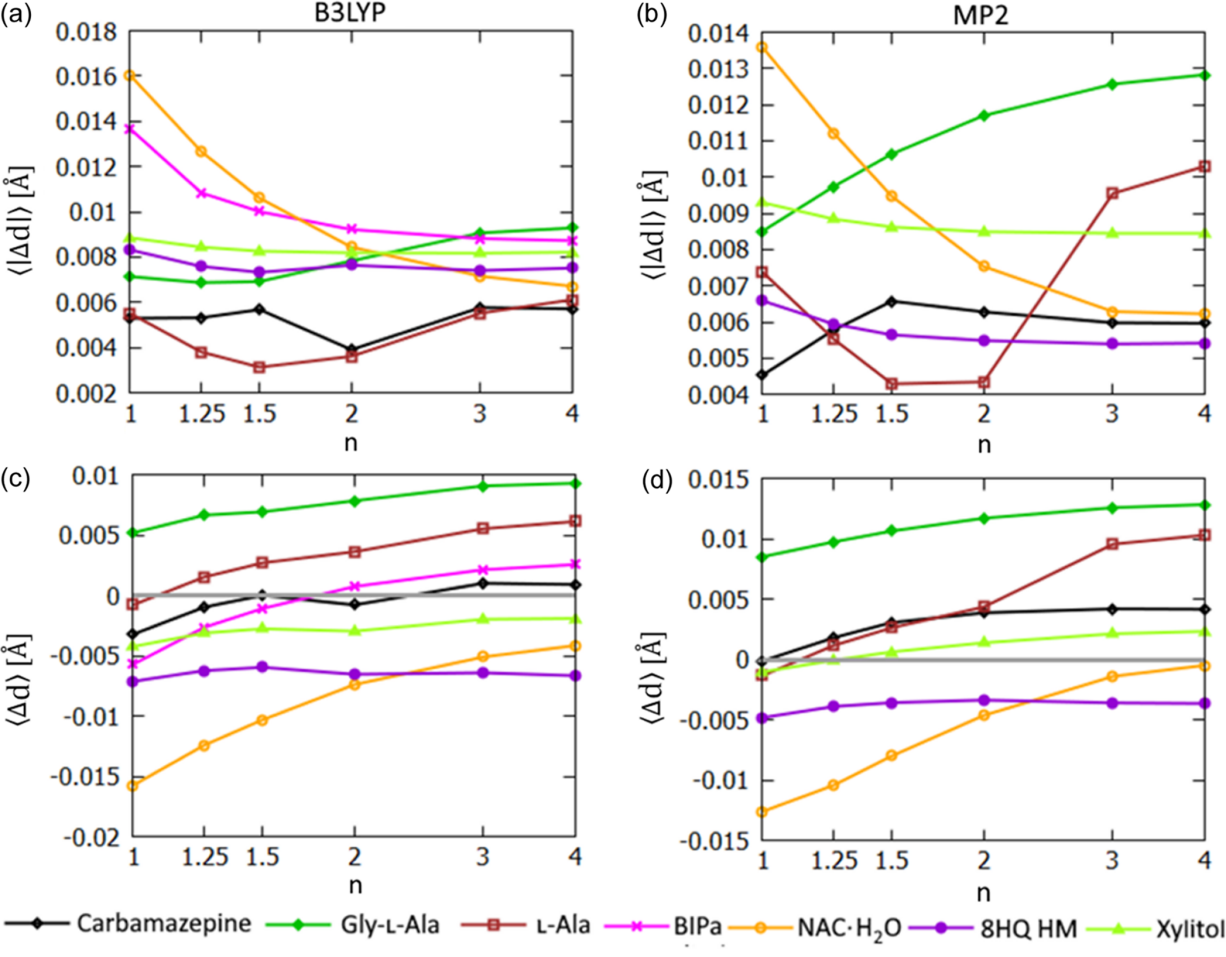
C—H bond-length statistics for structures derived with expHAR(*n*) as a function of *n*. Average 〈|Δ*d*|〉 of *X*—H bond lengths from neutron values for refinement with (*a*) B3LYP and (*b*) MP2. Average 〈Δ*d*〉 of *X*—H bond lengths from neutron values for refinement with (*c*) B3LYP and (*d*) MP2.

Non-polar bond lengths are also improved with exponential HAR in the majority of the tested cases [see Figs. 10 ▸(*a*) and 10 ▸(*b*)]. In this case, the discrepancies between HAR and the reference neutron values are on average smaller than in the case of the polar *X*—H bonds – the maximal 〈|Δ*d*|〉 values were 39 and 16 mÅ for polar and C—H bonds, respectively, for B3LYP refinements and 31 and 14 mÅ for MP2 refinements (NAC·H_2_O structure in all cases). The C—H bond lengths were improved (in terms of 〈|Δ*d*|〉) in 6/7 cases when switching from HAR to expHAR(1.5) for B3LYP-based refinements and in 4/6 cases for MP2-based refinements.

### 5.3. ADPs

**Figure 11:**
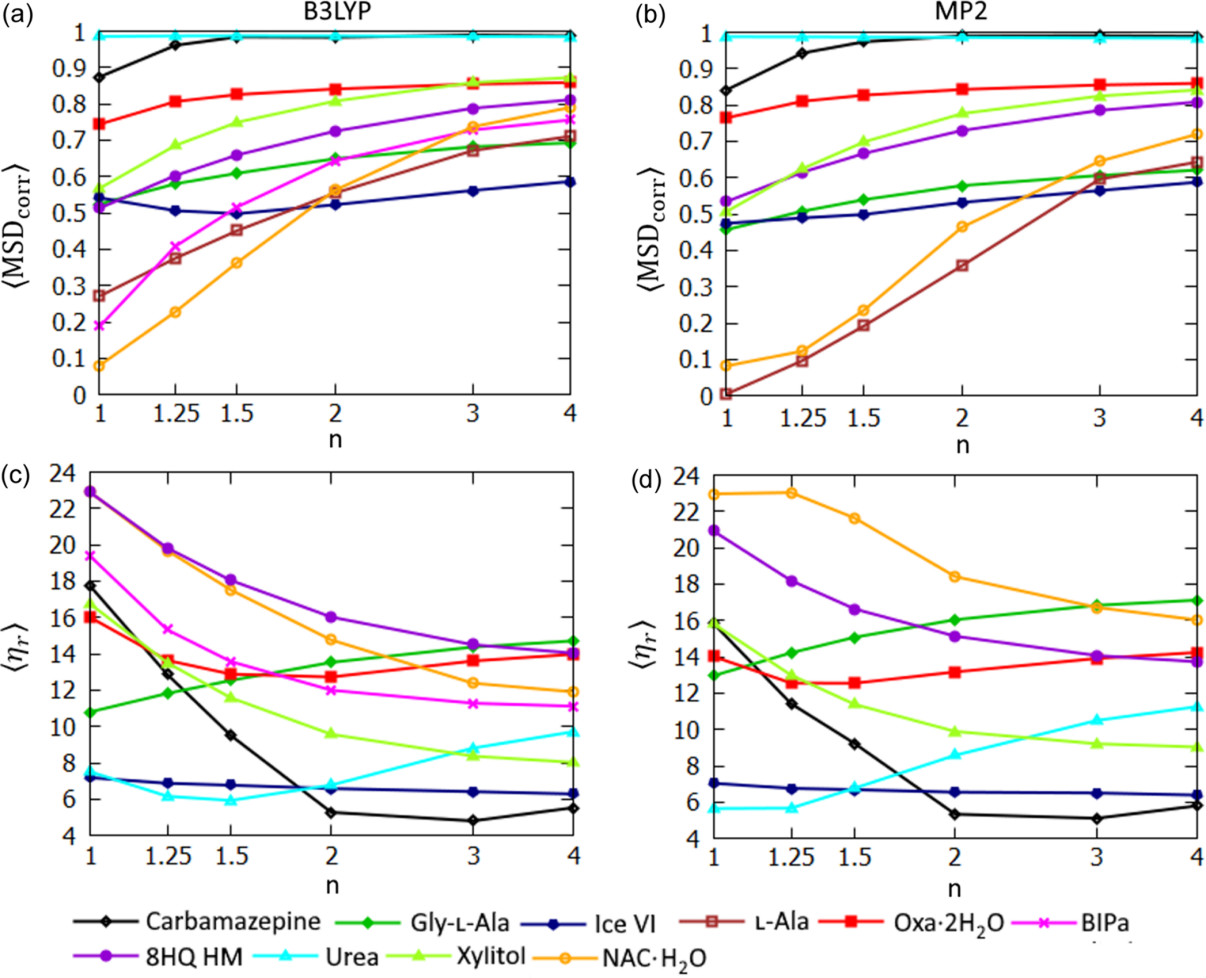
Polar hydrogen ADP statistics for structures derived with expHAR(*n*) as a function of *n*. Average 〈MSD_corr_〉 calculated with the neutron measurement structure as a reference for refinement with (*a*) B3LYP and (*b*) MP2. Average 〈η_*r*_〉 for atomic displacement probability distribution functions, calculated using the neutron measurement structure as a reference for refinement with (*c*) B3LYP and (*d*) MP2.

An application of expHAR(*n*) with *n* > 1 improves the polar hydrogen ADPs in the majority of the test structures (Fig. 11 ▸). It increased the average mean square displacement correlation (〈MSD_corr_〉) in 9/10 structures in the case of B3LYP-based refinements and in 8/9 structures in the case of MP2-based refinements. The average rescaled overlapping coefficient (〈η_*r*_〉) decreased in 8/10 structures (B3LYP) and 6/9 structures (MP2).

**Figure 12:**
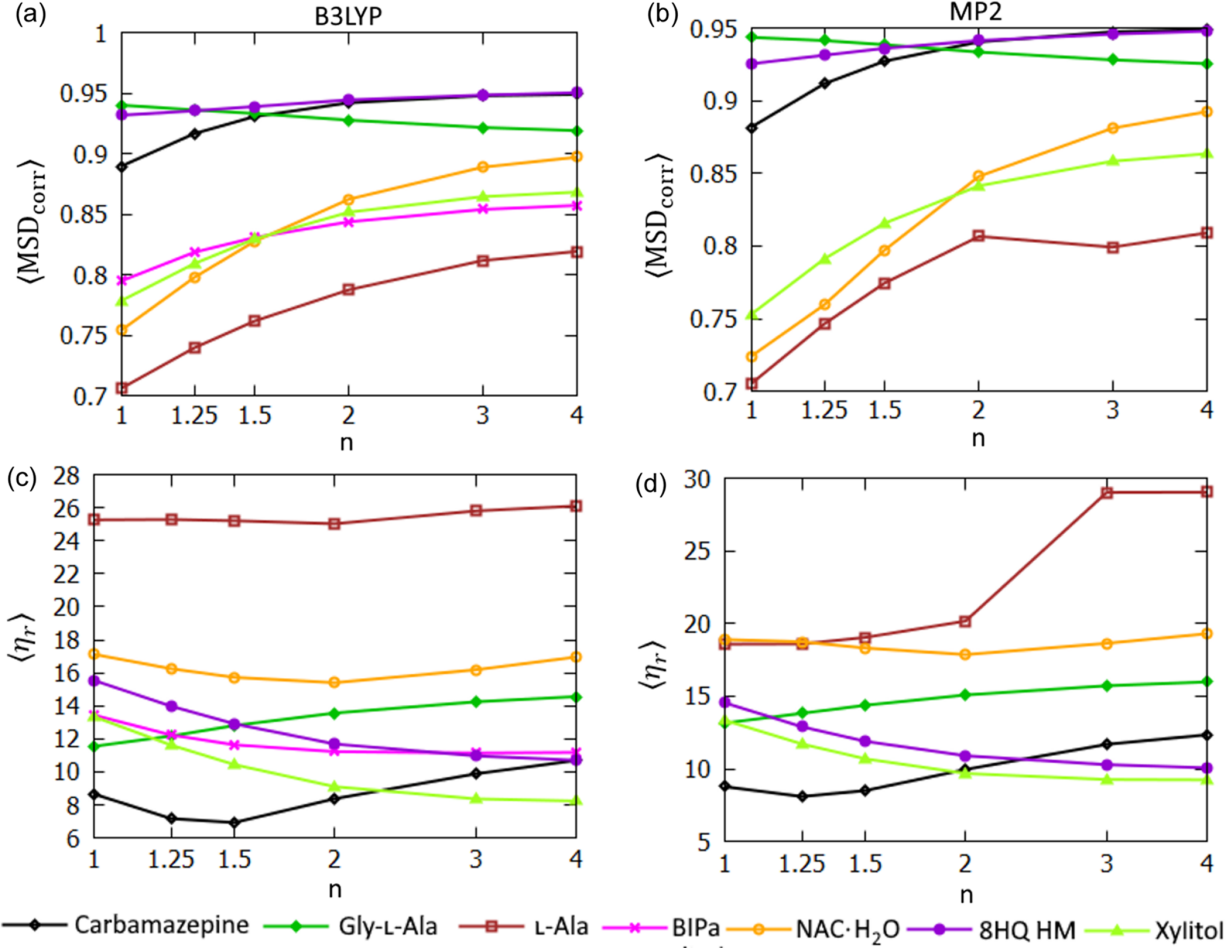
Non-polar hydrogen ADP statistics for structures derived with expHAR(*n*) as a function of *n*. Average 〈MSD_corr_〉 calculated with neutron measurement structure as a reference for refinement with (*a*) B3LYP and (*b*) MP2. Average 〈η_*r*_〉 for atomic displacement probability distribution functions, calculated using the neutron measurement structure as a reference for refinement with (*c*) B3LYP and (*d*) MP2.

In HAR-derived structures, the non-polar hydrogen atoms had higher values of 〈MSD_corr_〉 [Figs. 12 ▸(*a*) and 12 ▸(*b*)] than the polar ones for all structures. Sometimes the difference was very large; for BIPa: 0.79 versus 0.19 (B3LYP refinements). The lowest 〈MSD_corr_〉 for polar hydrogen atoms was 0.08 for NAC·H_2_O while for non-polar ones it was much closer to 1 (*e.g.* 0.71 for l-Ala). Clearly the polar atom ADPs were more challenging to reproduce with HAR. Also the non-polar hydrogen ADPs improved in a majority of the structures (as measured in 〈MSD_corr_〉 terms) when expHAR(*n*) with *n* > 1 was used in all but one structure (glycine) where it decreased slightly. The improvement was less obvious when the 〈η_*r*_〉 indicator was used [Figs. 12 ▸(*c*) and 12 ▸(*d*)], but still 〈η_*r*_〉 values were improved (decreased) in 5/7 cases (B3LYP) and 4/6 cases (MP2) when switching from HAR to expHAR(2)

**Figure 13:**
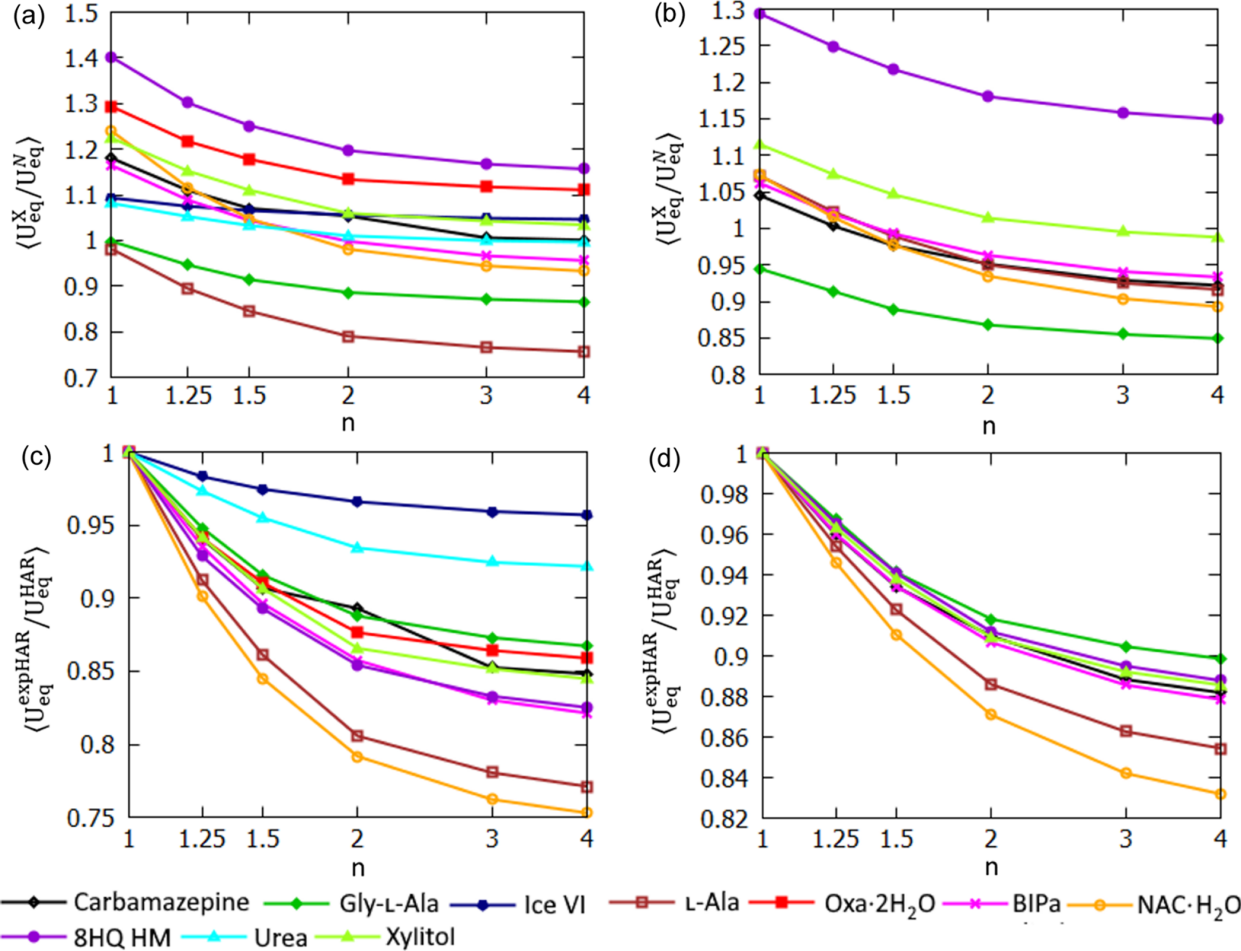
Hydrogen atomic displacement extended for structures derived with expHAR(*n*) as a function of *n*. Average ratio of equivalent isotropic ADPs from expHAR(*n*) and neutron measurement (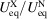) for (*a*) polar and (*b*) non-polar hydrogen atoms. Average ratio of equivalent isotropic ADPs from expHAR(*n*) and HAR (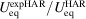) for (*c*) polar and (*d*) non-polar hydrogen atoms.

**Figure 14:**
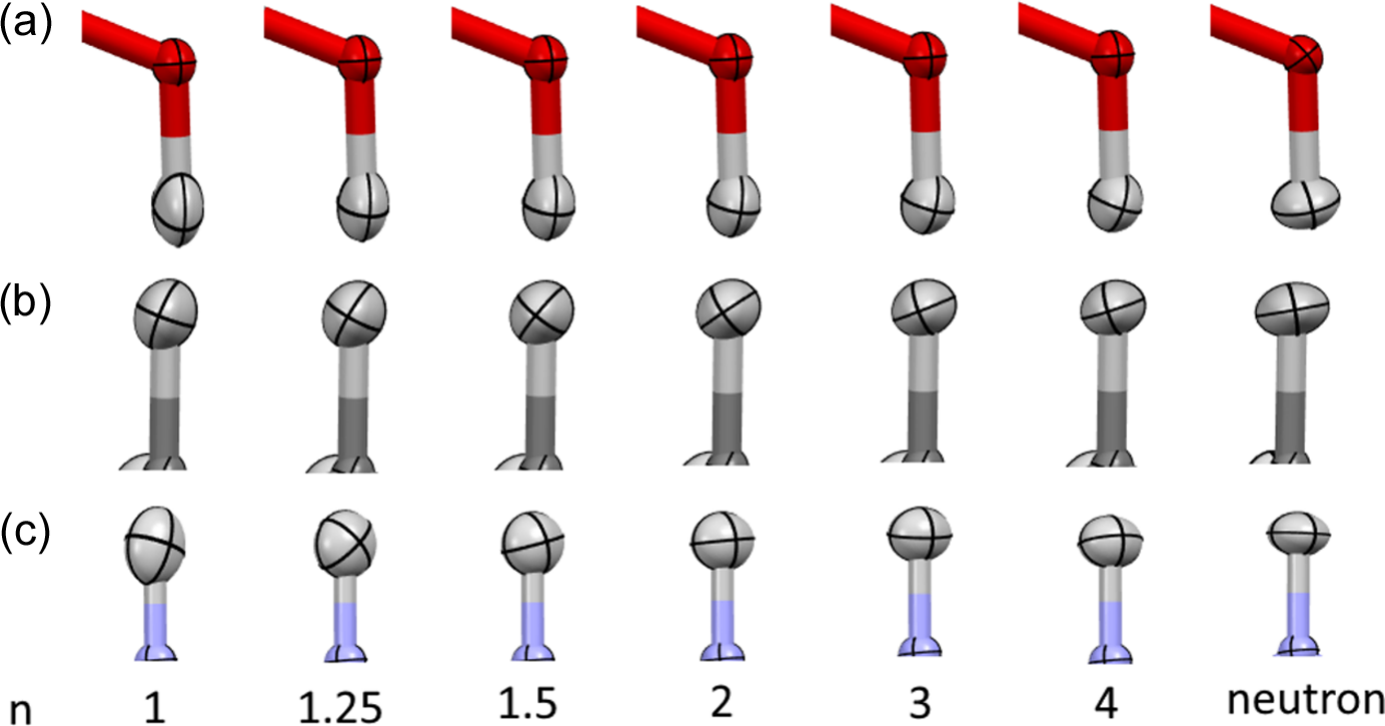
Dependence of thermal ellipsoids on the parameter *n* in expHAR(*n*) for selected hydrogen atoms: (*a*) oxygen-bonded hydrogen atom (H11) in xylitol, (*b*) carbon-bonded hydrogen atom (H5A) in xylitol, (*c*) nitrogen-bonded hydrogen atom (H1m) in BIPa. The last column shows the neutron measurement results.

A clear trend can be observed in the size of the hydrogen ADPs – an increase of *n* in expHAR(*n*) leads to smaller ADPs (smaller *U*_eq_, see Fig. 13 ▸) for both polar and non-polar hydrogen atoms. Increasing the *n* parameter appears to make hydrogen atom thermal ellipsoids ‘thinner’ in the *X*—H bond direction (Fig. 14 ▸), especially when they were too elongated in that direction. Sometimes it led to a very significant improvement in the directionality of the corresponding atomic displacement, *e.g.* the MSD_corr_ for one of the hydrogens shown in Fig. 14 ▸ changed from −0.7 for HAR to 0.66 for expHAR(4). On the other hand, it may also potentially lead to overly small ADPs.

### 5.4. Optimal exponent parameter *n*

Replacing HAR with expHAR(*n*) with *n* > 1 improved the accuracy of hydrogen atom parameters in the majority of the structures tested. There was no improvement in the case of the urea structure – increasing *n* above 1 made the *X*—H bond lengths slightly too large in this case but practically did not change 〈MSD_corr_〉. This might suggest that if switching from HAR to expHAR(*n* > 1) had only a minor impact on hydrogen ADPs (in terms of 〈MSD_corr_〉), then expHAR would not produce significant improvement in hydrogen atom parameters and hence *n* close to 1 was a good choice. For all structures tested, there was practically no deterioration of 〈MSD_corr_〉 observed with an increase of *n*. Therefore, if increasing *n* changes the ADPs, it was probably an improvement of the ADPs, at least in terms of 〈MSD_corr_〉. The situation was different in the case of bond lengths – increasing *n* led to overly long *X*—H bonds. In the case of B3LYP, 〈|Δ*d*|〉 for polar *X*—H bonds decreased with *n* up to *n* = 1.5, except in the urea structure for which it only increased. For *n* > 1.5, 〈|Δ*d*|〉 started to rise in some cases, but for *n* = 2 the increase was below 0.0003 Å. It seems that, provisionally, *n* = 2 can be expected to be a safe choice that improves the accuracy of the refinements or is at least not detrimental in the case of B3LYP refinements. In the case of MP2, *n* = 1.5 is preferential. Note that there is no single choice of parameter *n* that is optimal for all the structures tested and the more general applicability of such rules remains to be tested.

## 6. Conclusions

In this work, we tried to identify factors limiting the accuracy of HAR. Many datasets used so far in testing HAR performance were collected to high resolution. It was observed that cutting the data resolution can sometimes improve the accuracy of HAR. This happened with structures in which non-hydrogen ADPs derived from high-resolution data and those derived from limited-resolution data differed most significantly. On the contrary, cutting the resolution led to lower accuracy when the difference was small. Different sizes of non-hydrogen ADPs from high- and low-resolution refinements suggested that non-hydrogen ADPs refined for high-resolution data were not optimal for low-resolution data. This can be partially compensated for with modifications to the neighbouring hydrogen ADPs, leading to inaccurate hydrogen ADPs. A similar situation was observed when the non-hydrogen ADPs were artificially decreased.

It was assumed that the overlap of atomic electron densities facilitated the described mechanism of distortion of hydrogen ADPs. A new partition of the electron density that can lead to a lower overlap of the atomic electron densities was proposed as a remedy. This ‘exponential Hirshfeld’ partitioning is based on the original Hirshfeld partition and it has an ‘exponent’ parameter *n* allowing for an adjustment of the overlap of atomic electron densities. Setting *n* to 1 makes both partitions identical and increasing *n* reduces the overlap of atomic electron densities.

The effect of applying the exponential Hirshfeld partition in HAR-like procedures (expHAR) was tested on a set of structures of polar organic molecules using electron density calculated with the B3LYP functional (ten structures), and with the MP2 method (nine structures) and a range of *n* parameters. The resulting structures were compared with reference neutron measurements. ExpHAR with *n* > 1 usually led to slightly larger *wR*_2_ agreement factors than HAR (up to 0.02 per cent points for B3LYP refinements); however, comparison of *R* factors is hampered by the fact that the *SHELX*-type weighting scheme is used with adjustable parameters that differ from refinement to refinement, making the *R* factors in principle incomparable (which was especially clear for one of the refinements with the MP2 method). The differences between X-ray- and neutron-derived *X*—H bond lengths were significantly reduced in the case of structures for which they were the largest. The average absolute differences were reduced for 9/10 structures in the case of B3LYP refinements (8/9 for MP2). Hydrogen ADPs also improved in 9/10 of the structures for B3LYP-based refinements and 8/9 for MP2-based refinement when the ADPs were compared with a newly introduced scale-independent similarity measure. The improvement was especially visible in the case of ADPs elongated along the *X*—H bonds.

It was observed that oxygen- and nitro­gen-bonded hydrogen atoms are usually less accurately described with HAR than carbon-bonded ones and that their atomic electron densities overlap to a greater extent with the electron density of their bonding partner. This observation is in line with the assumption that the lower overlap of atomic electron densities makes hydrogen atom parameters less sensitive to the potential inaccuracies of the parameters of their bonding partner. Under this assumption, the lower overlap is not an advantage when there are no such inaccuracies. In such a situation, we would expect that the most accurate structural parameters can be obtained with the electron density partition that most accurately reproduces the dynamic electron density when applied with the convolution approximation for vibrational smearing. In the case of expHAR, reducing the atomic overlap by increasing the parameter *n* leads at some point to too-long *X*—H bonds. Setting *n* to infinity would make the atomic electron densities completely non-overlapping which does not seem to be an optimal choice. To use expHAR most effectively, a method for optimizing the exponent parameter *n* is needed. Yet such a method has not been introduced, only some provisional rules were proposed which appeared to be suitable for the tested systems.

An application of expHAR partition in HAR has led to significant improvements in the description of ADPs of hydrogen atoms in some test cases. We hope that further research will enable even greater accuracy improvements for the HAR-related methods.

## Supplementary Material

Crystallographic information files for all structures. DOI: 10.1107/S2052252524011242/fc5080sup1.zip

Supporting tables, equations and figures. DOI: 10.1107/S2052252524011242/fc5080sup2.pdf

CCDC references: 2407692, 2407693, 2407694, 2407695, 2407696, 2407697, 2407698, 2407699, 2407700, 2407701, 2407702, 2407703, 2407704, 2407705, 2407706, 2407707, 2407708, 2407709, 2407710, 2407711, 2407712, 2407718, 2407719, 2407720, 2407721, 2407722, 2407723, 2407724, 2407725, 2407726, 2407727, 2407728, 2407729, 2407730, 2407731, 2407732, 2407733, 2407734, 2407735, 2407736, 2407737, 2407738, 2407739, 2407740, 2407741, 2407742, 2407743, 2407744, 2407745, 2407746, 2407747, 2407748, 2407749, 2407750, 2407751, 2407752, 2407753, 2407754, 2407755, 2407756, 2407757, 2407758, 2407759, 2407760, 2407761, 2407762, 2407763, 2407764, 2407765, 2407766, 2407774, 2407775, 2407776, 2407777, 2407778, 2407779, 2407780, 2407781, 2407782, 2407783, 2407784, 2407785, 2407786, 2407787, 2407949, 2407950, 2407951, 2407952, 2407953, 2407954, 2407955, 2407956, 2407957, 2407958, 2407959, 2407960, 2407961, 2407962, 2407963, 2407964, 2407965, 2407966, 2407967, 2407968, 2407969, 2407970, 2407971, 2407972, 2407973, 2407974, 2407975, 2407976, 2407977, 2407978, 2407979, 2407980, 2407981, 2407982, 2407983, 2407984, 2407985, 2407986, 2407987, 2407988, 2407989, 2407990, 2407991, 2407992, 2407993, 2408748, 2408749, 2408750, 2408751, 2408752, 2408753, 2408754

## Figures and Tables

**Table 1:** Scale parameter *q* for scaling low-resolution refinement (*d*_min_ = 0.8 Å) non-hydrogen ADPs to values from high-resolution refinement and the average atomic mean square displacement correlation for polar hydrogen atoms for the two refinements calculated with respect to neutron measurement data

		〈MSD_corr_〉	
	*q*	Max *d* = 0.8	Max *d* = *d*_max_
Carbamazepine	0.864	0.982	0.874
NAC·H_2_O	0.884	0.731	0.080
L-Ala	0.924	0.631	0.271
BIPa	0.931	0.416	0.188
Oxa·2H_2_O	0.968	0.685	0.744
8HQ HM	0.990	0.404	0.513
Ice VI	0.993	0.439	0.541
Urea	0.996	0.964	0.986
Xylitol	1.004	0.312	0.568
Gly-L-Ala	1.020	0.278	0.524

**Table 2:** Average overlap coefficients for *X*—H pairs in chosen systems for the Hirshfeld and exponential Hirshfeld partitions

	Hirshfeld partition			Exponential Hirshfeld partition (*n* = 2)		
	O—H	N—H	C—H	O—H	N—H	C—H
Carbamazepine	–	0.0735	0.0510	–	0.0216	0.0186
Gly-L-Ala	–	0.0633	0.050	–	0.0184	0.0183
Xylitol	0.086	–	0.0498	0.0202	–	0.0185
