# Supplementary material for: Towards improved accuracy of Hirshfeld atom refinement with an alternative electron density partition
Source: IUCrJ. 2025 Jan 1;12(Pt 1):74–87. doi: 10.1107/S2052252524011242 (PMC11707693; doi:10.1107/S2052252524011242)

checkCIF/PLATON report

Structure factors have been supplied for datablock(s) 8HQ

THIS REPORT IS FOR GUIDANCE ONLY. IF USED AS PART OF A REVIEW PROCEDURE FOR PUBLICATION, IT SHOULD NOT REPLACE THE EXPERTISE OF AN EXPERIENCED CRYSTALLOGRAPHIC REFEREE.

No syntax errors found. CIF dictionary Interpreting this report

Datablock: 8HQ

Bond precision: C-C = 0.0005 A Wavelength=0.35307

Cell:	a=5.3386(1)	b=9.9878(2)	c=22.3493(4)
	alpha=90	beta=90	gamma=90
Temperature:	15 K		

	Calculated	Reported
Volume	1191.68(4)	1191.68(4)
Space group	P 21 21 21	P 21 21 21
Hall group	P 2ac 2ab	P 2ac 2ab
Moiety formula	C9 H8 N O, C4 H3 O4	C4 H3 O4, C9 H8 N O
Sum formula	C13 H11 N O5	C13 H11 N O5
Mr	261.23	261.24
Dx,g cm-3	1.456	1.456
Z	4	4
Mu (mm-1)	0.038	0.037
F000	544.0	543.9
F000'	543.90	
h,k,lmax	12,23,51	12,22,51
Nref	15707[8672]	12569
Tmin,Tmax	0.996,0.998	
Tmin'	0.996	

Correction method= Not given

Data completeness= 1.45/0.80 Theta(max)= 24.240

R(reflections)= 0.0323(12568) wR2(reflections)= 0.0524(12569)

S = 1.280 Npar= 271

The following ALERTS were generated. Each ALERT has the format
test-name_ALERT_alert-type_alert-level.
Click on the hyperlinks for more details of the test.

Alert level B

```
PLAT934_ALERT_3_B Number of (Iobs-Icalc)/Sigma(W) > 10 Outliers ..          5 Check
               -3-13 -2,  -2-18-19,   0 12  6,   2 18 19,   3 13  2,
```

Alert level C

```
CELLK01_ALERT_1_C  Check that the cell measurement temperature is in Kelvin.
            Value of measurement temperature given =    15.000
PLAT029_ALERT_3_C _diffrn_measured_fraction_theta_full value Low .      0.968 Why?
PLAT042_ALERT_1_C Calc. and Reported MoietyFormula Strings  Differ     Please Check
              Calc: C9 H8 N O, C4 H3 O4
              Rep.: C4 H3 O4, C9 H8 N O
PLAT250_ALERT_2_C Large U3/U1 Ratio for <U(i,j)> Tensor(Resd    1)        2.5 Note
PLAT250_ALERT_2_C Large U3/U1 Ratio for <U(i,j)> Tensor(Resd    2)        2.4 Note
PLAT353_ALERT_3_C Long   N-H  (N0.87,N1.01A)  N1       - H4       .       1.07 Ang.
PLAT911_ALERT_3_C Missing FCF Refl Between Thmin & STh/L=    0.600          41 Report
                6  2  0,   4  4  0,   5  4  0,   0  6  0,   2  7  0,   6  0  1,
                0  6  1,   5  6  2,   0  9  2,   4  3  3,   3  0  5,   6  0  5,
                0  2  5,   4  7  5,   0 10  5,   4  7  6,   0  9  7,   6  0  8,
                0  7  8,   4  0 14,   2  2 14,   0  8 15,   1  0 16,   0  1 16,
                0  4 16,   0  6 16,   0  8 16,   3  0 17,   0  5 20,   0  4 21,
                0  6 21,   3  2 22,   2  0 24,   0  3 24,   2  3 24,   1  4 24,
                0  5 24,   1  0 25,   0  0 26,   1  0 26,   0  2 26,
PLAT915_ALERT_3_C No Flack x Check Done: Low Friedel Pair Coverage         77 %
```

Alert level G

```
ABSMU01_ALERT_1_G  Calculation of _exptl_absorpt_correction_mu
                not performed for this radiation type.
PLAT092_ALERT_4_G Check: Wavelength Given is not Cu,Ga,Mo,Ag,In Ka    0.35307 Ang.
PLAT303_ALERT_2_G Full Occupancy Atom H1        with # Connections       2.00 Check
PLAT778_ALERT_2_G Check O..H..X Bond in CIF: O2        --H1              1.40 Ang.
PLAT881_ALERT_1_G No Datum for _diffrn_reflns_av_R_equivalents ...     Please Do !
PLAT883_ALERT_1_G No Info/Value for _atom_sites_solution_primary .     Please Do !
PLAT912_ALERT_4_G Missing # of FCF Reflections Above STh/L=  0.600       1479 Note
PLAT961_ALERT_5_G Dataset Contains no Negative Intensities .......     Please Check
PLAT969_ALERT_5_G The 'Henn et al.' R-Factor-gap value ..........       2.13 Note
              Predicted wR2: Based on SigI**2  2.46 or SHELX Weight  4.13
PLAT978_ALERT_2_G Number C-C Bonds with Positive Residual Density.          8 Info
PLAT979_ALERT_1_G NoSpherA2 Scattering Factors Used .............     Please Note
```

```
   0 ALERT level A = Most likely a serious problem - resolve or explain
   1 ALERT level B = A potentially serious problem, consider carefully
   8 ALERT level C = Check. Ensure it is not caused by an omission or oversight
  11 ALERT level G = General information/check it is not something unexpected

   6 ALERT type 1 CIF construction/syntax error, inconsistent or missing data
```

```
5 ALERT type 2 Indicator that the structure model may be wrong or deficient
5 ALERT type 3 Indicator that the structure quality may be low
2 ALERT type 4 Improvement, methodology, query or suggestion
2 ALERT type 5 Informative message, check
```

It is advisable to attempt to resolve as many as possible of the alerts in all categories. Often the minor alerts point to easily fixed oversights, errors and omissions in your CIF or refinement strategy, so attention to these fine details can be worthwhile. In order to resolve some of the more serious problems it may be necessary to carry out additional measurements or structure refinements. However, the purpose of your study may justify the reported deviations and the more serious of these should normally be commented upon in the discussion or experimental section of a paper or in the "special_details" fields of the CIF. checkCIF was carefully designed to identify outliers and unusual parameters, but every test has its limitations and alerts that are not important in a particular case may appear. Conversely, the absence of alerts does not guarantee there are no aspects of the results needing attention. It is up to the individual to critically assess their own results and, if necessary, seek expert advice.

Publication of your CIF in IUCr journals

A basic structural check has been run on your CIF. These basic checks will be run on all CIFs submitted for publication in IUCr journals (*Acta Crystallographica*, *Journal of Applied Crystallography*, *Journal of Synchrotron Radiation*); however, if you intend to submit to *Acta Crystallographica Section C* or *E* or *IUCrData*, you should make sure that full publication checks are run on the final version of your CIF prior to submission.

Publication of your CIF in other journals

Please refer to the *Notes for Authors* of the relevant journal for any special instructions relating to CIF submission.

PLATON version of 06/01/2024; check.def file version of 05/01/2024

Datablock 8HQ - ellipsoid plot

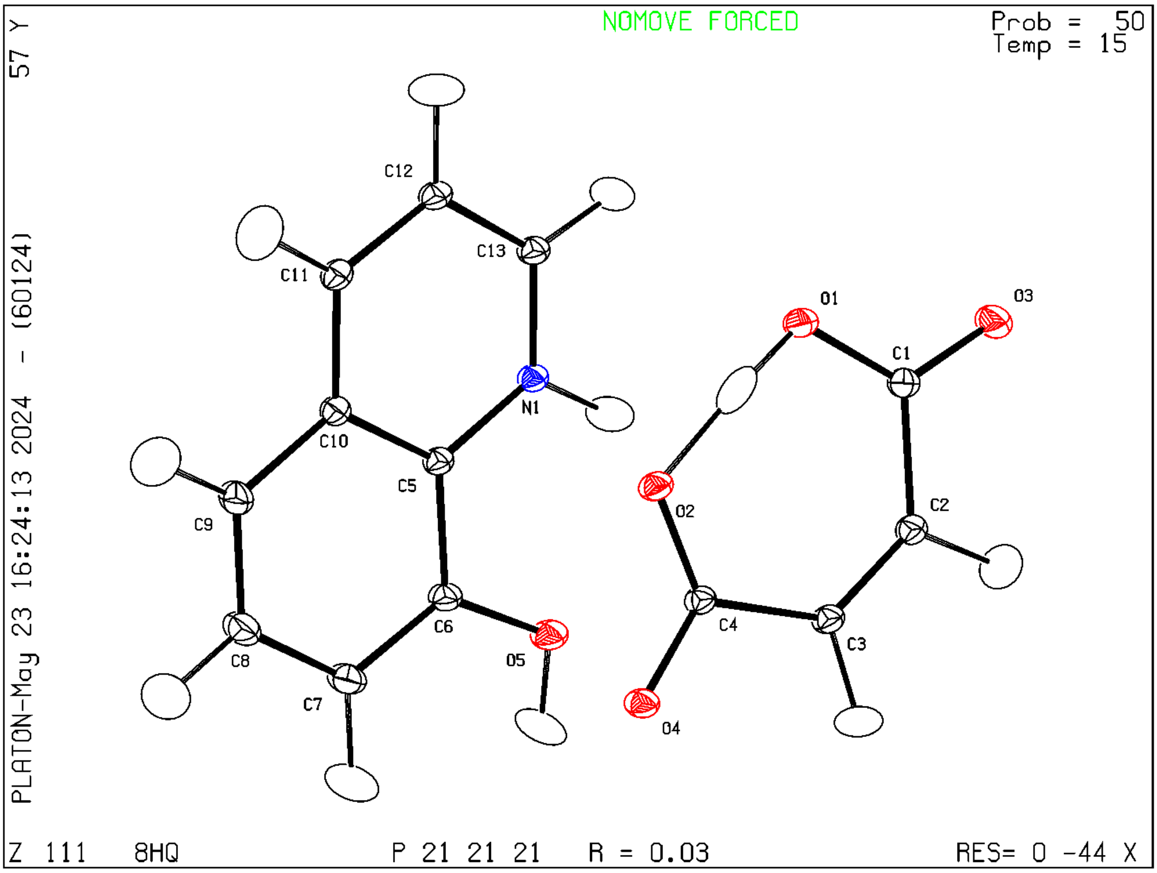

Supplement: Supplementary file 1 [file m-12-00074-sup1.zip › cif_checkcif/8HQ_HM/MP2/1.5_checkcif.pdf]
