# Supplementary material for: Towards improved accuracy of Hirshfeld atom refinement with an alternative electron density partition
Source: IUCrJ. 2025 Jan 1;12(Pt 1):74–87. doi: 10.1107/S2052252524011242 (PMC11707693; doi:10.1107/S2052252524011242)

checkCIF/PLATON report

Structure factors have been supplied for datablock(s) bipa

THIS REPORT IS FOR GUIDANCE ONLY. IF USED AS PART OF A REVIEW PROCEDURE FOR PUBLICATION, IT SHOULD NOT REPLACE THE EXPERTISE OF AN EXPERIENCED CRYSTALLOGRAPHIC REFEREE.

No syntax errors found. CIF dictionary Interpreting this report

Datablock: bipa

Bond precision:	C-C = 0.0005 A		Wavelength=0.71070
Cell:	a=33.5939(5)	b=7.66580(8)	c=25.1324(3)
	alpha=90	beta=114.7162(17)	gamma=90
Temperature:	100 K		

	Calculated	Reported
Volume	5879.28(15)	5879.28(15)
Space group	C 2/c	C 1 2/c 1
Hall group	-C 2yc	-C 2yc
Moiety formula	2(C6 H2 N3 O7), C5 H11 N O2, 2(C3 H5 N2)	C5 H11 N O2, 2(C3 H5 N2), 2(C6 H2 N3 O7)
Sum formula	C23 H25 N11 O16	C23 H25 N11 O16
Mr	711.54	711.52
Dx,g cm-3	1.608	1.608
Z	8	8
Mu (mm-1)	0.138	0.138
F000	2944.0	2946.0
F000’	2945.79	
h,k,lmax	80,18,60	77,18,59
Nref	42407	31489
Tmin,Tmax	0.966,0.978	
Tmin’	0.961	

Correction method= Not given

Data completeness= 0.743 Theta(max)= 58.410

R(reflections)= 0.0368(23190) wR2(reflections)= 0.0925(31489)

S = 0.907 Npar= 676

The following ALERTS were generated. Each ALERT has the format
test-name_ALERT_alert-type_alert-level.
Click on the hyperlinks for more details of the test.

Alert level B

```
PLAT934_ALERT_3_B Number of (Iobs-Icalc)/Sigma(W) > 10 Outliers ..          5 Check
                0   6 12,    3   3   0,   12   6   0,   15   3   3,   18   0   6,
```

Alert level C

```
DIFMX02_ALERT_1_C  The maximum difference density is > 0.1*ZMAX*0.75
            The relevant atom site should be identified.
PLAT029_ALERT_3_C _diffrn_measured_fraction_theta_full value Low .      0.965 Why?
PLAT042_ALERT_1_C Calc. and Reported MoietyFormula Strings  Differ     Please Check
              Calc: 2(C6 H2 N3 O7), C5 H11 N O2, 2(C3 H5 N2)
              Rep.: C5 H11 N O2, 2(C3 H5 N2), 2(C6 H2 N3 O7)
PLAT097_ALERT_2_C Large Reported Max.  (Positive) Residual Density       0.71 eA-3
PLAT351_ALERT_3_C Long   C-H (X0.96,N1.08A)  C15      - H1G       .       1.11 Ang.
PLAT353_ALERT_3_C Long   N-H (N0.87,N1.01A)  N1A      - H1L       .       1.03 Ang.
PLAT353_ALERT_3_C Long   N-H (N0.87,N1.01A)  N3A      - H3A       .       1.04 Ang.
PLAT353_ALERT_3_C Long   N-H (N0.87,N1.01A)  N1B      - H1M       .       1.02 Ang.
PLAT353_ALERT_3_C Long   N-H (N0.87,N1.01A)  N3B      - H3B       .       1.03 Ang.
PLAT430_ALERT_2_C Short Inter D...A Contact  O9       ..O9        .       2.90 Ang.
                                                1-x,y,1/2-z   =      2_655 Check
PLAT906_ALERT_3_C Large K Value in the Analysis of Variance ......      2.937 Check
PLAT910_ALERT_3_C Missing # of FCF Reflection(s) Below Theta(Min).          5 Note
                2   0   0,   -4   0   2,   -2   0   2,    0   0   2,    2   0   2,
PLAT911_ALERT_3_C Missing FCF Refl Between Thmin & STh/L=    0.600        183 Report
                8   0   0,   36   0   0, -36   0   2, -24   0   2, -12   0   2, -10   0   2,
               -8   0   2,   -6   0   2,   -1   1   2, -38   0   4, -20   0   4, -18   0   4,
              -16   0   4, -14   0   4, -12   0   4, -10   0   4,  -8   0   4,  -6   0   4,
               -2   0   4,    0   0   4,    2   0   4,    4   0   4,  34   0   4, -38   0   6,
              -36   0   6, -26   0   6, -24   0   6, -22   0   6, -20   0   6, -18   0   6,
              -14   0   6, -12   0   6, -10   0   6,  -8   0   6,  -6   0   6,  -4   0   6,
               -2   0   6,    0   0   6,    2   0   6,    4   0   6,   6   0   6,  10   0   6,
              -38   0   8, -28   0   8, -26   0   8, -24   0   8, -22   0   8, -18   0   8,
              -16   0   8, -14   0   8, -12   0   8, -10   0   8,  -8   0   8,  -6   0   8,
               -4   0   8,   -2   0   8,    0   0   8,    2   0   8,   4   0   8,   6   0   8,
                8   0   8,   10   0   8,   12   0   8, -39   1   9, -40   0  10, -38   0  10,
              -28   0  10, -26   0  10, -22   0  10, -20   0  10, -18   0  10, -16   0  10,
              -14   0  10, -12   0  10, -10   0  10,  -8   0  10,  -6   0  10,  -4   0  10,
               -2   0  10,    0   0  10,    2   0  10,   4   0  10,   6   0  10,   8   0  10,
               10   0  10,  -40   0  12, -32   0  12, -24   0  12, -22   0  12, -20   0  12,
              -18   0  12,  -16   0  12, -14   0  12, -12   0  12, -10   0  12,  -8   0  12,
```

Alert level G

```
PLAT068_ALERT_1_G Reported F000 Differs from Calcd (or Missing)...     Please Check
PLAT128_ALERT_4_G Alternate Setting for Input Space Group     C2/c        I2/a Note
PLAT432_ALERT_2_G Short Inter X...Y Contact  O1A      ..C7B       .       2.97 Ang.
                                          1/2-x,1/2-y,-z   =      7_555 Check
PLAT432_ALERT_2_G Short Inter X...Y Contact  O5A      ..C14       .       2.92 Ang.
                                     -1/2+x,1/2-y,-1/2+z   =      8_455 Check
PLAT432_ALERT_2_G Short Inter X...Y Contact  C2B      ..C10       .       3.18 Ang.
```

```
                                          1/2-x,1/2-y,-z  =      7_555 Check
PLAT790_ALERT_4_G Centre of Gravity not Within Unit Cell: Resd.  #          3 Note
              C5 H11 N O2
PLAT881_ALERT_1_G No Datum for _diffrn_reflns_av_R_equivalents ...     Please Do !
PLAT883_ALERT_1_G No Info/Value for _atom_sites_solution_primary .     Please Do !
PLAT912_ALERT_4_G Missing # of FCF Reflections Above STh/L=  0.600      10624 Note
PLAT956_ALERT_1_G Calculated (ThMax) and Actual (FCF) Hmax Differ          3 Units
PLAT960_ALERT_3_G Number of Intensities with I  <   - 2*sig(I) ...         8 Check
PLAT963_ALERT_2_G Both SHELXL WEIGHT Parameter Values Zero .......     Please Check
PLAT969_ALERT_5_G The 'Henn et al.' R-Factor-gap value ...........       0.90 Note
              Predicted wR2: Based on SigI**2 10.32 or SHELX Weight 10.32
PLAT978_ALERT_2_G Number C-C Bonds with Positive Residual Density.         10 Info
PLAT979_ALERT_1_G NoSpherA2 Scattering Factors Used .............     Please Note
```

---

0 **ALERT level A** = Most likely a serious problem - resolve or explain
1 **ALERT level B** = A potentially serious problem, consider carefully
13 **ALERT level C** = Check. Ensure it is not caused by an omission or oversight
15 **ALERT level G** = General information/check it is not something unexpected

7 ALERT type 1 CIF construction/syntax error, inconsistent or missing data
7 ALERT type 2 Indicator that the structure model may be wrong or deficient
11 ALERT type 3 Indicator that the structure quality may be low
3 ALERT type 4 Improvement, methodology, query or suggestion
1 ALERT type 5 Informative message, check

It is advisable to attempt to resolve as many as possible of the alerts in all categories. Often the minor alerts point to easily fixed oversights, errors and omissions in your CIF or refinement strategy, so attention to these fine details can be worthwhile. In order to resolve some of the more serious problems it may be necessary to carry out additional measurements or structure refinements. However, the purpose of your study may justify the reported deviations and the more serious of these should normally be commented upon in the discussion or experimental section of a paper or in the "special_details" fields of the CIF. checkCIF was carefully designed to identify outliers and unusual parameters, but every test has its limitations and alerts that are not important in a particular case may appear. Conversely, the absence of alerts does not guarantee there are no aspects of the results needing attention. It is up to the individual to critically assess their own results and, if necessary, seek expert advice.

Publication of your CIF in IUCr journals

A basic structural check has been run on your CIF. These basic checks will be run on all CIFs submitted for publication in IUCr journals (*Acta Crystallographica*, *Journal of Applied Crystallography*, *Journal of Synchrotron Radiation*); however, if you intend to submit to *Acta Crystallographica Section C* or *E* or *IUCrData*, you should make sure that full publication checks are run on the final version of your CIF prior to submission.

Publication of your CIF in other journals

Please refer to the *Notes for Authors* of the relevant journal for any special instructions relating to CIF submission.

PLATON version of 06/01/2024; check.def file version of 05/01/2024

Datablock bipa - ellipsoid plot

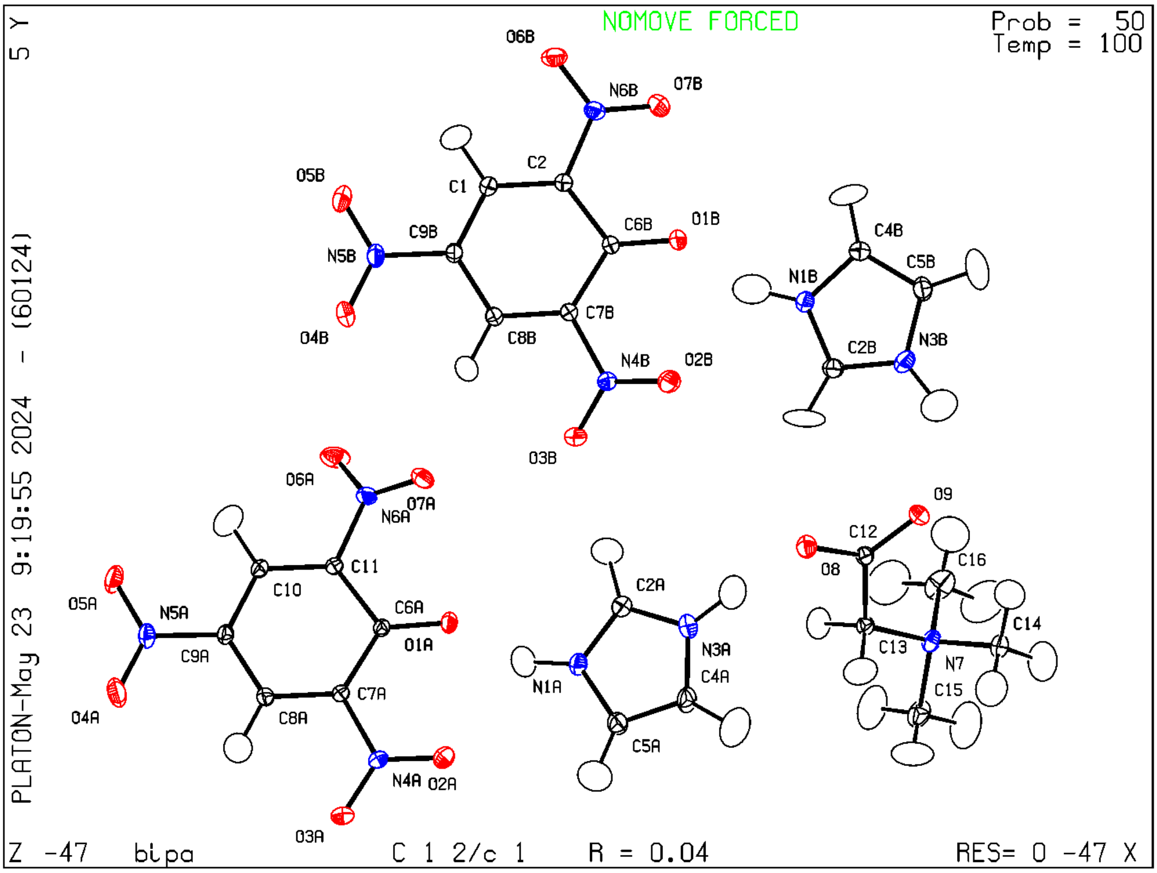

Supplement: Supplementary file 1 [file m-12-00074-sup1.zip › cif_checkcif/BIPa/B3LYP/1_checkcif.pdf]
