# Supplementary material for: Towards improved accuracy of Hirshfeld atom refinement with an alternative electron density partition
Source: IUCrJ. 2025 Jan 1;12(Pt 1):74–87. doi: 10.1107/S2052252524011242 (PMC11707693; doi:10.1107/S2052252524011242)

checkCIF/PLATON report

Structure factors have been supplied for datablock(s) formIII_XRAY_xd

THIS REPORT IS FOR GUIDANCE ONLY. IF USED AS PART OF A REVIEW PROCEDURE FOR PUBLICATION, IT SHOULD NOT REPLACE THE EXPERTISE OF AN EXPERIENCED CRYSTALLOGRAPHIC REFEREE.

No syntax errors found. CIF dictionary Interpreting this report

Datablock: formIII_XRAY_xd

Bond precision:	C-C = 0.0003 A		Wavelength=0.71073
Cell:	a=7.4893(4)	b=11.0323(5)	c=13.7640(6)
	alpha=90	beta=92.953(2)	gamma=90
Temperature:	100 K		

	Calculated	Reported
Volume	1135.73(9)	1135.73(9)
Space group	P 21/n	P 1 21/n 1
Hall group	-P 2yn	-P 2ybc (x-
Moiety formula	C15 H12 N2 O	C15 H12 N2 O
Sum formula	C15 H12 N2 O	C15 H12 N2 O
Mr	236.27	236.27
Dx,g cm-3	1.382	1.382
Z	4	4
Mu (mm-1)	0.089	0.089
F000	496.0	496.2
F000'	496.19	
h,k,lmax	17,26,32	17,26,32
Nref	16157	16012
Tmin,Tmax	0.963,0.988	
Tmin'	0.951	

Correction method= Not given

Data completeness= 0.991 Theta(max)= 57.990

R(reflections)= 0.0264(12827) wR2(reflections)= 0.0661(16012)

S = 1.155 Npar= 271

The following ALERTS were generated. Each ALERT has the format
test-name_ALERT_alert-type_alert-level.
Click on the hyperlinks for more details of the test.

Alert level C

```
PLAT126_ALERT_1_C Error in or Uninterpretable Hall Symbol  ....... -P 2YBC (X Check
PLAT353_ALERT_3_C Long   N-H (N0.87,N1.01A)  N1       - H1A      .       1.01 Ang.
PLAT353_ALERT_3_C Long   N-H (N0.87,N1.01A)  N1       - H1B      .       1.03 Ang.
PLAT420_ALERT_2_C D-H Bond Without Acceptor  N1       --H1A      .     Please Check
PLAT905_ALERT_3_C Negative K value in the Analysis of Variance ...     -1.850 Report
PLAT934_ALERT_3_C Number of (Iobs-Icalc)/Sigma(W) > 10 Outliers ..          1 Check
                1 19  2,
```

Alert level G

```
PLAT881_ALERT_1_G No Datum for _diffrn_reflns_av_R_equivalents ...     Please Do !
PLAT883_ALERT_1_G No Info/Value for _atom_sites_solution_primary .     Please Do !
PLAT912_ALERT_4_G Missing # of FCF Reflections Above STh/L=  0.600        145 Note
PLAT969_ALERT_5_G The 'Henn et al.' R-Factor-gap value ..........        1.49 Note
              Predicted wR2: Based on SigI**2  4.45 or SHELX Weight  5.77
PLAT978_ALERT_2_G Number C-C Bonds with Positive Residual Density.         11 Info
PLAT979_ALERT_1_G NoSpherA2 Scattering Factors Used .............     Please Note
PLAT992_ALERT_5_G Repd & Actual _reflns_number_gt Values Differ by          2 Check
```

0 **ALERT level A** = Most likely a serious problem - resolve or explain
0 **ALERT level B** = A potentially serious problem, consider carefully
6 **ALERT level C** = Check. Ensure it is not caused by an omission or oversight
7 **ALERT level G** = General information/check it is not something unexpected

4 ALERT type 1 CIF construction/syntax error, inconsistent or missing data
2 ALERT type 2 Indicator that the structure model may be wrong or deficient
4 ALERT type 3 Indicator that the structure quality may be low
1 ALERT type 4 Improvement, methodology, query or suggestion
2 ALERT type 5 Informative message, check

It is advisable to attempt to resolve as many as possible of the alerts in all categories. Often the minor alerts point to easily fixed oversights, errors and omissions in your CIF or refinement strategy, so attention to these fine details can be worthwhile. In order to resolve some of the more serious problems it may be necessary to carry out additional measurements or structure refinements. However, the purpose of your study may justify the reported deviations and the more serious of these should normally be commented upon in the discussion or experimental section of a paper or in the "special_details" fields of the CIF. checkCIF was carefully designed to identify outliers and unusual parameters, but every test has its limitations and alerts that are not important in a particular case may appear. Conversely, the absence of alerts does not guarantee there are no aspects of the results needing attention. It is up to the individual to critically assess their own results and, if necessary, seek expert advice.

Publication of your CIF in IUCr journals

A basic structural check has been run on your CIF. These basic checks will be run on all CIFs submitted for publication in IUCr journals (*Acta Crystallographica*, *Journal of Applied Crystallography*, *Journal of Synchrotron Radiation*); however, if you intend to submit to *Acta Crystallographica Section C* or *E* or *IUCrData*, you should make sure that full publication checks are run on the final version of your CIF prior to submission.

Publication of your CIF in other journals

Please refer to the *Notes for Authors* of the relevant journal for any special instructions relating to CIF submission.

PLATON version of 06/01/2024; check.def file version of 05/01/2024

Datablock formIII_XRAY_xd - ellipsoid plot

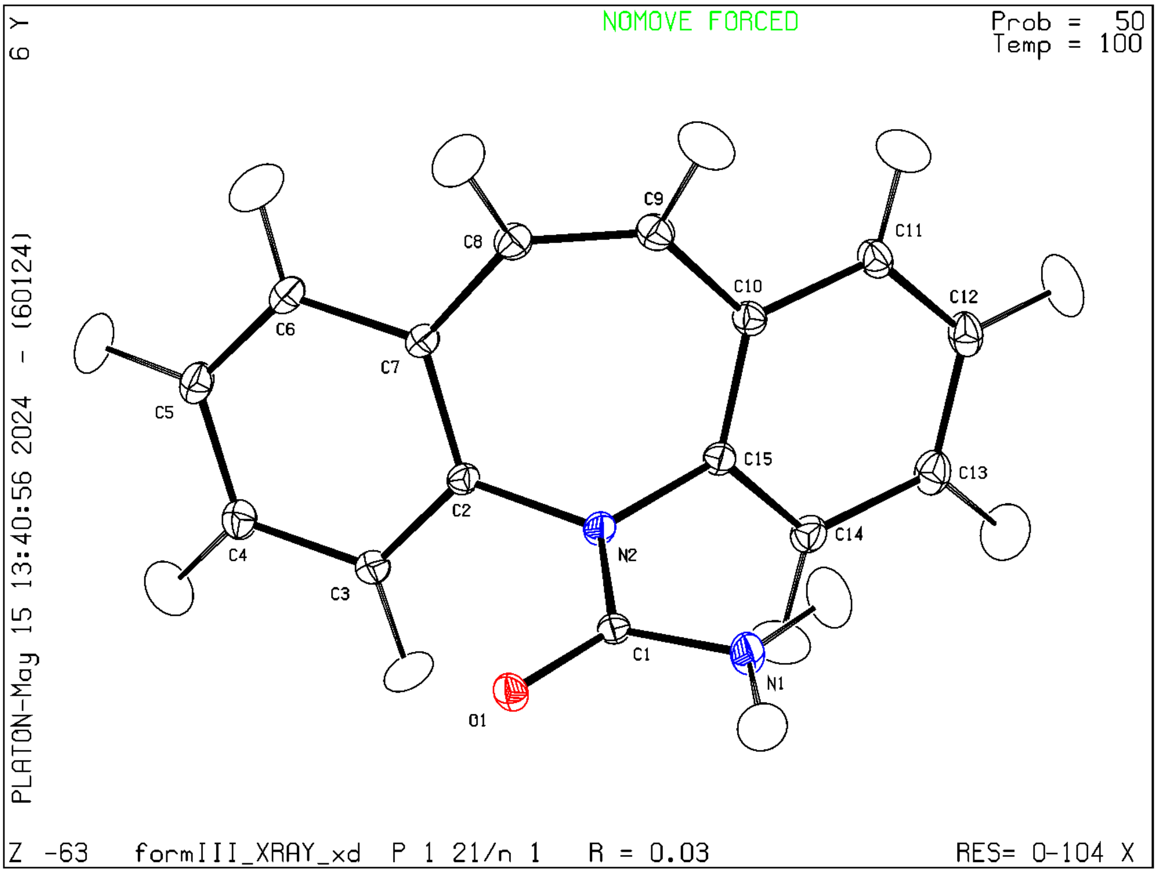

Supplement: Supplementary file 1 [file m-12-00074-sup1.zip › cif_checkcif/carbamazepine/B3LYP/4_checkcif.pdf]
