# Supplementary material for: Towards improved accuracy of Hirshfeld atom refinement with an alternative electron density partition
Source: IUCrJ. 2025 Jan 1;12(Pt 1):74–87. doi: 10.1107/S2052252524011242 (PMC11707693; doi:10.1107/S2052252524011242)

checkCIF/PLATON report

Structure factors have been supplied for datablock(s) gly_ala

THIS REPORT IS FOR GUIDANCE ONLY. IF USED AS PART OF A REVIEW PROCEDURE FOR PUBLICATION, IT SHOULD NOT REPLACE THE EXPERTISE OF AN EXPERIENCED CRYSTALLOGRAPHIC REFEREE.

No syntax errors found.   CIF dictionary   Interpreting this report

Datablock: gly_ala

```
Bond precision:   C-C = 0.0007 A          Wavelength=0.52590

Cell:                a=7.487(2)       b=9.4960(1)       c=9.7099(4)
                     alpha=90         beta=90           gamma=90
Temperature:         150 K

                 Calculated                    Reported
Volume           690.34(19)                    690.34(19)
Space group      P 21 21 21                    P 21 21 21
Hall group       P 2ac 2ab                     P 2ac 2ab
Moiety formula   C5 H10 N2 O3                  C5 H10 N2 O3
Sum formula      C5 H10 N2 O3                  C5 H10 N2 O3
Mr               146.15                        146.15
Dx,g cm-3        1.406                         1.406
Z                4                             4
Mu (mm-1)        0.063                         0.063
F000             312.0                         312.0
F000'            312.03
h,k,lmax         9,11,12                       9,11,12
Nref             1415[ 844]                    1404
Tmin,Tmax        0.994,0.997
Tmin'            0.994

Correction method= Not given

Data completeness= 1.66/0.99        Theta(max)= 19.200

                                                          wR2(reflections)=
R(reflections)= 0.0108( 1379)                             0.0241( 1404)

S = 1.040                 Npar= 181
```

The following ALERTS were generated. Each ALERT has the format
test-name_ALERT_alert-type_alert-level.
Click on the hyperlinks for more details of the test.

Alert level B

```
PLAT089_ALERT_3_B Poor Data / Parameter Ratio (Zmax < 18) ........       4.62 Note
```

Alert level C

```
STRVA01_ALERT_4_C           Flack parameter is too small
           From the CIF: _refine_ls_abs_structure_Flack   -1.000
           From the CIF: _refine_ls_abs_structure_Flack_su    2.000
PLAT351_ALERT_3_C Long   C-H (X0.96,N1.08A)  C2       - H2       .       1.11 Ang.
PLAT353_ALERT_3_C Long   N-H (N0.87,N1.01A)  N1       - H1N1     .       1.02 Ang.
PLAT353_ALERT_3_C Long   N-H (N0.87,N1.01A)  N2       - H1N2     .       1.05 Ang.
PLAT353_ALERT_3_C Long   N-H (N0.87,N1.01A)  N2       - H2N2     .       1.04 Ang.
PLAT353_ALERT_3_C Long   N-H (N0.87,N1.01A)  N2       - H3N2     .       1.04 Ang.
PLAT911_ALERT_3_C Missing FCF Refl Between Thmin & STh/L=    0.600          8 Report
                 2  1  0,   0  2  0,   3  2  0,   2  3  0,   1  0  1,   0  2  1,
                 0  0  2,   1  2  2,
PLAT934_ALERT_3_C Number of (Iobs-Icalc)/Sigma(W) > 10 Outliers ..          1 Check
                 1  1  2,
```

Alert level G

```
ABSMU01_ALERT_1_G  Calculation of _exptl_absorpt_correction_mu
                 not performed for this radiation type.
PLAT032_ALERT_4_G Std. Uncertainty on Flack Parameter Value High .      2.000 Report
PLAT720_ALERT_4_G Number of Unusual/Non-Standard Labels .........          4 Note
               H1N1    H1N2    H2N2    H3N2
PLAT791_ALERT_4_G Model has Chirality at C2          (Sohncke SpGr)          S Verify
PLAT883_ALERT_1_G No Info/Value for _atom_sites_solution_primary .     Please Do !
PLAT913_ALERT_3_G Missing # of Very Strong Reflections in FCF ....          2 Note
                 2  1  0,   1  2  2,
PLAT916_ALERT_2_G Hooft y and Flack x Parameter Values Differ by .       0.80 Check
PLAT967_ALERT_5_G Note: Two-Theta Cutoff Value in Embedded .res ..       38.4 Degree
PLAT969_ALERT_5_G The 'Henn et al.' R-Factor-gap value ..........      1.572 Note
               Predicted wR2: Based on SigI**2  1.53 or SHELX Weight  2.31
PLAT978_ALERT_2_G Number C-C Bonds with Positive Residual Density.          2 Info
PLAT979_ALERT_1_G NoSpherA2 Scattering Factors Used .............     Please Note
```

0 **ALERT level A** = Most likely a serious problem - resolve or explain
1 **ALERT level B** = A potentially serious problem, consider carefully
8 **ALERT level C** = Check. Ensure it is not caused by an omission or oversight
11 **ALERT level G** = General information/check it is not something unexpected

3 ALERT type 1 CIF construction/syntax error, inconsistent or missing data
2 ALERT type 2 Indicator that the structure model may be wrong or deficient
9 ALERT type 3 Indicator that the structure quality may be low
4 ALERT type 4 Improvement, methodology, query or suggestion
2 ALERT type 5 Informative message, check

Validation response form

Please find below a validation response form (VRF) that can be filled in and pasted into your CIF.

```
# start Validation Reply Form
_vrf_PLAT089_gly_ala
;
PROBLEM: Poor Data / Parameter Ratio (Zmax < 18) ........       4.62 Note
RESPONSE: ...
;
# end Validation Reply Form
```

It is advisable to attempt to resolve as many as possible of the alerts in all categories. Often the minor alerts point to easily fixed oversights, errors and omissions in your CIF or refinement strategy, so attention to these fine details can be worthwhile. In order to resolve some of the more serious problems it may be necessary to carry out additional measurements or structure refinements. However, the purpose of your study may justify the reported deviations and the more serious of these should normally be commented upon in the discussion or experimental section of a paper or in the "special_details" fields of the CIF. checkCIF was carefully designed to identify outliers and unusual parameters, but every test has its limitations and alerts that are not important in a particular case may appear. Conversely, the absence of alerts does not guarantee there are no aspects of the results needing attention. It is up to the individual to critically assess their own results and, if necessary, seek expert advice.

Publication of your CIF in IUCr journals

A basic structural check has been run on your CIF. These basic checks will be run on all CIFs submitted for publication in IUCr journals (*Acta Crystallographica*, *Journal of Applied Crystallography*, *Journal of Synchrotron Radiation*); however, if you intend to submit to *Acta Crystallographica Section C* or *E* or *IUCrData*, you should make sure that full publication checks are run on the final version of your CIF prior to submission.

Publication of your CIF in other journals

Please refer to the *Notes for Authors* of the relevant journal for any special instructions relating to CIF submission.

PLATON version of 22/08/2024; check.def file version of 21/08/2024

Datablock gly_ala - ellipsoid plot

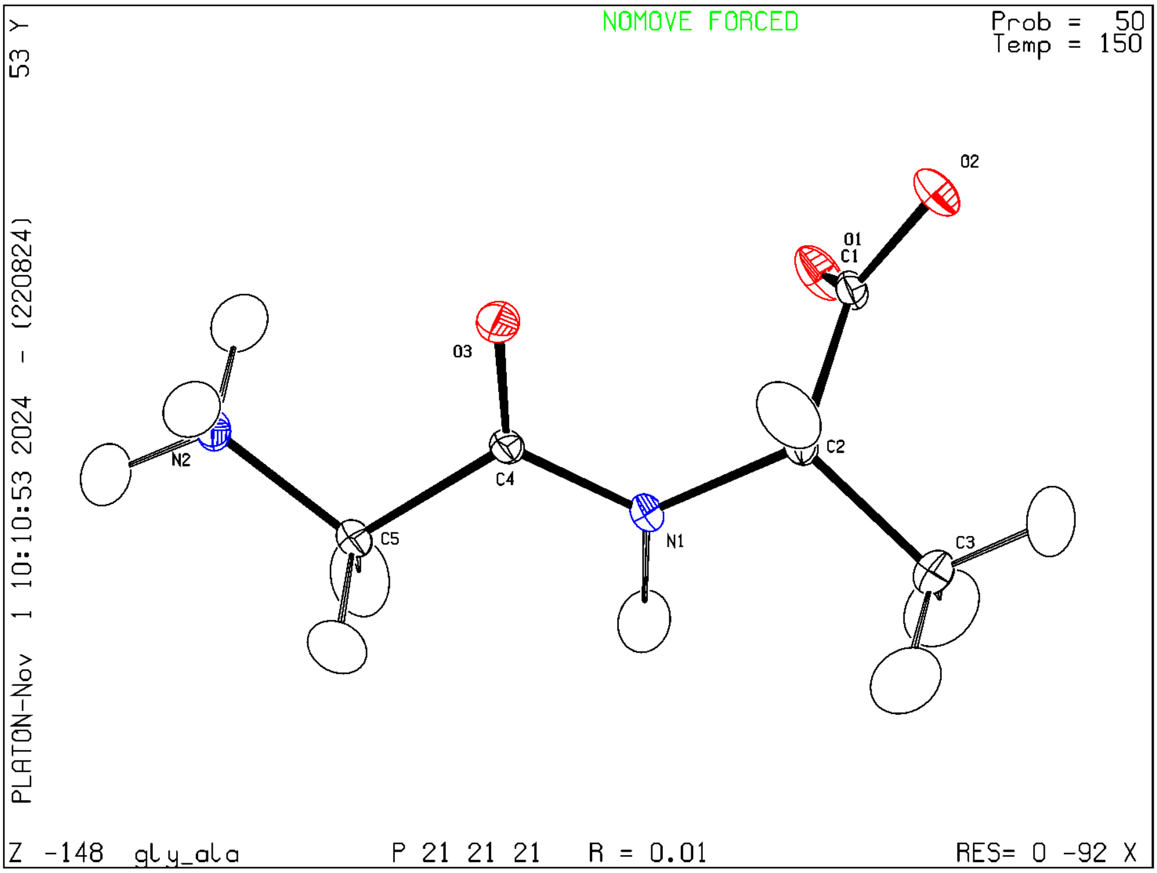

Supplement: Supplementary file 1 [file m-12-00074-sup1.zip › cif_checkcif/gly_ala/MP2/1_d0.8_checkcif.pdf]
