# Supplementary material for: Towards improved accuracy of Hirshfeld atom refinement with an alternative electron density partition
Source: IUCrJ. 2025 Jan 1;12(Pt 1):74–87. doi: 10.1107/S2052252524011242 (PMC11707693; doi:10.1107/S2052252524011242)

checkCIF/PLATON report

Structure factors have been supplied for datablock(s) L-Ala_23K_HAR

THIS REPORT IS FOR GUIDANCE ONLY. IF USED AS PART OF A REVIEW PROCEDURE FOR PUBLICATION, IT SHOULD NOT REPLACE THE EXPERTISE OF AN EXPERIENCED CRYSTALLOGRAPHIC REFEREE.

No syntax errors found. CIF dictionary Interpreting this report

Datablock: L-Ala_23K_HAR

```
Bond precision:   C-C = 0.0005 A          Wavelength=0.71073

Cell:              a=5.9279(10)      b=12.2597(17)      c=5.7939(9)
                   alpha=90          beta=90            gamma=90
Temperature:       23 K

                 Calculated                    Reported
Volume           421.07(11)                    421.07(11)
Space group      P 21 21 21                    P 21 21 21
Hall group       P 2ac 2ab                     P 2ac 2ab
Moiety formula   C3 H7 N O2                    C3 H7 N O2
Sum formula      C3 H7 N O2                    C3 H7 N O2
Mr               89.10                         89.10
Dx,g cm-3        1.406                         1.405
Z                4                             4
Mu (mm-1)        0.117                         0.117
F000             192.0                         192.0
F000'            192.11
h,k,lmax         12,26,12                      12,26,12
Nref             4445[ 2536]                   2405
Tmin,Tmax
Tmin'

Correction method= Not given

Data completeness= 0.95/0.54       Theta(max)= 50.140

R(reflections)= 0.0184( 2372)            wR2(reflections)= 0.0315( 2405)

S = 1.091                  Npar= 118
```

```
The following ALERTS were generated. Each ALERT has the format
       test-name_ALERT_alert-type_alert-level.
Click on the hyperlinks for more details of the test.
```

Alert level C

```
CELLK01_ALERT_1_C  Check that the cell measurement temperature is in Kelvin.
            Value of measurement temperature given =    23.000
CRYSR01_ALERT_1_C  _exptl_crystal_size_rad not in the CIF when expected.
PLAT029_ALERT_3_C _diffrn_measured_fraction_theta_full value Low .      0.973 Why?
PLAT053_ALERT_1_C Minimum Crystal Dimension Missing (or Error) ...     Please Check
PLAT054_ALERT_1_C Medium  Crystal Dimension Missing (or Error) ...     Please Check
PLAT055_ALERT_1_C Maximum Crystal Dimension Missing (or Error) ...     Please Check
PLAT353_ALERT_3_C Long   N-H (N0.87,N1.01A)  N1       - H1       .       1.02 Ang.
PLAT353_ALERT_3_C Long   N-H (N0.87,N1.01A)  N1       - H2       .       1.02 Ang.
PLAT353_ALERT_3_C Long   N-H (N0.87,N1.01A)  N1       - H3       .       1.05 Ang.
PLAT911_ALERT_3_C Missing FCF Refl Between Thmin & STh/L=    0.600         13 Report
                7  1  0,   3  2  0,   5  6  0,   0  5  1,   1 12  1,   3  0  2,
                5  2  2,   5  0  4,   0  0  6,   1  0  6,   3  0  6,   1  1  6,
                2  6  6,
```

Alert level G

```
PLAT881_ALERT_1_G No Datum for _diffrn_reflns_av_R_equivalents ...     Please Do !
PLAT883_ALERT_1_G No Info/Value for _atom_sites_solution_primary .     Please Do !
PLAT912_ALERT_4_G Missing # of FCF Reflections Above STh/L=  0.600        118 Note
PLAT961_ALERT_5_G Dataset Contains no Negative Intensities ......     Please Check
PLAT969_ALERT_5_G The 'Henn et al.' R-Factor-gap value ..........       1.11 Note
              Predicted wR2: Based on SigI**2  2.83 or SHELX Weight  2.96
PLAT978_ALERT_2_G Number C-C Bonds with Positive Residual Density.          2 Info
PLAT979_ALERT_1_G NoSpherA2 Scattering Factors Used .............     Please Note
```

```
   0 ALERT level A = Most likely a serious problem - resolve or explain
   0 ALERT level B = A potentially serious problem, consider carefully
  10 ALERT level C = Check. Ensure it is not caused by an omission or oversight
   7 ALERT level G = General information/check it is not something unexpected

   8 ALERT type 1 CIF construction/syntax error, inconsistent or missing data
   1 ALERT type 2 Indicator that the structure model may be wrong or deficient
   5 ALERT type 3 Indicator that the structure quality may be low
   1 ALERT type 4 Improvement, methodology, query or suggestion
   2 ALERT type 5 Informative message, check
```

It is advisable to attempt to resolve as many as possible of the alerts in all categories. Often the minor alerts point to easily fixed oversights, errors and omissions in your CIF or refinement strategy, so attention to these fine details can be worthwhile. In order to resolve some of the more serious problems it may be necessary to carry out additional measurements or structure refinements. However, the purpose of your study may justify the reported deviations and the more serious of these should normally be commented upon in the discussion or experimental section of a paper or in the "special_details" fields of the CIF. checkCIF was carefully designed to identify outliers and unusual parameters, but every test has its limitations and alerts that are not important in a particular case may appear. Conversely, the absence of alerts does not guarantee there are no aspects of the results needing attention. It is up to the individual to critically assess their own results and, if necessary, seek expert advice.

Publication of your CIF in IUCr journals

A basic structural check has been run on your CIF. These basic checks will be run on all CIFs submitted for publication in IUCr journals (*Acta Crystallographica*, *Journal of Applied Crystallography*, *Journal of Synchrotron Radiation*); however, if you intend to submit to *Acta Crystallographica Section C* or *E* or *IUCrData*, you should make sure that full publication checks are run on the final version of your CIF prior to submission.

Publication of your CIF in other journals

Please refer to the *Notes for Authors* of the relevant journal for any special instructions relating to CIF submission.

PLATON version of 06/01/2024; check.def file version of 05/01/2024

Datablock L-Ala_23K_HAR - ellipsoid plot

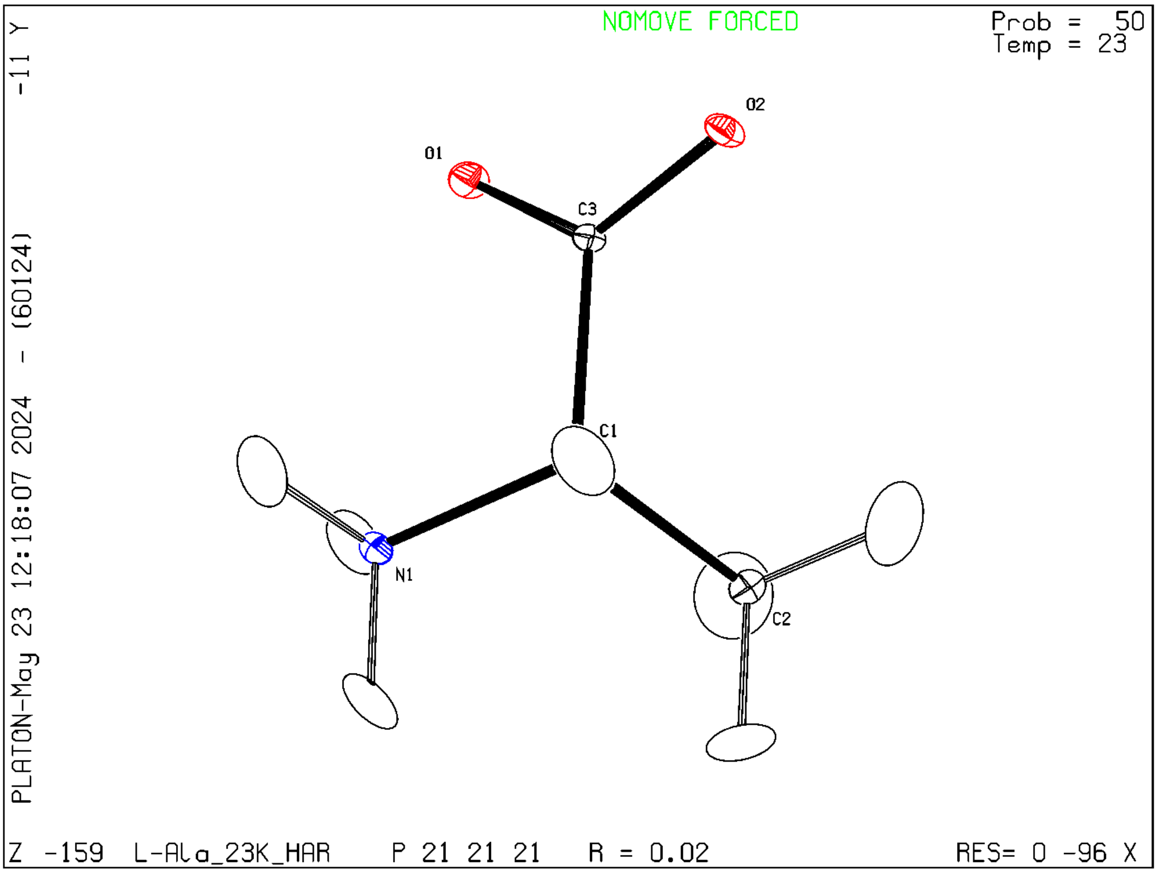

Supplement: Supplementary file 1 [file m-12-00074-sup1.zip › cif_checkcif/l_ala/MP2/1_checkcif.pdf]
