# Supplementary material for: Towards improved accuracy of Hirshfeld atom refinement with an alternative electron density partition
Source: IUCrJ. 2025 Jan 1;12(Pt 1):74–87. doi: 10.1107/S2052252524011242 (PMC11707693; doi:10.1107/S2052252524011242)
Supplement: Supplementary file 2 [file m-12-00074-sup2.pdf]

IUCrJ

Volume 12 (2025)

Supporting information for article:

Towards improved accuracy of Hirshfeld Atom Refinement with an alternative electron density partition

Michał Chodkiewicz and Krzysztof Woźniak

S1. MSD_{corr} calculation 1

S2. Calculation of form factor ratio for aspherical form factors for urea 2

S3. Values of wR_2 calculated for all reflections used in refinement 2

S4. Values of ⟨|Δd|⟩ 3

S5. Values of ⟨Δd⟩ 6

S6. Values of MSD_{corr} 8

S7. Values of $\langle \boldsymbol{\eta r} \rangle$ 10

S8. Values of U_{eq} ratio for expHAR and neutron derived structures 12

S9. Values of U_{eq} ratio for expHAR and HAR derived structures 15

S10. Influence of resolution on refinement – test with B3LYP and with MP2 16

S11. Atomic charges from exponential Hirshfeld partition 17

S11.1. Carbamazepine 17

S11.2. Gly-L-Ala 19

S11.3. Urea 20

S11.4. Xylitol 21

S1. MSD_{corr} calculation

Covariance can be expressed in terms of expected values: cov(X, Y) = E[XY]-E[X]E[Y]. In the case of MSDs, E[MSD] corresponds to the average MSD which is equal to U_{eq}. The E[XY] term can be expressed by averaging the MSDs product over all possible displacement directions $\boldsymbol{r}$ i.e. all vectors on a unit sphere:

$$\langle \mathrm{MSD}(U_A)\mathrm{MSD}(U_B)\rangle = \frac{1}{4\pi}\int_S (\boldsymbol{r}^{\mathrm{T}}\boldsymbol{U}_A\boldsymbol{r})(\boldsymbol{r}^{\mathrm{T}}\boldsymbol{U}_B\boldsymbol{r})\mathrm{d}S$$

After integration, it can be expressed in terms of a trace of a matrix (denoted as Tr(M) = $M_{11}+M_{22}+M_{33}$) as:

$$\langle \mathrm{MSD}(U_A)\mathrm{MSD}(U_B)\rangle = \frac{2}{15}\left(\mathrm{Tr}(\boldsymbol{U}_A\boldsymbol{U}_B) - \frac{1}{3}\mathrm{Tr}(\boldsymbol{U}_A)\mathrm{Tr}(\boldsymbol{U}_B)\right)$$

and the MDS_{corr} is given by:

$$\mathrm{MSD_{corr}} = \frac{\mathrm{Tr}(U_A U_B) - \frac{1}{3}\mathrm{Tr}(U_A)\mathrm{Tr}(U_B)}{\sqrt{\left(\mathrm{Tr}(U_A^2) - \frac{\mathrm{Tr}^2(U_A)}{3}\right)\left(\mathrm{Tr}(U_A^2) - \frac{\mathrm{Tr}^2(U_B)}{3}\right)}}$$

or in terms of Frobenius norm ($\|A\|_F = \left(\sum_{ij} A_{ij}^2\right)^{1/2}$) and inner product ($\langle A, B\rangle_F = \sum_{ij} A_{ij}\, B_{ij}$) as:

$$\mathrm{MSD_{corr}} = \frac{\langle \boldsymbol{U}_\mathrm{A}, \boldsymbol{U}_\mathrm{B}\rangle_\mathrm{F} - 3U_A^{eq}U_B^{eq}}{\sqrt{\left(\|\boldsymbol{U}_A\|_F^2 - 3\left(U_A^{eq}\right)^2\right)\left(\|\boldsymbol{U}_B\|_F^2 - 3\left(U_B^{eq}\right)^2\right)}}$$

S2. Calculation of form factor ratio for aspherical form factors for urea

Atomic form factors for atoms in urea structure are calculated using a geometry of urea molecule obtained with HAR involving wavefunction calculation with B3LYP functional, cc-pVTZ basis set, and surrounding point charges to mimic crystal field. The form factors are based on the Hirshfeld partition i.e. they are calculated in the same way as in HAR.

In the case of aspherical atomic electron densities, the form factors are also aspherical and they are complex numbers. To calculate the ratio of the form factors, in the first step an average module of the absolute value of the form factor for given length of the reciprocal space vector s is calculated using numerical integration: $f_C(\mathrm{s}) = \sum_k |f_C(\mathrm{s}\boldsymbol{r}_k)|\, w_k$, using 50-point Lebedev-Laikov grid, $f_C(\boldsymbol{s})$ is a value of aspherical atomic form factor (for carbon in this case) at reciprocal space vector $\boldsymbol{s}$, w_k are integration weights and $\boldsymbol{r}_k$ are the grid points in angular integration. The final ratio is calculated as a ratio of the average modules calculated in the first step, e.g. $f_C(\mathrm{s})/f_H(\mathrm{s})$. The values were calculated for sin(θ)/λ ranging from 0 to 1.4 Å^{-1} every 0.05 Å^{-1}.

S3. Values of wR_2 calculated for all reflections used in refinement

Table S1 Values of wR2 [%] calculated for all reflections used in refinement (B3LYP refinements with expHAR(n))

n	Carbam.	Gly- L-Ala	Ice	L-Ala	Oxa	BIPa	NAC·H_2O	8HQ HM	urea	xylitol
1	6.54	2.67	2.54	3.97	3.83	9.25	4.91	5.29	3.55	2.84
1.25	6.55	2.69	2.55	3.96	3.84	9.25	4.92	5.29	3.5	2.84
1.5	6.56	2.7	2.55	3.95	3.85	9.26	4.91	5.3	3.52	2.85
2	6.55	2.71	2.56	3.97	3.86	9.27	4.92	5.3	3.5	2.87
3	6.61	2.73	2.56	3.94	3.88	9.28	4.94	5.31	3.53	2.88
4	6.61	2.74	2.56	3.93	3.88	9.29	4.95	5.31	3.53	2.89

Table S2 Values of wR2 [%] calculated for all reflections used in refinement (MP2 refinements with expHAR(n))

n	Carbam.	Gly- L-Ala	Ice	L-Ala	Oxa	NAC·H_2O	8HQ HM	urea	xylitol
1	6.67	2.69	2.51	3.15	3.82	4.81	5.25	3.63	2.85
1.25	6.69	2.7	2.51	3.16	3.83	4.83	5.26	3.58	2.85
1.5	6.71	2.71	2.51	3.16	3.84	4.83	5.24	3.6	2.86
2	6.72	2.72	2.52	3.17	3.85	4.83	5.24	3.6	2.87
3	6.74	2.73	2.52	3.97	3.86	4.84	5.24	3.6	2.89
4	6.74	2.74	2.52	3.97	3.86	4.85	5.25	3.6	2.89

S4. Values of ⟨|Δd|⟩

Table S3 Values of average absolute deviation from neutron bond length (⟨|Δd|⟩) (mÅ) for polar X-H bonds, B3LYP expHAR(n) refinements.

n	Carbam.	Gly- L-Ala	Ice	L-Ala	Oxa	BIPa	NAC· H_2O	8HQ HM	urea	xylitol
1	19.8	7.1	15	19	9.2	16.4	39.2	27.1	3.4	17.8
1.25	11.5	5.7	12.9	13.2	3.9	9.7	29.1	20.7	4.6	10.3
1.5	8.5	5	11.2	9.5	2.8	7.3	23.1	17.6	6.2	8.1
2	8.8	5.3	8.6	6.9	2.8	6.8	16.6	14.5	7.5	7.4
3	7.8	7.5	4.3	9.4	5.2	10.5	12.4	13	10.7	6.9
4	9.7	8.1	3.9	10.3	6.1	12	10.8	12.6	11.4	7

Table S4 Values of average absolute deviation from neutron bond length (⟨|Δd|⟩) (mÅ) for C-H bonds, B3LYP expHAR(n) refinements.

n	Carbamazepine	Gly- L-Ala	L-Ala	BIPa	NAC·H_2O	8HQ HM	xylitol
1	5.3	7.1	5.5	13.7	16	8.3	8.8
1.25	5.3	6.9	3.8	10.8	12.7	7.6	8.4
1.5	5.7	6.9	3.1	10	10.6	7.3	8.3
2	3.9	7.8	3.6	9.2	8.4	7.6	8.2
3	5.8	9.1	5.5	8.8	7.1	7.4	8.2
4	5.7	9.3	6.1	8.7	6.7	7.5	8.2

Table S5 Values of average absolute deviation from neutron bond length (⟨|Δd|⟩) (mÅ) for X-H bonds, B3LYP expHAR(n) refinements.

n	Carbam.	Gly- L-Ala	Ice	L-Ala	Oxa	BIPa	NAC·H_2O	8HQ HM	urea	xylitol
1	7.7	7.1	15	11.3	9.2	14.1	23.2	13.4	3.4	12.6
1.25	6.3	6.4	12.9	7.8	3.9	10.7	17.7	11.1	4.6	9.2
1.5	6.1	6.2	11.2	5.9	2.8	9.6	14.4	10.1	6.2	8.2
2	4.7	6.8	8.6	5	2.8	8.8	10.9	9.5	7.5	7.8
3	6.1	8.5	4.3	7.2	5.2	9.1	8.8	8.9	10.7	7.6
4	6.4	8.8	3.9	7.9	6.1	9.3	8	8.9	11.4	7.7

Table S6 Values of average absolute deviation from neutron bond length (⟨|Δd|⟩) (mÅ) for polar X-H bonds, MP2 expHAR(n) refinements.

n	Carbam.	Gly- L-Ala	Ice	L-Ala	Oxa	NAC·H_2O	8HQ HM	urea	Xylitol
1	16.1	6.4	4	14.8	4	31.3	21.9	4.7	10.2
1.25	9.6	6.6	0.9	9.5	2.5	25.1	16.6	6.7	7.3
1.5	8.3	7.4	2.9	6.6	2.8	19.7	13.8	8.4	6.5
2	7.8	9.8	5.6	6.8	4.8	13.6	12.5	10.3	6.8
3	9.5	11.7	9.2	13	6.8	8.8	11.5	12.5	8.7
4	11.3	12	9.6	13.6	7	8	11.3	12.8	9.1

Table S7 Values of average absolute deviation from neutron bond length (⟨|Δd|⟩) (mÅ) for C-H bonds, MP2 expHAR(n) refinements.

N	Carbamazepine	Gly- L-Ala	L-Ala	NAC·H_2O	8HQ HM	xylitol
1	4.5	8.5	7.4	13.6	6.6	9.3
1.25	5.8	9.7	5.5	11.2	5.9	8.8
1.5	6.6	10.6	4.3	9.5	5.6	8.6
2	6.3	11.7	4.3	7.5	5.5	8.5
3	6	12.6	9.5	6.3	5.4	8.4
4	6	12.8	10.3	6.2	5.4	8.4

Table S8 Values of average absolute deviation from neutron bond length (⟨|Δd|⟩) (mÅ) for X-H bonds, MP2 expHAR(n) refinements.

N	Carbam.	Gly- L-Ala	Ice	L-Ala	Oxa	NAC·H_2O	8HQ HM	urea	xylitol
1	6.5	7.7	4	10.5	4	19	10.8	4.7	9.7
1.25	6.4	8.5	0.9	7.2	2.5	15.5	8.9	6.7	8.2
1.5	6.9	9.4	2.9	5.3	2.8	12.6	7.9	8.4	7.7
2	6.5	10.9	5.6	5.4	4.8	9.4	7.4	10.3	7.8
3	6.6	12.2	9.2	11	6.8	7.1	7.1	12.5	8.5
4	6.9	12.5	9.6	11.7	7	6.8	7	12.8	8.7

S5. Values of ⟨Δd⟩

Table S9 Values of average deviation from neutron bond length (⟨Δd⟩) (mÅ) for polar X-H bonds, B3LYP expHAR(n) refinements.

n	Carbam.	Gly- L-Ala	Ice	L-Ala	Oxa	BIPa	NAC·H_2O	8HQ HM	urea	xylitol
1	-19.8	-4.5	-15	-19	-9.2	-13	-39.2	-27.1	1.2	-17.8
1.25	-11.5	0.2	-12.9	-13.2	-3.4	-4.4	-29.1	-20.7	4.1	-10.3
1.5	-6	2.1	-11.2	-9.5	-0.3	0.3	-23.1	-17.6	6.2	-5.8
2	-6.4	4.8	-8.6	-5.7	2.1	6	-16.6	-14.5	7.5	-1.5
3	7.8	7.5	-4.3	-1	5.2	10.5	-12.4	-11.5	10.7	3
4	9.7	8.1	-3.9	0.6	6.1	12	-10.8	-10.9	11.4	4.3

Table S10 Values of average deviation from neutron bond length (⟨Δd⟩) (mÅ) for C-H bonds, B3LYP expHAR(n) refinements.

n	Carbamazepine	Gly- L-Ala	L-Ala	BIPa	NAC·H_2O	8HQ HM	xylitol
1	-3.2	5.2	-0.8	-5.7	-15.7	-7.1	-4.2
1.25	-1	6.6	1.5	-2.6	-12.4	-6.2	-3.1
1.5	0	6.9	2.7	-1.1	-10.3	-6	-2.8
2	-0.8	7.8	3.6	0.7	-7.4	-6.5	-3
3	1	9.1	5.5	2.1	-5.1	-6.4	-2
4	0.9	9.3	6.1	2.6	-4.2	-6.7	-1.9

Table S11 Values of average deviation from neutron bond length (⟨Δd⟩) (mÅ) for X-H bonds, B3LYP expHAR(n) refinements.

n	Carbam.	Gly- L-Ala	Ice	L-Ala	Oxa	BIPa	NAC·H_2O	8HQ HM	urea	xylitol
1	7.7	7.1	15	11.3	9.2	14.1	23.2	13.4	3.4	12.6
1.25	6.3	6.4	12.9	7.8	3.9	10.7	17.7	11.1	4.6	9.2
1.5	6.1	6.2	11.2	5.9	2.8	9.6	14.4	10.1	6.2	8.2
2	4.7	6.8	8.6	5	2.8	8.8	10.9	9.5	7.5	7.8
3	6.1	8.5	4.3	7.2	5.2	9.1	8.8	8.9	10.7	7.6
4	6.4	8.8	3.9	7.9	6.1	9.3	8	8.9	11.4	7.7

Table S12 Values of average deviation from neutron bond length (⟨Δd⟩) (mÅ) for polar X-H bonds, MP2 expHAR(n) refinements.

N	Carbam.	Gly- L-Ala	Ice	L-Ala	Oxa	NAC·H_2O	8HQ HM	urea	xylitol
1	16.1	6.4	4	14.8	4	31.3	21.9	4.7	10.2
1.25	9.6	6.6	0.9	9.5	2.5	25.1	16.6	6.7	7.3
1.5	8.3	7.4	2.9	6.6	2.8	19.7	13.8	8.4	6.5
2	7.8	9.8	5.6	6.8	4.8	13.6	12.5	10.3	6.8
3	9.5	11.7	9.2	13	6.8	8.8	11.5	12.5	8.7
4	11.3	12	9.6	13.6	7	8	11.3	12.8	9.1

Table S13 Values of average deviation from neutron bond length (⟨Δd⟩) (mÅ) for C-H bonds, MP2 expHAR(n) refinements.

n	Carbamazepine	Gly- L-Ala	L-Ala	NAC·H_2O	8HQ HM	xylitol
1	4.5	8.5	7.4	13.6	6.6	9.3
1.25	5.8	9.7	5.5	11.2	5.9	8.8
1.5	6.6	10.6	4.3	9.5	5.6	8.6
2	6.3	11.7	4.3	7.5	5.5	8.5
3	6	12.6	9.5	6.3	5.4	8.4
4	6	12.8	10.3	6.2	5.4	8.4

Table S14 Values of average deviation from neutron bond length (⟨Δd⟩) (mÅ) for X-H bonds, B3LYP expHAR(n) refinements.

n	Carbam.	Gly- L-Ala	Ice	L-Ala	Oxa	NAC·H_2O	8HQ HM	urea	xylitol
1	6.5	7.7	4	10.5	4	19	10.8	4.7	9.7
1.25	6.4	8.5	0.9	7.2	2.5	15.5	8.9	6.7	8.2
1.5	6.9	9.4	2.9	5.3	2.8	12.6	7.9	8.4	7.7
2	6.5	10.9	5.6	5.4	4.8	9.4	7.4	10.3	7.8
3	6.6	12.2	9.2	11	6.8	7.1	7.1	12.5	8.5
4	6.9	12.5	9.6	11.7	7	6.8	7	12.8	8.7

S6. Values of MSD_{corr}

Table S15 Values of average MSD_{corr} for polar H-atoms, B3LYP expHAR(n) refinements.

n	Carbam.	Gly-Ala	Ice	L-Ala	Oxa	BIPa	NAC· H_2O	8HQ HM	Urea	xylitol
1	0.874	0.524	0.541	0.271	0.744	0.188	0.08	0.513	0.986	0.568
1.25	0.961	0.58	0.506	0.375	0.806	0.408	0.228	0.603	0.987	0.686
1.5	0.984	0.609	0.498	0.451	0.826	0.516	0.363	0.659	0.987	0.749
2	0.983	0.649	0.522	0.555	0.841	0.643	0.562	0.725	0.987	0.808
3	0.99	0.682	0.562	0.671	0.854	0.728	0.737	0.788	0.986	0.859
4	0.987	0.693	0.586	0.711	0.859	0.756	0.79	0.811	0.985	0.872

Table S16 Values of average MSD_{corr} for non-polar H-atoms, B3LYP expHAR(n) refinements.

n	Carbamazepine	Gly- L-Ala	L-Ala	BIPa	NAC·H_2O	8HQ HM	xylitol
1	0.889	0.94	0.706	0.795	0.754	0.932	0.778
1.25	0.916	0.936	0.74	0.818	0.798	0.935	0.809
1.5	0.931	0.933	0.762	0.831	0.827	0.939	0.829
2	0.942	0.928	0.788	0.844	0.862	0.944	0.852
3	0.948	0.922	0.812	0.854	0.889	0.948	0.864
4	0.949	0.919	0.819	0.857	0.897	0.95	0.868

Table S17 Values of average MSD_{corr} for H-atoms, B3LYP expHAR(n) refinements.

n	Carbam.	Gly- Ala	Ice	L-Ala	Oxa	BIPa	NAC·H_2O	8HQ HM	urea	xylitol
1	0.887	0.774	0.541	0.519	0.744	0.698	0.547	0.817	0.986	0.691
1.25	0.924	0.794	0.506	0.583	0.806	0.753	0.622	0.845	0.987	0.758
1.5	0.94	0.803	0.498	0.629	0.826	0.78	0.685	0.863	0.987	0.796
2	0.949	0.816	0.522	0.688	0.841	0.812	0.77	0.884	0.987	0.833
3	0.955	0.826	0.562	0.751	0.854	0.834	0.842	0.905	0.986	0.862
4	0.955	0.828	0.586	0.773	0.859	0.841	0.864	0.912	0.985	0.87

Table S18 Values of average MSD_{corr} for polar H-atoms, MP2 expHAR(n) refinements.

n	Carbam.	Gly-Ala	Ice	L-Ala	Oxa	NAC·H_2O	8HQ HM	urea	xylitol
1	0.842	0.457	0.474	0.004	0.765	0.082	0.534	0.988	0.507
1.25	0.943	0.508	0.489	0.096	0.81	0.122	0.615	0.988	0.625
1.5	0.974	0.539	0.499	0.192	0.827	0.236	0.667	0.987	0.698
2	0.99	0.578	0.532	0.358	0.843	0.463	0.73	0.986	0.776
3	0.99	0.606	0.565	0.596	0.855	0.645	0.785	0.985	0.825
4	0.989	0.621	0.588	0.643	0.86	0.72	0.808	0.984	0.842

Table S19 Values of average MSD_{corr} for non-polar H-atoms, MP2 expHAR(n) refinements.

n	Carbamazepine	Gly- L-Ala	L-Ala	NAC·H_2O	8HQ HM	xylitol
1	0.882	0.944	0.705	0.724	0.925	0.753
1.25	0.912	0.941	0.746	0.759	0.931	0.791
1.5	0.927	0.938	0.774	0.797	0.936	0.816
2	0.94	0.933	0.807	0.848	0.941	0.841
3	0.947	0.928	0.799	0.881	0.946	0.858
4	0.949	0.925	0.809	0.892	0.948	0.863

Table S20 Values of average MSD_{corr} for H-atoms, MP2 expHAR(n) refinements.

n	Carbam.	Gly-Ala	Ice	L-Ala	Oxa	NAC· H_2O	8HQ HM	urea	xylitol
1	0.842	0.457	0.474	0.004	0.765	0.082	0.534	0.988	0.507
1.25	0.943	0.508	0.489	0.096	0.81	0.122	0.615	0.988	0.625
1.5	0.974	0.539	0.499	0.192	0.827	0.236	0.667	0.987	0.698
2	0.99	0.578	0.532	0.358	0.843	0.463	0.73	0.986	0.776
3	0.99	0.606	0.565	0.596	0.855	0.645	0.785	0.985	0.825
4	0.989	0.621	0.588	0.643	0.86	0.72	0.808	0.984	0.842

S7. Values of $\langle \eta_r \rangle$

Table S21 Values of average rescaled overlapping coefficient $\langle \eta_r \rangle$ for polar H-atoms, B3LYP expHAR(n) refinements.

n	Carbam.	Gly- L-Ala	Ice	L-Ala	Oxa	BIPa	NAC· H_2O	8HQ HM	urea	xylitol
1	17.8	10.8	7.2	41.3	16	19.4	22.9	22.9	7.5	16.8
1.25	12.9	11.8	6.9	31.9	13.6	15.3	19.7	19.8	6.2	13.5
1.5	9.5	12.5	6.8	29.2	12.9	13.6	17.5	18	5.9	11.6
2	5.3	13.5	6.6	27.1	12.7	12	14.8	16	6.8	9.6
3	4.8	14.4	6.4	24.4	13.6	11.3	12.4	14.5	8.8	8.4
4	5.5	14.7	6.3	23.9	14	11.1	11.9	14	9.7	8

Table S22 Values of average rescaled overlapping coefficient $\langle \eta_r \rangle$ for non-polar H-atoms, B3LYP expHAR(n) refinements.

n	Carbamazepine	Gly- L-Ala	L-Ala	BIPa	NAC·H_2O	8HQ HM	xylitol
1	8.7	11.5	25.3	13.4	17.1	15.6	13.4
1.25	7.2	12.2	25.3	12.2	16.2	14	11.6
1.5	6.9	12.8	25.2	11.6	15.7	12.9	10.4
2	8.4	13.6	25	11.2	15.4	11.7	9.1
3	9.9	14.2	25.8	11.1	16.2	11	8.4
4	10.7	14.6	26.1	11.2	17	10.7	8.2

Table S23 Values of average rescaled overlapping coefficient $\langle \eta_r \rangle$ for H-atoms, B3LYP expHAR(n) refinements.

n	Carbam.	Gly- L-Ala	Ice	L-Ala	Oxa	BIPa	NAC·H_2O	8HQ HM	urea	xylitol
1	10.2	11.2	7.2	32.1	16	14.4	18.9	17.6	7.5	14.8
1.25	8.1	12	6.9	28.1	13.6	12.7	17.3	15.6	6.2	12.4
1.5	7.4	12.7	6.8	26.9	12.9	11.9	16.3	14.3	5.9	10.9
2	7.9	13.5	6.6	25.9	12.7	11.3	15.2	12.9	6.8	9.3
3	9	14.3	6.4	25.2	13.6	11.2	15	11.9	8.8	8.4
4	9.8	14.6	6.3	25.2	14	11.2	15.4	11.6	9.7	8.1

Table S24 Values of average rescaled overlapping coefficient $\langle \eta_r \rangle$ for polar H-atoms, MP2 expHAR(n) refinements.

n	Carbam.	Gly- L-Ala	Ice	L-Ala	Oxa	NAC·H_2O	8HQ HM	urea	xylitol
1	15.9	13	7	30.1	14	23	20.9	5.6	15.8
1.25	11.4	14.2	6.8	29.5	12.6	23	18.2	5.7	13
1.5	9.2	15.1	6.7	28.6	12.5	21.6	16.6	6.8	11.4
2	5.3	16	6.5	27.1	13.2	18.4	15.1	8.6	9.9
3	5.1	16.8	6.5	31.6	13.9	16.7	14.1	10.5	9.2
4	5.8	17.1	6.4	30.6	14.2	16	13.7	11.3	9

Table S25 Values of average rescaled overlapping coefficient $\langle \eta_r \rangle$ for non-polar H-atoms, MP2 expHAR(n) refinements.

n	Carbamazepine	Gly- L-Ala	L-Ala	NAC·H_2O	8HQ HM	xylitol
1	8.8	13.1	18.6	18.9	14.5	13.3
1.25	8.1	13.8	18.6	18.7	12.9	11.7
1.5	8.5	14.4	19	18.3	11.9	10.7
2	10	15.1	20.2	17.9	10.9	9.7
3	11.7	15.7	29	18.6	10.3	9.3
4	12.3	16	29	19.3	10.1	9.2

Table S26 Values of average rescaled overlapping coefficient $\langle \eta_r \rangle$ for H-atoms, MP2 expHAR(n) refinements.

n	Carbam.	Gly- L-Ala	Ice	L-Ala	Oxa	NAC·H_2O	8HQ HM	urea	xylitol
1	10	13.1	7	23.5	14	20.1	16.3	5.6	14.4
1.25	8.6	14	6.8	23.3	12.6	20.1	14.3	5.7	12.2
1.5	8.6	14.7	6.7	23.2	12.5	19.3	13.2	6.8	11
2	9.2	15.5	6.5	23.1	13.2	18	12	8.6	9.8
3	10.6	16.2	6.5	30.1	13.9	18	11.3	10.5	9.2
4	11.2	16.4	6.4	29.7	14.2	18.3	11.1	11.3	9.1

S8. Values of U_{eq} ratio for expHAR and neutron derived structures

The average U_{eq} ratio $\left\langle \frac{U_{eq}^{X}}{U_{eq}^{N}} \right\rangle$ is reported.

Table S27 Values of average U_{eq} ratio for expHAR and neutron-derived structures for polar H-atoms, B3LYP expHAR(n) refinements.

N	Carbam.	Gly- L-Ala	Ice	L-Ala	Oxa	BIPa	NAC· H_2O	8HQ HM	urea	xylitol
1	1.18	0.998	1.093	0.981	1.293	1.164	1.239	1.401	1.081	1.224
1.25	1.11	0.946	1.075	0.895	1.217	1.089	1.116	1.301	1.052	1.152
1.5	1.07	0.914	1.065	0.845	1.178	1.044	1.047	1.251	1.032	1.11
2	1.054	0.886	1.056	0.79	1.133	0.998	0.981	1.197	1.01	1.06
3	1.006	0.871	1.049	0.766	1.117	0.966	0.945	1.167	1	1.042
4	1.001	0.865	1.046	0.756	1.111	0.956	0.933	1.156	0.996	1.034

Table S28 Values of average U_{eq} ratio for expHAR and neutron-derived structures for non-polar H-atoms, B3LYP expHAR(n) refinements.

n	Carbamazepine	Gly- L-Ala	L-Ala	BIPa	NAC·H_2O	8HQ HM	xylitol
1	1.045	0.945	1.072	1.062	1.073	1.294	1.115
1.25	1.003	0.914	1.023	1.02	1.015	1.249	1.074
1.5	0.977	0.889	0.989	0.993	0.977	1.218	1.046
2	0.951	0.868	0.95	0.963	0.935	1.18	1.014
3	0.929	0.855	0.925	0.941	0.904	1.158	0.995
4	0.922	0.849	0.916	0.933	0.893	1.149	0.988

Table S29 Values of average U_{eq} ratio for expHAR and neutron-derived structures for H-atoms, B3LYP expHAR(n) refinements.

n	Carbam.	Gly- L-Ala	Ice	L-Ala	Oxa	BIPa	NAC·H_2O	8HQ HM	urea	xylitol
1	1.068	0.966	1.093	1.033	1.293	1.079	1.124	1.323	1.081	1.161
1.25	1.021	0.927	1.075	0.968	1.217	1.031	1.046	1.264	1.052	1.107
1.5	0.992	0.899	1.065	0.927	1.178	1.001	0.999	1.227	1.032	1.073
2	0.968	0.875	1.056	0.882	1.133	0.969	0.949	1.185	1.01	1.033
3	0.942	0.861	1.049	0.857	1.117	0.945	0.916	1.161	1	1.015
4	0.935	0.856	1.046	0.848	1.111	0.937	0.905	1.151	0.996	1.007

Table S30 Values of average U_{eq} ratio for expHAR and neutron-derived structures for polar H-atoms, MP2 expHAR(n) refinements.

n	Carbam.	Gly- L-Ala	Ice	L-Ala	Oxa	NAC·H_2O	8HQ HM	urea	xylitol
1	1.098	0.925	1.043	0.922	1.174	1.143	1.277	1.026	1.124
1.25	1.033	0.877	1.027	0.833	1.113	1.009	1.192	1.003	1.056
1.5	0.985	0.851	1.02	0.785	1.08	0.944	1.149	0.987	1.019
2	0.962	0.827	1.013	0.738	1.049	0.887	1.107	0.971	0.983
3	0.939	0.809	1.007	0.679	1.027	0.848	1.076	0.96	0.957
4	0.934	0.804	1.005	0.672	1.021	0.838	1.066	0.957	0.95

Table S31 Values of average U_{eq} ratio for expHAR and neutron-derived structures for non-polar H-atoms, MP2 expHAR(n) refinements.

n	Carbamazepine	Gly- L-Ala	L-Ala	NAC·H_2O	8HQ HM	xylitol
1	0.981	0.904	1.065	1.011	1.231	1.059
1.25	0.941	0.873	1.008	0.948	1.185	1.018
1.5	0.916	0.854	0.974	0.912	1.158	0.993
2	0.893	0.834	0.936	0.874	1.128	0.967
3	0.877	0.82	0.856	0.845	1.107	0.948
4	0.872	0.815	0.849	0.837	1.099	0.942

Table S32 Values of average U_{eq} ratio for expHAR and neutron-derived structures for H-atoms, MP2 expHAR(n) refinements.

n	Carbam.	Gly- L-Ala	Ice	L-Ala	Oxa	NAC·H_2O	8HQ HM	urea	xylitol
1	1.001	0.913	1.043	1.003	1.174	1.051	1.243	1.026	1.086
1.25	0.956	0.875	1.027	0.933	1.113	0.967	1.187	1.003	1.033
1.5	0.927	0.853	1.02	0.893	1.08	0.922	1.155	0.987	1.004
2	0.905	0.831	1.013	0.851	1.049	0.878	1.122	0.971	0.973
3	0.888	0.816	1.007	0.78	1.027	0.846	1.098	0.96	0.952
4	0.882	0.811	1.005	0.773	1.021	0.837	1.09	0.957	0.945

S9. Values of U_{eq} ratio for expHAR and HAR derived structures

The average U_{eq} ratio $\left\langle \frac{U_{eq}^{expHAR}}{U_{eq}^{HAR}} \right\rangle$ is reported.

Table S33 Values of average U_{eq} ratio for expHAR and HAR derived structures for polar H-atoms, B3LYP expHAR(n) refinements.

n	Carbam.	Gly- L-Ala	Ice	L-Ala	Oxa	BIPa	NAC· H_2O	8HQ HM	urea	xylitol
1	1.18	0.998	1.093	0.981	1.293	1.164	1.239	1.401	1.081	1.224
1.25	1.11	0.946	1.075	0.895	1.217	1.089	1.116	1.301	1.052	1.152
1.5	1.07	0.914	1.065	0.845	1.178	1.044	1.047	1.251	1.032	1.11
2	1.054	0.886	1.056	0.79	1.133	0.998	0.981	1.197	1.01	1.06
3	1.006	0.871	1.049	0.766	1.117	0.966	0.945	1.167	1	1.042
4	1.001	0.865	1.046	0.756	1.111	0.956	0.933	1.156	0.996	1.034

Table S34 Values of average U_{eq} ratio for expHAR and HAR derived structures for non-polar H-atoms, B3LYP expHAR(n) refinements.

n	Carbamazepine	Gly- L-Ala	L-Ala	BIPa	NAC·H_2O	8HQ HM	xylitol
1	1	1	1	1	1	1	1
1.25	0.96	0.967	0.954	0.96	0.946	0.965	0.963
1.5	0.935	0.941	0.923	0.935	0.911	0.941	0.938
2	0.91	0.919	0.886	0.907	0.871	0.912	0.909
3	0.889	0.905	0.863	0.886	0.842	0.895	0.892
4	0.882	0.898	0.854	0.879	0.832	0.888	0.886

S10. Influence of resolution on refinement – test with B3LYP and with MP2

Table S35 The scale parameter q for scaling low-resolution refinement (d_{min}=0.8Å) non-H ADPs to values from high-resolution refinement and the average atomic mean square displacement correlation $\langle MSD_{corr} \rangle$ for polar hydrogen atoms for the two refinements calculated with respect to neutron measurement data.

	q		$\langle MSD_{corr} \rangle$			
	B3LYP	MP2	B3LYP		MP2	
			max d=0.8	max d=d_{max}	max d=0.8	max d=d_{max}
Carbamazepine	0.864	0.854	0.982	0.874	0.971	0.842
NAC·H_2O	0.884	0.883	0.731	0.080	0.774	0.082
L-Ala	0.924	0.938	0.631	0.271	0.284	0.004
BIPa	0.931		0.416	0.188		
Oxa·2H_2O	0.968	0.966	0.685	0.744	0.718	0.765
8HQ HM	0.990	0.987	0.404	0.513	0.372	0.534
Ice VI	0.993	1.001	0.439	0.541	0.104	0.224
Urea	0.996	0.997	0.964	0.986	0.985	0.988
Xylitol	1.004	1.003	0.312	0.568	0.262	0.507
Gly-L-Ala	1.020	1.021	0.278	0.524	0.178	0.457

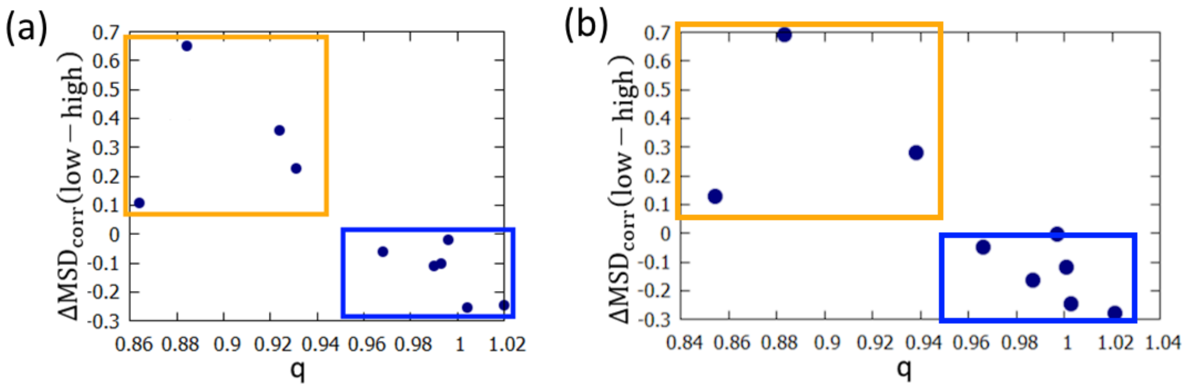

Figure S1 Change in polar H-atoms ADPs accuracy (measured with average MSD_{corr}) when data resolution is limited to d=0.8Å plotted against the scale parameter q for scaling low-resolution refinement (d_{min}=0.8Å) non-H ADPs to values from high-resolution refinement. Limiting resolution deteriorated the accuracy of the ADPs when the parameter q was close to 1 (points inside of the blue box) and improved otherwise (points inside of the orange box). Values for HAR refinements using as

computational method (a) B3LYP (b) MP2 (point for BIPa is missing since there was not MP2 refinement for this system).

S11. Atomic charges from exponential Hirshfeld partition

S11.1. Carbamazepine

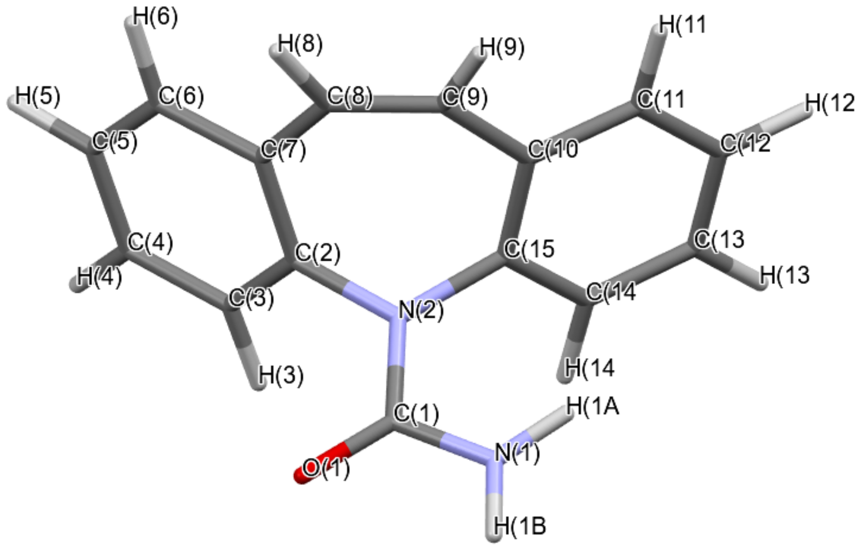

Figure S2 Atom labels for carbamazepine.

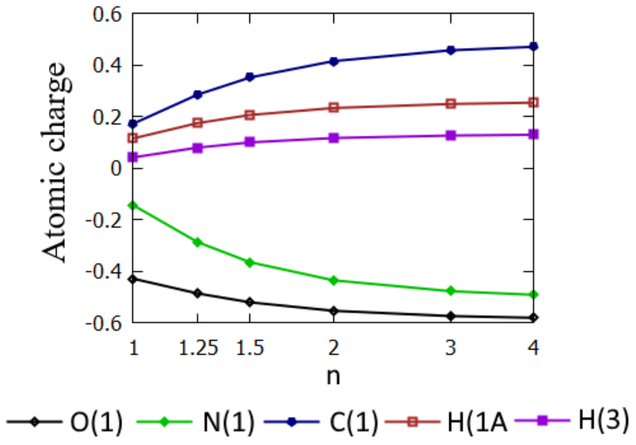

Figure S3 Atomic charge from exponential Hirshfeld partition (B3LYP) for carbamazepine as a function of an exponent parameter n for chosen atoms.

Table S36 Atomic charges from exponential Hirshfeld partition (B3LYP) for carbamazepine as a function of an exponent parameter n.

n	1	1.25	1.5	2	3	4
O(1)	-0.42964	-0.48693	-0.52160	-0.55409	-0.57461	-0.58132
N(1)	-0.14454	-0.28812	-0.36599	-0.43576	-0.47812	-0.49208
N(2)	-0.04913	-0.11511	-0.15693	-0.19529	-0.21988	-0.22785
C(1)	0.17182	0.28461	0.35112	0.41449	0.45638	0.47001
C(2)	0.04237	0.07028	0.08537	0.09911	0.10834	0.11131
C(3)	-0.04612	-0.08062	-0.09772	-0.11272	-0.12088	-0.12349
C(4)	-0.04872	-0.08176	-0.09896	-0.11379	-0.12178	-0.12434
C(5)	-0.04223	-0.08524	-0.10647	-0.12430	-0.13411	-0.13701
C(6)	-0.02912	-0.07207	-0.09396	-0.11368	-0.12564	-0.12968
C(7)	-0.00799	-0.00094	0.00219	0.00547	0.00899	0.01029
C(8)	-0.04452	-0.08096	-0.09931	-0.11579	-0.12462	-0.12772
C(9)	-0.03725	-0.07418	-0.09326	-0.10978	-0.12003	-0.12315
C(10	-0.00901	-0.00045	0.00331	0.00745	0.01139	0.01292
C(11	-0.02688	-0.06954	-0.09121	-0.11010	-0.12188	-0.12561
C(12	-0.01153	-0.07042	-0.09901	-0.12263	-0.13705	-0.14096
C(13	-0.01301	-0.06177	-0.08627	-0.10722	-0.12041	-0.12429
C(14	-0.04451	-0.08016	-0.09828	-0.11421	-0.12329	-0.12626
C(15	0.03378	0.06046	0.07445	0.08728	0.09601	0.09876
H(1A	0.11486	0.17402	0.20558	0.23252	0.24806	0.25328
H(1B	0.15697	0.22336	0.25908	0.29071	0.30897	0.31478
H(3)	0.04108	0.07899	0.09927	0.11606	0.12570	0.12852
H(4)	0.04034	0.08206	0.10327	0.12165	0.13210	0.13533
H(5)	0.04537	0.07595	0.09234	0.10630	0.11383	0.11610
H(6)	0.04709	0.08191	0.10009	0.11574	0.12467	0.12740
H(9)	0.05851	0.10130	0.12283	0.14147	0.15171	0.15498
H(11	0.04086	0.07786	0.09737	0.11444	0.12419	0.12726
H(12	0.06342	0.08566	0.09798	0.10785	0.11218	0.11341

H(13	0.06061	0.10322	0.12521	0.14508	0.15634	0.16014
H(14	0.03601	0.07873	0.10074	0.11923	0.12992	0.13324
H(8)	0.03245	0.06883	0.08735	0.10246	0.11099	0.11347

S11.2. Gly-L-Ala

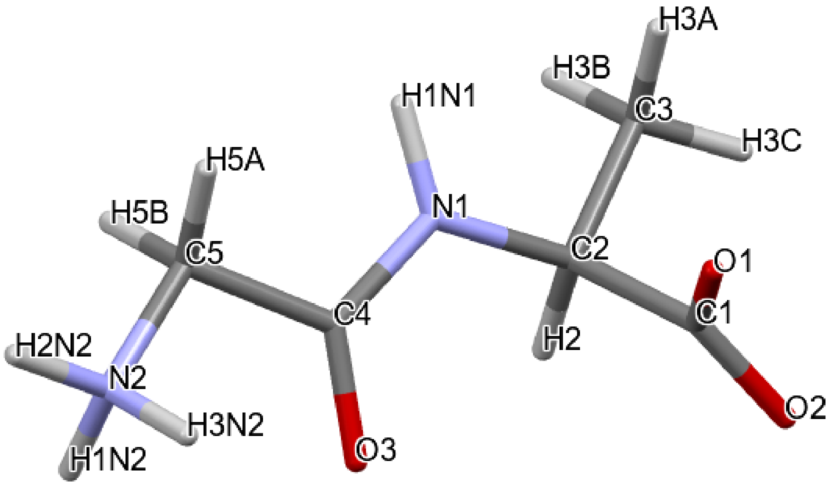

Figure S4 Atom labels for Gly-L-Ala

Table S37 Atomic charges from exponential Hirshfeld partition (B3LYP) for Gly-L-Ala as a function of an exponent parameter n.

n	1	1.25	1.5	2	3	4
O1	-0.53518	-0.59103	-0.63487	-0.66785	-0.67688	-0.68339
O2	-0.54704	-0.62183	-0.67698	-0.71612	-0.72813	-0.73608
O3	-0.32210	-0.39224	-0.44365	-0.47989	-0.49042	-0.49744
N1	-0.07463	-0.18202	-0.25090	-0.30685	-0.33681	-0.34880
N2	0.07030	-0.09882	-0.18816	-0.26565	-0.31194	-0.32671
C1	0.11603	0.23966	0.32772	0.39678	0.42239	0.43631
C2	0.01620	0.02411	0.03390	0.04402	0.04631	0.04981
C3	-0.07663	-0.18508	-0.23161	-0.27512	-0.30822	-0.31574
C4	0.16448	0.25159	0.30879	0.35633	0.37810	0.38775
C5	0.04038	-0.01350	-0.03000	-0.04791	-0.06704	-0.06943
H1N1	0.17190	0.22806	0.25928	0.28508	0.29930	0.30429
H1N2	0.23167	0.28438	0.31336	0.33721	0.35095	0.35559

H2N2	0.24227	0.29708	0.32436	0.34942	0.36520	0.37002
H3N2	0.22031	0.27705	0.30742	0.33250	0.34698	0.35170
H2	0.02301	0.05319	0.07337	0.08424	0.08756	0.08880
H3A	0.02447	0.05671	0.07313	0.08557	0.09339	0.09523
H3B	0.03925	0.07231	0.08733	0.10023	0.10972	0.11187
H3C	0.04401	0.07886	0.09777	0.11260	0.12127	0.12382
H5A	0.06712	0.09616	0.10874	0.11860	0.12623	0.12752
H5B	0.08516	0.12453	0.14240	0.15827	0.17037	0.17321

S11.3. Urea

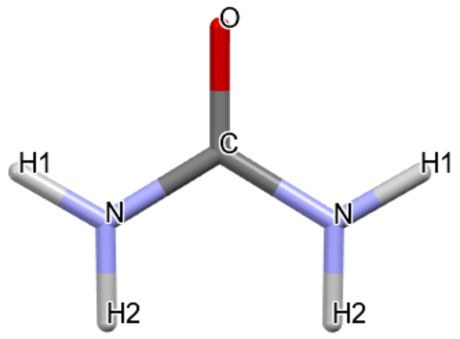

Figure S5 Atom labels for urea.

Table S38 Atomic charges from exponential Hirshfeld partition (B3LYP) for urea as a function of an exponent parameter n.

n	1	1.25	1.5	2	3	4
C	0.16194	0.28140	0.35192	0.43291	0.46655	0.48122
O	-0.53063	-0.58735	-0.62233	-0.66555	-0.67392	-0.68009
N	-0.13237	-0.28027	-0.36198	-0.44286	-0.48511	-0.50037
H1	0.15387	0.20916	0.24060	0.27251	0.28533	0.29055
H2	0.16309	0.22387	0.25633	0.28701	0.30312	0.30872

S11.4. Xylitol

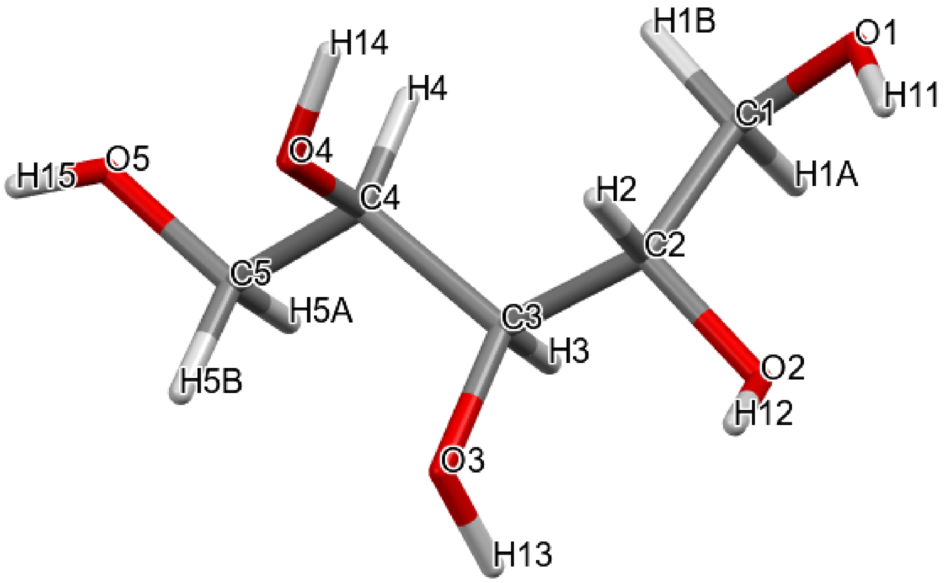

Figure S6 Atom labels for xylitol

Table S39 Atomic charges from exponential Hirshfeld partition (B3LYP) for xylitol as a function of an exponent parameter n.

n	1	1.25	1.5	2	3	4
O1	-0.29963	-0.41119	-0.47366	-0.54485	-0.56089	-0.57125
O2	-0.25996	-0.38498	-0.45263	-0.52675	-0.54734	-0.55856
O3	-0.27120	-0.38833	-0.45213	-0.52259	-0.54057	-0.55108
O4	-0.26945	-0.38847	-0.45313	-0.52444	-0.54313	-0.55382
O5	-0.28339	-0.39888	-0.46336	-0.53591	-0.55342	-0.56404
C1	0.01892	-0.00563	-0.01393	-0.00598	-0.01929	-0.01892
C2	0.04135	0.05557	0.06550	0.08622	0.08720	0.09102
C3	0.04645	0.05829	0.06653	0.08579	0.08459	0.08782
C4	0.04720	0.06021	0.06901	0.08857	0.08774	0.09102
C5	0.02177	-0.00363	-0.01223	-0.00430	-0.01705	-0.01644
H1A	0.02573	0.05692	0.07264	0.08414	0.09031	0.09182
H1B	0.04653	0.08332	0.10129	0.11393	0.12277	0.12480
H2	0.03236	0.06356	0.07961	0.09080	0.09778	0.09928
H3	0.04407	0.07711	0.09257	0.10141	0.10892	0.11009

H4	0.05328	0.08960	0.10693	0.11833	0.12615	0.12775
H5B	0.02458	0.05907	0.07726	0.09142	0.09989	0.10222
H5A	0.04090	0.07273	0.08814	0.09830	0.10475	0.10601
H11	0.18178	0.25447	0.29426	0.33757	0.34864	0.35485
H12	0.19100	0.26428	0.30385	0.34488	0.35849	0.36477
H13	0.19821	0.26899	0.30687	0.34573	0.35752	0.36318
H14	0.18372	0.25580	0.29458	0.33415	0.34751	0.35347
H15	0.18655	0.25997	0.30064	0.34470	0.35713	0.36367
