# Supplementary material for: Towards improved accuracy of Hirshfeld atom refinement with an alternative electron density partition
Source: IUCrJ. 2025 Jan 1;12(Pt 1):74–87. doi: 10.1107/S2052252524011242 (PMC11707693; doi:10.1107/S2052252524011242)

checkCIF/PLATON report

Structure factors have been supplied for datablock(s) 3

THIS REPORT IS FOR GUIDANCE ONLY. IF USED AS PART OF A REVIEW PROCEDURE FOR PUBLICATION, IT SHOULD NOT REPLACE THE EXPERTISE OF AN EXPERIENCED CRYSTALLOGRAPHIC REFEREE.

No syntax errors found. CIF dictionary Interpreting this report

Datablock: 3

Bond precision: = 0.0000 A Wavelength=0.56087

Cell:	a=6.1918(4)	b=6.1918(4)	c=5.7067(4)
	alpha=90	beta=90	gamma=90
Temperature:	293 K		

	Calculated	Reported
Volume	218.79(3)	218.79(2)
Space group	P 42/n m c	P 42/n m c
Hall group	-P 4ac 2a	-P 4ac 2a
Moiety formula	4(H2 O), H2 O	H2 O
Sum formula	H10 O5	H2 O
Mr	90.08	18.01
Dx,g cm-3	1.367	1.367
Z	2	10
Mu (mm-1)	0.089	0.089
F000	100.0	100.1
F000'	100.05	
h,k,lmax	9,9,9	9,6,9
Nref	270	240
Tmin,Tmax		
Tmin'		

Correction method= Not given

Data completeness= 0.889 Theta(max)= 26.660

R(reflections)= 0.0153(182) wR2(reflections)= 0.0256(240)

S = 0.932 Npar= 36

The following ALERTS were generated. Each ALERT has the format
test-name_ALERT_alert-type_alert-level.
Click on the hyperlinks for more details of the test.

Alert level A

```
PLAT029_ALERT_3_A _diffrn_measured_fraction_theta_full value Low .      0.918 Why?
```

Author Response: It is caused by the fact that sample was closed in Diamond Anvil Cell and as a result access to the reciprocal space was restricted.

Alert level B

```
PLAT088_ALERT_3_B Poor Data / Parameter Ratio ....................       6.67 Note
```

Alert level C

```
PLAT042_ALERT_1_C Calc. and Reported MoietyFormula Strings  Differ     Please Check
              Calc: 4(H2 O), H2 O
              Rep.: H2 O
PLAT053_ALERT_1_C Minimum Crystal Dimension Missing (or Error) ...     Please Check
PLAT054_ALERT_1_C Medium  Crystal Dimension Missing (or Error) ...     Please Check
PLAT055_ALERT_1_C Maximum Crystal Dimension Missing (or Error) ...     Please Check
PLAT313_ALERT_2_C Oxygen with Three Covalent Bonds (rare) .......          O1 Check
PLAT911_ALERT_3_C Missing FCF Refl Between Thmin & STh/L=    0.600         10 Report
                2  2  0,    0  4  0,    0  6  0,    1  7  0,    0  2  1,   1  2  1,
                0  6  1,    0  7  1,    0  1  2,    0  7  2,
PLAT913_ALERT_3_C Missing # of Very Strong Reflections in FCF ....          5 Note
                2  2  0,    0  4  0,    0  2  1,    1  2  1,    0  1  2,
```

Alert level G

```
PLAT017_ALERT_1_G Check Scattering Type Consistency of D1A      as          H
PLAT017_ALERT_1_G Check Scattering Type Consistency of D1B      as          H
PLAT017_ALERT_1_G Check Scattering Type Consistency of D2       as          H
PLAT017_ALERT_1_G Check Scattering Type Consistency of D1C      as          H
PLAT045_ALERT_1_G Calculated and Reported Z Differ by a Factor ...      0.200 Check
PLAT199_ALERT_1_G Reported _cell_measurement_temperature ..... (K)        293 Check
PLAT200_ALERT_1_G Reported   _diffrn_ambient_temperature ..... (K)        293 Check
PLAT300_ALERT_4_G Atom Site Occupancy of D1A        Constrained at        0.5 Check
PLAT300_ALERT_4_G Atom Site Occupancy of D1B        Constrained at        0.5 Check
PLAT300_ALERT_4_G Atom Site Occupancy of D1C        Constrained at        0.5 Check
PLAT300_ALERT_4_G Atom Site Occupancy of D2         Constrained at        0.5 Check
PLAT304_ALERT_4_G Non-Integer Number of Atoms in ..... (Resd    2)       2.62 Check
PLAT720_ALERT_4_G Number of Unusual/Non-Standard Labels ..........          4 Note
              D1A     D1B     D2      D1C
PLAT790_ALERT_4_G Centre of Gravity not Within Unit Cell: Resd.  #          2 Note
              H2 O
PLAT883_ALERT_1_G No Info/Value for _atom_sites_solution_primary .     Please Do !
PLAT912_ALERT_4_G Missing # of FCF Reflections Above STh/L=  0.600         21 Note
PLAT969_ALERT_5_G The 'Henn et al.' R-Factor-gap value ..........        1.44 Note
              Predicted wR2: Based on SigI**2  1.78 or SHELX Weight  2.98
PLAT979_ALERT_1_G NoSpherA2 Scattering Factors Used .............      Please Note
```

```
  1 ALERT level A = Most likely a serious problem - resolve or explain
  1 ALERT level B = A potentially serious problem, consider carefully
  7 ALERT level C = Check. Ensure it is not caused by an omission or oversight
 18 ALERT level G = General information/check it is not something unexpected

 13 ALERT type 1 CIF construction/syntax error, inconsistent or missing data
  1 ALERT type 2 Indicator that the structure model may be wrong or deficient
  4 ALERT type 3 Indicator that the structure quality may be low
  8 ALERT type 4 Improvement, methodology, query or suggestion
  1 ALERT type 5 Informative message, check
```

It is advisable to attempt to resolve as many as possible of the alerts in all categories. Often the minor alerts point to easily fixed oversights, errors and omissions in your CIF or refinement strategy, so attention to these fine details can be worthwhile. In order to resolve some of the more serious problems it may be necessary to carry out additional measurements or structure refinements. However, the purpose of your study may justify the reported deviations and the more serious of these should normally be commented upon in the discussion or experimental section of a paper or in the "special_details" fields of the CIF. checkCIF was carefully designed to identify outliers and unusual parameters, but every test has its limitations and alerts that are not important in a particular case may appear. Conversely, the absence of alerts does not guarantee there are no aspects of the results needing attention. It is up to the individual to critically assess their own results and, if necessary, seek expert advice.

Publication of your CIF in IUCr journals

A basic structural check has been run on your CIF. These basic checks will be run on all CIFs submitted for publication in IUCr journals (*Acta Crystallographica*, *Journal of Applied Crystallography*, *Journal of Synchrotron Radiation*); however, if you intend to submit to *Acta Crystallographica Section C* or *E* or *IUCrData*, you should make sure that full publication checks are run on the final version of your CIF prior to submission.

Publication of your CIF in other journals

Please refer to the *Notes for Authors* of the relevant journal for any special instructions relating to CIF submission.

PLATON version of 06/01/2024; check.def file version of 05/01/2024

Datablock 3 - ellipsoid plot

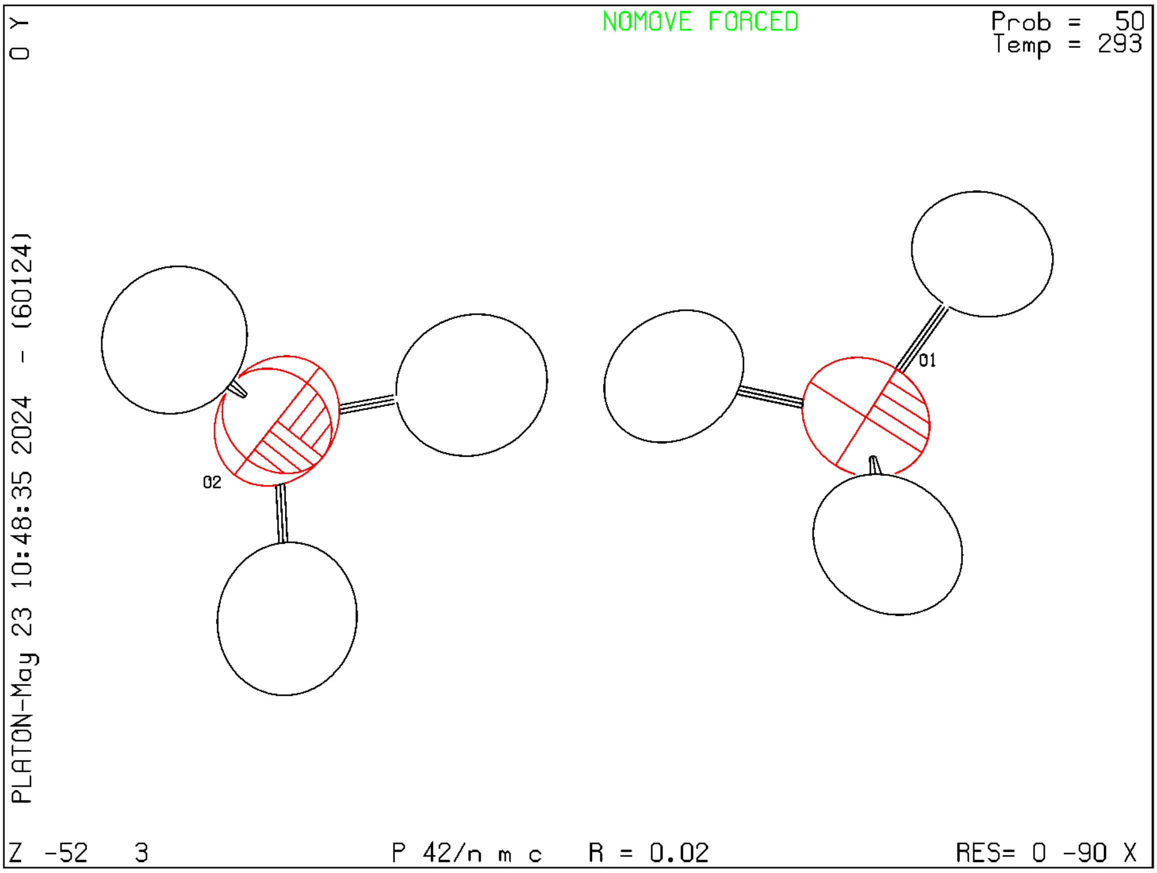

Supplement: Supplementary file 1 [file 3_checkcif.pdf › cif_checkcif/ice/B3LYP/3_checkcif.pdf]
