# Supplementary material for: Towards improved accuracy of Hirshfeld atom refinement with an alternative electron density partition
Source: IUCrJ. 2025 Jan 1;12(Pt 1):74–87. doi: 10.1107/S2052252524011242 (PMC11707693; doi:10.1107/S2052252524011242)

checkCIF/PLATON report

Structure factors have been supplied for datablock(s) oxa07

THIS REPORT IS FOR GUIDANCE ONLY. IF USED AS PART OF A REVIEW PROCEDURE FOR PUBLICATION, IT SHOULD NOT REPLACE THE EXPERTISE OF AN EXPERIENCED CRYSTALLOGRAPHIC REFEREE.

No syntax errors found. CIF dictionary Interpreting this report

Datablock: oxa07

Bond precision:	C-C = 0.0002 A		Wavelength=0.71073
Cell:	a=6.0950(5)	b=3.4881(3)	c=11.8462(9)
	alpha=90	beta=103.842(3)	gamma=90
Temperature:	100 K		

	Calculated	Reported
Volume	244.54(3)	244.54(4)
Space group	P 21/c	P 1 21/c 1
Hall group	-P 2ybc	-P 2ybc
Moiety formula	C2 H2 O4, 2(H2 O)	C2 H6 O6
Sum formula	C2 H6 O6	C2 H6 O6
Mr	126.07	126.07
Dx,g cm-3	1.712	1.712
Z	2	2
Mu (mm-1)	0.181	0.181
F000	132.0	132.1
F000’	132.13	
h,k,lmax	14,8,27	13,7,26
Nref	3095	2499
Tmin,Tmax	0.966,0.975	
Tmin’	0.966	

Correction method= Not given

Data completeness= 0.807 Theta(max)= 54.810

R(reflections)= 0.0139(2499) wR2(reflections)= 0.0386(2499)

S = 1.140 Npar= 64

The following ALERTS were generated. Each ALERT has the format
test-name_ALERT_alert-type_alert-level.
Click on the hyperlinks for more details of the test.

Alert level B

```
PLAT355_ALERT_3_B Long   O-H (X0.82,N0.98A)  O1        - H1        .        1.08 Ang.
PLAT417_ALERT_2_B Short Inter D-H..H-D       H1        ..H2        .        2.01 Ang.
                                                       x,y,z  =      1_555 Check
PLAT417_ALERT_2_B Short Inter D-H..H-D       H1        ..H3        .        2.06 Ang.
                                                       x,y,z  =      1_555 Check
PLAT934_ALERT_3_B Number of (Iobs-Icalc)/Sigma(W) > 10 Outliers ..          2 Check
               -3  3  3,   0  0 18,
```

Alert level C

```
PLAT029_ALERT_3_C _diffrn_measured_fraction_theta_full value Low .      0.960 Why?
PLAT042_ALERT_1_C Calc. and Reported MoietyFormula Strings  Differ     Please Check
              Calc: C2 H2 O4, 2(H2 O)
              Rep.: C2 H6 O6
PLAT911_ALERT_3_C Missing FCF Refl Between Thmin & STh/L=    0.600         17 Report
                6  2  0,  -1  1  1,  -1  3  1,  -5  3  2,  -2  0  2,   2  0  2,
                4  0  2,  -1  4  3,   0  2  3,   1  3  3,  -2  0  4,   1  0  4,
               -5  2  9,  -4  1 10,  -1  1 10,  -5  1 11,   1  1 11,
PLAT913_ALERT_3_C Missing # of Very Strong Reflections in FCF ....          4 Note
               -1  1  1,   2  0  2,  -2  0  4,   1  0  4,
```

Alert level G

```
PLAT778_ALERT_2_G Check O..H..X Bond in CIF: H1       --O3              1.41 Ang.
PLAT881_ALERT_1_G No Datum for _diffrn_reflns_av_R_equivalents ...     Please Do !
PLAT883_ALERT_1_G No Info/Value for _atom_sites_solution_primary .     Please Do !
PLAT910_ALERT_3_G Missing # of FCF Reflection(s) Below Theta(Min).          1 Note
                1  0  0,
PLAT912_ALERT_4_G Missing # of FCF Reflections Above STh/L=  0.600        574 Note
PLAT940_ALERT_3_G Fsqd Refinement With I > n * Sigma(I) Only .....     Please Check
PLAT961_ALERT_5_G Dataset Contains no Negative Intensities .......     Please Check
PLAT969_ALERT_5_G The 'Henn et al.' R-Factor-gap value ..........       1.21 Note
              Predicted wR2: Based on SigI**2  3.18 or SHELX Weight  3.43
PLAT977_ALERT_2_G Check Negative Difference Density on H2        .      -0.29 eA-3
PLAT977_ALERT_2_G Check Negative Difference Density on H3        .      -0.30 eA-3
PLAT978_ALERT_2_G Number C-C Bonds with Positive Residual Density.          0 Info
PLAT979_ALERT_1_G NoSpherA2 Scattering Factors Used .............      Please Note
```

0 **ALERT level A** = Most likely a serious problem - resolve or explain
4 **ALERT level B** = A potentially serious problem, consider carefully
4 **ALERT level C** = Check. Ensure it is not caused by an omission or oversight
12 **ALERT level G** = General information/check it is not something unexpected

4 ALERT type 1 CIF construction/syntax error, inconsistent or missing data
6 ALERT type 2 Indicator that the structure model may be wrong or deficient
7 ALERT type 3 Indicator that the structure quality may be low
1 ALERT type 4 Improvement, methodology, query or suggestion
2 ALERT type 5 Informative message, check

It is advisable to attempt to resolve as many as possible of the alerts in all categories. Often the minor alerts point to easily fixed oversights, errors and omissions in your CIF or refinement strategy, so attention to these fine details can be worthwhile. In order to resolve some of the more serious problems it may be necessary to carry out additional measurements or structure refinements. However, the purpose of your study may justify the reported deviations and the more serious of these should normally be commented upon in the discussion or experimental section of a paper or in the "special_details" fields of the CIF. checkCIF was carefully designed to identify outliers and unusual parameters, but every test has its limitations and alerts that are not important in a particular case may appear. Conversely, the absence of alerts does not guarantee there are no aspects of the results needing attention. It is up to the individual to critically assess their own results and, if necessary, seek expert advice.

Publication of your CIF in IUCr journals

A basic structural check has been run on your CIF. These basic checks will be run on all CIFs submitted for publication in IUCr journals (*Acta Crystallographica*, *Journal of Applied Crystallography*, *Journal of Synchrotron Radiation*); however, if you intend to submit to *Acta Crystallographica Section C* or *E* or *IUCrData*, you should make sure that full publication checks are run on the final version of your CIF prior to submission.

Publication of your CIF in other journals

Please refer to the *Notes for Authors* of the relevant journal for any special instructions relating to CIF submission.

PLATON version of 06/01/2024; check.def file version of 05/01/2024

Datablock oxa07 - ellipsoid plot

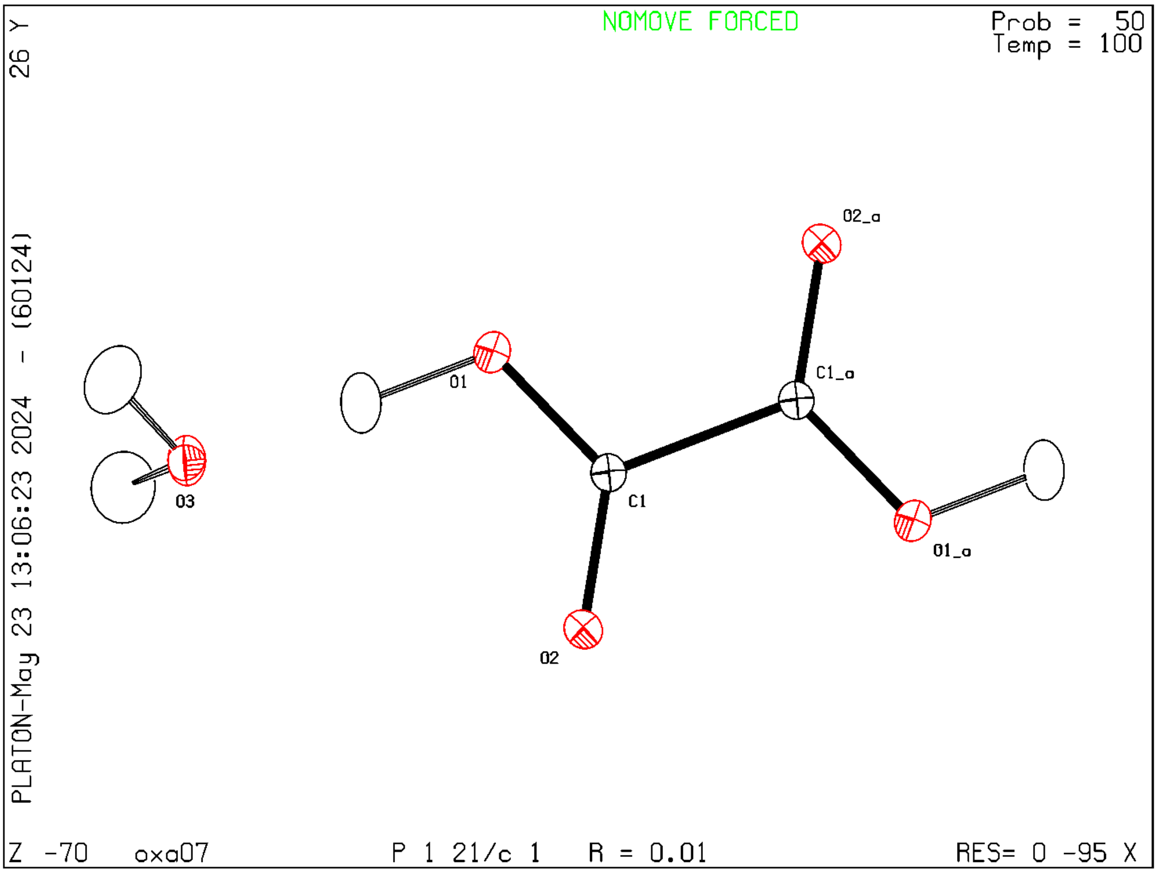

Supplement: Supplementary file 1 [file m-12-00074-sup1.zip › cif_checkcif/oxalic_acid_dihydrate/MP2/4_checkcif.pdf]
